# Osteohistological insight into the growth dynamics of early dinosaurs and their contemporaries

**DOI:** 10.1371/journal.pone.0298242

**Published:** 2024-04-03

**Authors:** Kristina Curry Rogers, Ricardo N. Martínez, Carina Colombi, Raymond R. Rogers, Oscar Alcober

**Affiliations:** 1 Biology and Geology Departments, Macalester College, St. Paul, Minnesota, United States of America; 2 Instituto y Museo de Ciencias Naturales, Universidad Nacional de San Juan, San Juan, Argentina; 3 CIGEOBIO - Centro de Investigaciones de la Geósfera y Biósfera, Consejo Nacional de Investigaciones Científicas y Técnicas - Universidad Nacional de San Juan, San Juan, Argentina; 4 Geology Department, Macalester College, St. Paul, Minnesota, United States of America; University of Gothenburg: Goteborgs Universitet, SWEDEN

## Abstract

Dinosauria debuted on Earth’s stage in the aftermath of the Permo-Triassic Mass Extinction Event, and survived two other Triassic extinction intervals to eventually dominate terrestrial ecosystems. More than 231 million years ago, in the Upper Triassic Ischigualasto Formation of west-central Argentina, dinosaurs were just getting warmed up. At this time, dinosaurs represented a minor fraction of ecosystem diversity. Members of other tetrapod clades, including synapsids and pseudosuchians, shared convergently evolved features related to locomotion, feeding, respiration, and metabolism and could have risen to later dominance. However, it was Dinosauria that radiated in the later Mesozoic most significantly in terms of body size, diversity, and global distribution. Elevated growth rates are one of the adaptations that set later Mesozoic dinosaurs apart, particularly from their contemporary crocodilian and mammalian compatriots. When did the elevated growth rates of dinosaurs *first* evolve? How did the growth strategies of the earliest known dinosaurs compare with those of other tetrapods in their ecosystems? We studied femoral bone histology of an array of early dinosaurs alongside that of non-dinosaurian contemporaries from the Ischigualasto Formation in order to test whether the oldest known dinosaurs exhibited novel growth strategies. Our results indicate that the Ischigualasto vertebrate fauna collectively exhibits relatively high growth rates. Dinosaurs are among the fastest growing taxa in the sample, but they occupied this niche alongside crocodylomorphs, archosauriformes, and large-bodied pseudosuchians. Interestingly, these dinosaurs grew at least as quickly, but more continuously than sauropodomorph and theropod dinosaurs of the later Mesozoic. These data suggest that, while elevated growth rates were ancestral for Dinosauria and likely played a significant role in dinosaurs’ ascent within Mesozoic ecosystems, they did not set them apart from their contemporaries.

## Introduction

Bone microstructural data provide an archive of the phylogenetic, ontogenetic, functional, and environmental factors that influence the paleobiology of fossil vertebrates [e.g., 1–5]. These histological data include details of primary bone protein, mineral, and vascular organization, density and distributions of osteocyte lacunae, punctuation of growth recorded by modulations, annuli, and/or Lines of Arrested Growth (LAGs), and signatures of bone remodeling. Among extant amniotes, experimental data have revealed distinctions in primary bone tissue patterns that reflect a continuum of bone growth rates that correspond to somatic growth rates [1, 5–14]. It is now firmly established that qualitative categorizations of primary bone microstructure allow for inferences relating to relative growth rates, ontogenetic stages, and growth strategies of fossil vertebrates [1–2, 4]

Bone tissue has been studied among a diverse assemblage of Late Triassic vertebrates including synapsids, archosauromorphs, archosauriformes, pseudosuchian (crocodile-line) archosaurs, non-dinosaurian avemetatarsalian/ornithodiran (bird-line) archosaurs, and a handful of isolated non-dinosaurian dinosauriforms and Late Triassic dinosaurs [1, 2, 4, 15, and references therein]. By necessity, sampling of these rare taxa is opportunistic with histological data often extracted from fragmentary material and/or disparate skeletal elements, thus making comparisons among analyses challenging. Some studies provided insight from multiple bones from an individual skeleton [e.g., 15, 16], while others were restricted to single, isolated, and sometimes unidentifiable skeletal elements [e.g., 17, 18]. Few studies have afforded an ontogenetic perspective among multiple skeletal elements from a single taxon [e.g., 19]. In spite of the challenges in comparing histology among disparate elements, different ontogenetic stages, and distant sampling locations, these landmark studies have shed light on the complicated growth dynamics of Triassic vertebrates. Triassic vertebrates likely exhibited a range of growth patterns as diverse as those observed in modern vertebrates, as well as patterns that sometimes diverge from the dominant pattern among their extant representatives [15–19].

**Fig 1:**
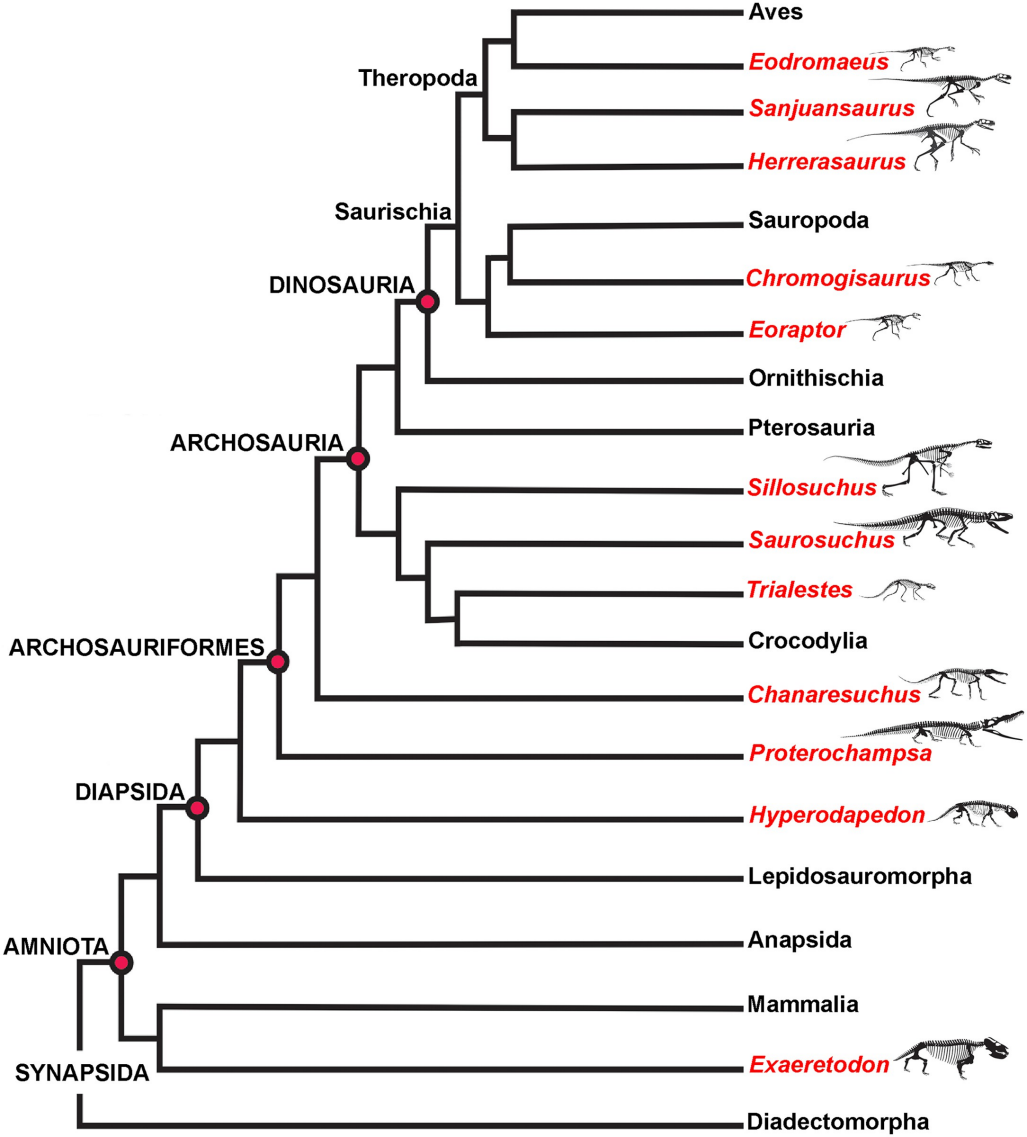
Simplified ischigualasto vertebrate phylogeny. The histological sample includes femoral mid-diaphyseal thin sections derived from each taxon indicated in red. The sample comprises a group of phylogenetically and ecologically diverse Late Triassic vertebrates, including five of the oldest known dinosaurs, all from a temporal interval spanning less than 2 million years within the Ischigualasto Formation of Argentina.

**Fig 2:**
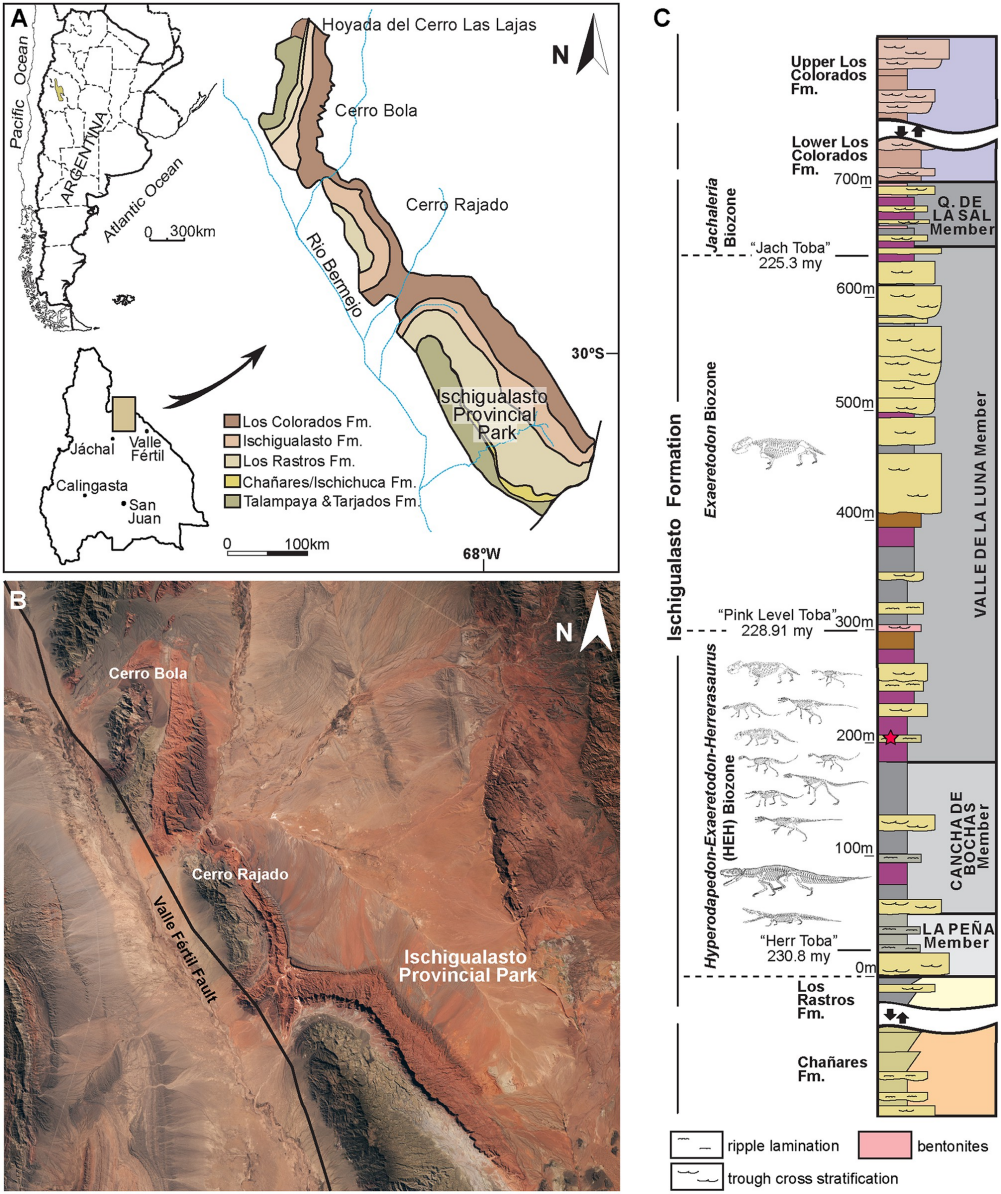
The late triassic ischigualasto formation, northwestern Argentina. A, Location and geological map of the Ischigualasto-Villa Unión Basin. B, Primary outcrop of the Ischigualasto Basin, including Ischigualasto Provincial Park, Cerro Bola, and Cerro Rajado. Landsat imagery courtesy of NASA Goddard Space Flight Center and U. S. Geological Survey. C, General stratigraphic section of the Ischigualasto Formation showing the stratigraphic positions of radiometric ages and the *Hyperodapedon-Exaeretodon-Herrerasaurus* (HEH), *Exaeretodon* (E), and *Jachaleria* (J) biozones. All the sampled vertebrates come from within the HEH biozone, deposited between 230.8 and 228.9 Ma. All but *Eodromaeus* come from the Cancha de Bochas Member. The stratigraphic position of *Eodromaeus* is indicated by the red star, at the base of the Valle de la Luna Member, and within the HEH biozone.

Advancing our current understanding of growth dynamics in relation to Triassic vertebrates requires a comparative, systematic sampling of homologous elements within a clearly resolved phylogenetic, temporal, and paleoenvironmental context [1, 2, 4, 5, 17, 18]. Here we document femoral histology for a group of phylogenetically and ecologically diverse Late Triassic vertebrates (Fig 1; Table 1). Our sample includes five of the oldest known dinosaurs and an array of their non-dinosaurian contemporaries, all from a narrow temporal interval within the Ischigualasto Formation of Argentina (Fig 2) [20]. Our study is among the first to approach the analysis of fossil vertebrate bone tissue from an “ecosystem-level” perspective, with basic constraints of taxonomic, anatomical, functional, paleoenvironmental, and temporal contexts, all affording a clearer path to direct comparison among disparate taxa. We investigated two key questions: 1) What is the variability in femoral bone histology among members of this pivotal Late Triassic paleofauna? and 2) Do our histological results support the hypothesis that the oldest known dinosaurs already exhibited the elevated growth dynamics characteristic of later Mesozoic dinosaurs?

**Table 1:** Specimens sampled for histological analysis in this study. All samples were derived from femoral mid-shafts in areas devoid of bone scars indicative of muscle attachments, including for those samples indicated with an * in which the element was partially preserved. Samples extracted from holotypes that consist of a single individual are indicated by a superscript H. Except for these singleton holotypes, most samples were drawn from larger specimens than those previously studied. Samples for *Hyperodapedon* and *Herrerasaurus* were extracted from the largest available specimens, which are equivalent in size to previously studied elements [15–19]. Data on habitat, diet, and estimated body mass are derived from Martínez and colleagues [6,7]. Institutional Abbreviations: PVSJ (División de Paleontología de Vertebrados del Museo de Ciencias Naturales y Universidad Nacional de San Juan, San Juan, Argentina).

Taxon	Specimen Number	Femur Length (cm)	Habitat	Diet	Mass (kg)
**Archosauromorpha**					
*Hyperodapedon sanjuanensis*	PVSJ 574	21.1	Terrestrial	Herbivore	25–250 kg
**Archosauriformes**					
*Proterochampsa barrionuevoi*	PVSJ 606	18.7	Aquatic	Carnivore	25–250 kg
*Pseudochampsa ischigualastensis* ^H^	PVSJ 567	18.1	Aquatic	Carnivore	<25 kg
**Crurotarsi**					
*Sillosuchus longicervix* ^H^	PVSJ 085	46.5	Terrestrial	Carnivore?	25–250 kg
*Saurosuchus galilei*	PVSJ 047	28.5*	Terrestrial	Carnivore	>250 kg
***Crocodylomorpha***					
*Trialestes romeri*	PVSJ 368	42.3	Terrestrial	Carnivore	25–250 kg
**Dinosauria**					
**Saurischia**					
**Theropoda**					
*Eodromaeus murphi* ^H^	PVSJ 561	16.0	Terrestrial	Carnivore	<25 kg
**Sauropodomorpha**					
*Eoraptor lunensis*	PVSJ 559	14.0*	Terrestrial	Omnivore?	< 25 kg
*Chromogisaurus novasi* ^H^	PVSJ 845	16.0*	Terrestrial	Herbivore	<25 kg
**Herrerasauridae**					
*Herrerasaurus ischigualastensis*	PVSJ 614	24*	Terrestrial	Carnivore	25–250 kg
*Sanjuansaurus gordilloi* ^H^	PVSJ 605	39.5*	Terrestrial	Carnivore	25–250 kg
**Cynodontia**					
*Exaeretodon argentinus*	PVSJ 38–2002	21.7	Terrestrial	Herbivore	25–250 kg

## Qualifying relative growth dynamics with bone histology

**Fig 3:**
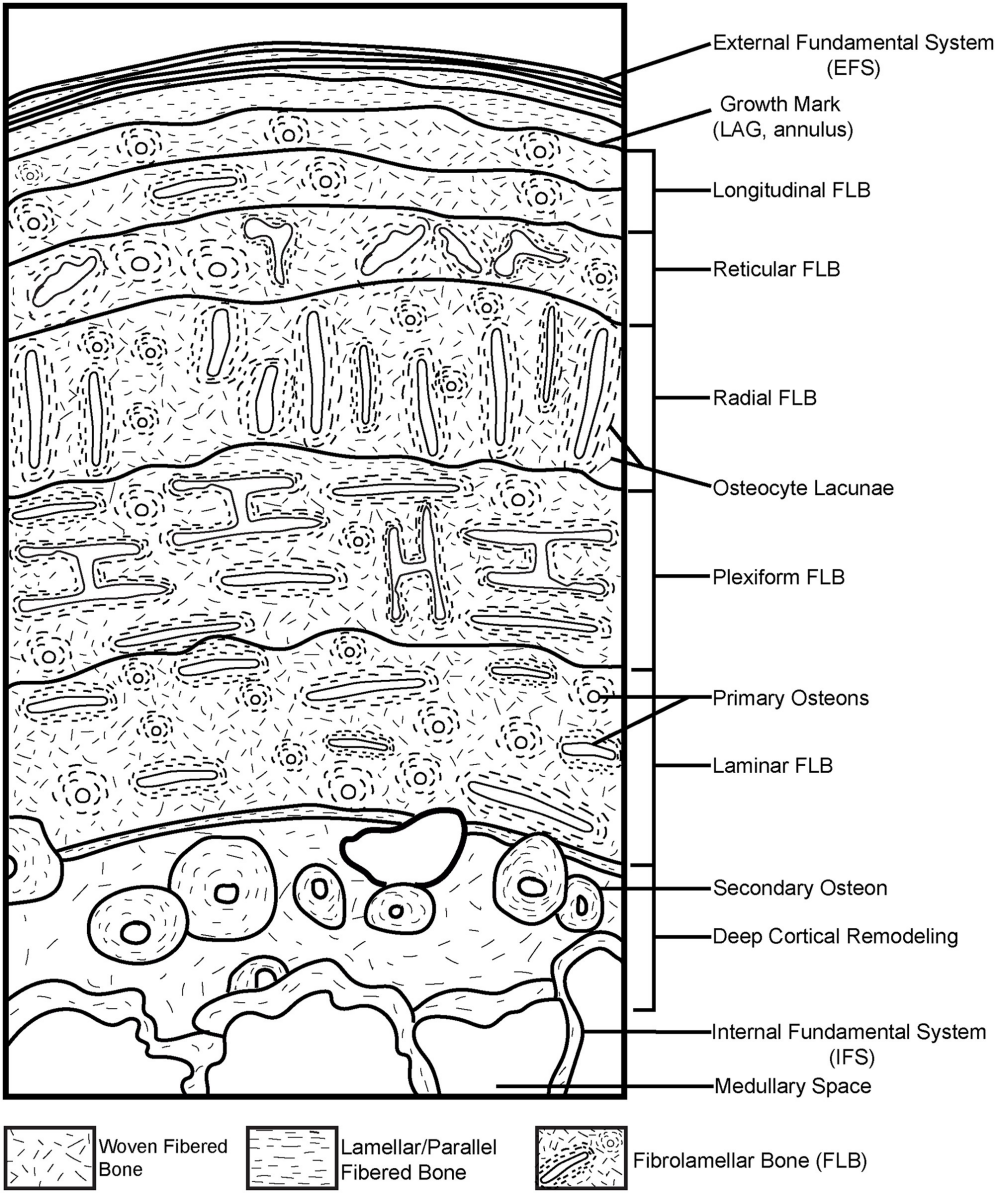
Schematic representation of a long bone cortex in cross-section. The degree of vascularization, the orientation of vascular canals (i.e., longitudinal, laminar, radial, reticular, and plexiform orientations), and the degree of organization of the collagenous matrix (e.g., woven, lamellar, or parallel fibered) directly reflect relative, local bone depositional rates. Growth marks, including LAG and annuli, indicate intervals in which appositional growth decelerates significantly and/or stops completely. These growth slowdowns and stoppages can occur annually, irregularly, and/or at the termination of appositional growth once somatic maturity is attained. Secondary remodeling includes erosion and redeposition of bone within trabeculae, at the border of cortical bone with the medullary cavity (where it may form an IFS) and/or within compact bone tissue, where it is recognized by erosion rooms, secondary osteons and dense Haversian bone tissue. Schematized structures may not all necessarily be found together within a single section [1, 2, 4, 5 and included references].

Empirical data among living vertebrates supports the hypothesis, first articulated in studies of fossils nearly 200 years ago [1–14, 21–26], that bone tissue typologies are the product of their depositional rates, which are, in turn, intimately linked to somatic growth rates and basal metabolic rates. These studies underscore the intricacies of bone microstructure and the complexities of quantifying patterns, particularly among fossil vertebrates. Different primary bone tissue types can be deposited at a range of possible, and sometimes overlapping, absolute rates [9, 10]. The same tissue types can be deposited at variable rates, even within different regions of the same skeletal element [e.g., 7, 8]. In addition, different skeletal elements from single skeletons grow distinctively within their unique functional environments [e.g., a weight bearing femur vs. a non-weight-bearing rib] [e.g., 8–10, 24, 24–29], which may also result in primary osteohistology and/or differential patterns of remodeling that produce inconsistent results when the goal is determining the absolute age of the sampled individual via skeletochronology [28–34]. These caveats make the determination of absolute growth rates and ages particularly challenging among fossil taxa. That said, when we control for functional environment, ontogenetic stage, taxonomic identity, and paleoenvironmental context, the analysis of bone microstructural organization among individuals and/or taxa can illuminate comparative *relative* growth dynamics, particularly among taxa derived from the same ecosystem [18, 28]. Such controls on sampling make it possible to glean more specific comparisons among taxa, and are required if our goals include the analysis of distinctions in growth dynamics among species. Below, we review just a few of these different bone organizational patterns as they relate to the interpretations of relative growth dynamics of Ischigualasto Formation vertebrates (Fig 3).

Bone tissue types signaling relatively slow appositional rates include lamellar-fibered bone (LFB), deposited at the slowest known growth rates, and parallel-fibered bone (PFB), deposited at intermediate rates [1, 2, 5]. LFB is formed when collagen and bone mineral align during bone deposition as a series of stacked, well-differentiated 5–7 micron thick lamellae composed of fibers that are parallel within each lamella, but with shifting orientations between adjacent lamellae. In contrast, PFB is diagnosed by primary bone that exhibits a highly organized, unidirectional, parallel fiber and osteocyte lacuna configuration, but with occasional small pockets of more disorganized primary bone occurring within interstitial spaces. In both tissue types, vascular density and rates of bone remodeling tend to be comparatively low, while growth marks, including annuli and Lines of Arrested Growth (LAG) occur more regularly throughout the primary cortex [e.g., 1–5, 30]. Annuli are recorded by a microstructural transition to more organized LFB and/or PFB in which osteocyte lacunae are aligned in parallel rows. These features indicate a temporary decrease in appositional growth rate relative to surrounding tissues. LAG often accompany annuli, and are diagnosed as circumferential cementing lines that indicate a temporary but complete cessation of appositional growth (Fig 3, see below). When stacked layers of circumferential annuli and/or LAG occur at the external margin of bone cross-sections, often in the context of avascular primary bone, they are termed the “External Fundamental System (EFS)” or the “Outer Circumferential Layer (OCL)” and signal the end of robust appositional growth and attainment of adult size [1–5] (Fig 3). Similar slow-growing, avascular deposits of LFB may also occur as a lining of the medullary space. These deposits, termed the “Internal Fundamental System (IFS),” indicate medullary drift, a process of centripetal deposition of bone tissue linked to episodic remodeling of the peri-medullar cortex [1–5, see below]. PFB and LFB characterize the majority of primary appositional growth among small vertebrates including extant lissamphibians and squamates, various extant and fossil mammals [5, 35], and in the majority of large bodied non-avian extinct and extant reptiles excluding most non-avian dinosaurs, but including some birds [1–5, 28, 36–38]. LFB also forms a significant component of bone tissue in relatively fast-growing taxa because it builds both primary and secondary osteons (see below) and, along with PFB, contributes to the formation of intracortical growth marks (annuli and LAGs), as well as the Internal Fundamental System (IFS) and the External Fundamental System (EFS) [1, 2, 5, 22–23, 39–42].

The primary bone tissue type that is most commonly associated with relatively faster bone apposition is fibrolamellar bone [FLB; 1, 2, 5, 43, 44]. FLB is a composite primary bone tissue type formed by the rapid sub-periosteal accretion of woven fibered bone in a loose, disorganized scaffold of fine cancellous bony trabeculae surrounding dense networks of periosteally-derived primary capillaries that are subsequently infilled by centripetally deposited, slower-growing LFB to form primary osteons (Fig 3). Primary osteonal networks occur in a variety of patterns (e.g., longitudinal, reticular, radial, circular, laminar, and plexiform) (Fig 3). In these vascular networks, increasing anastomoses and complexity are linked to increasing relative growth rates [1, 2, 5, 4, 5, 6–13, 23–25]. LAG and annuli also occur in FLB. Peripheral growth marks forming an EFS signal the termination of major appositional growth, while intracortical growth marks have been linked to a thermometabolic strategy for energy conservation during unfavorable seasons across environmental gradients among extant large-bodied ruminants with FLB [45]. FLB dominates the primary bone appositional signal and is thought to indicate high sustained metabolic activity in most moderate- and large-bodied mammals, many extant birds, and non-avian dinosaurs [e.g., 1–13, 22–25, 35, 45–51]. FLB can also occur in juvenile ontogenetic stages for extant taxa typically known for slower overall growth histories, but in these cases, it represents a much smaller proportion of overall appositional bone growth than LFB or PFB [27, 52, 53].

Primary bone tissue also includes cyclically deposited growth marks (LAG and annuli), which are useful in the assessment of absolute age in at least some extant and extinct vertebrates [see 4, 31 for review]. That said, as we explore intraskeletal variation in LAG among extant vertebrates of known age, a number of complications have diminished the efficacy of LAG counts as reliable indicators of absolute age [28–33]. Not all organisms, and especially not those that are particularly fast-growing, deposit cyclical LAG throughout ontogeny [28–33]. Even if they do, bone remodeling and medullary drift may lead to obliteration of growth marks as ontogeny proceeds. Moreover, LAG and annuli also form in organisms that normally grow continuously, but pause when they experience extrinsic stressors, such as resource limitations during severe drought. In these cases LAG may signify seasons of stress more faithfully than absolute age [45–48]. In addition, some groups, including early dinosauromorphs [54, 55]; some sauropods [49, 50, 56, 57] and some hadrosaurs [58] do not appear to deposit LAG or do so seemingly haphazardly. Thus, the absence of clearly defined growth marks does not necessarily indicate less than a single year of growth. Instead, an absence of LAG/annuli may reflect a lengthier temporal interval of continuous growth [1–5, 11–14, 28–33, 45, 47, 53–59].

Remodeling is an essential component of bone biology and can occur (1) within trabecular bone, (2) at the border of cortical bone with the medullary cavity (where it may form the IFS) and/or (3) within compact cortical bone [1, 2, 4, 5, 56, 57]. Signatures of cortical bone remodeling include erosion rooms, which indicate the onset of bone resorption mediated by osteoclasts, and secondary osteons (also called Haversian osteons), which indicate the redeposition of LFB by osteoblasts within these erosional spaces (Fig 3) [1, 2, 5]. When multiple generations of secondary osteons occur at high density they overlap one another, obliterating underlying primary bone, to form dense Haversian tissue [1, 2, 5]. Variable degrees of secondary osteon development have been recorded in taxa as diverse as agnathans and mammals [5], although it tends to occur most frequently within the context of FLB when compared to PFB/LFB. That said, secondary osteons are rare in the long bones of lissamphibians and squamates [5, 30, 32–33, 37, 39–41].

Remodeling can obliterate signatures of primary bone deposition in earlier ontogeny and so, whenever possible, sampling of ontogenetic series is recommended [5]. When ontogenetic series are unavailable for study, as is often the case for fossil taxa, it is critical to compare homologous elements as well as homologous regions of long bones where remodeling might be more limited (e.g., mid-shaft diaphyses in areas devoid of muscle origin or insertion) [1–5; 17–18, 22–31, 56–57, 60–63]. Remodeling serves several functions: 1) repair of fatigue microfractures that accumulate over the course of ontogeny and compromise the structural integrity of bones; 2) microanatomical restructuring in response to mechanical loading demands; and 3) mineral storage and mobilization, fundamental to somatic growth and phosphocalcic homeostasis [5]. Each function is intimately linked to both metabolism and ontogenetic age; with higher metabolism and/or increasing age, bone remodeling becomes increasingly pervasive [1–5, 56, 60–63]. Cortical remodeling via secondary osteons, as well as medullary-focused remodeling (the IFS and trabecular reworking) can thus serve as reliable indicators of relative ontogenetic status [1–5, 56, 60–63].

## Geological context: The ischigualasto formation

The Ischigualasto Formation in San Juan Province, central-western Argentina, is a richly fossiliferous fluvial succession within the Triassic Ischigualasto-Villa Unión basin (Fig 2). The unit preserves an exceptional record of Late Triassic vertebrates, including some of the earliest known dinosaurs, archosauromorphs, pseudosuchian archosaurs, therapsids, and amphibians (Figs 1 and 2, Table 1) [64–70]. The Ischigualasto fossil record is contextualized with abundant sedimentologic, paleoclimatic, geochronologic, and taphonomic evidence [20, 65, 67, 69, 71–76]. The unit and its vertebrate fauna are of primary importance for investigating vertebrate paleobiology and evolution during Late Triassic faunal turnover. Fluvial sandstones, siltstones, and mudstones that represent channels and their associated floodplains dominate the unit, which is subdivided into four members based on depositional architecture and paleosol characteristics: the La Peña, Cancha de Bochas, Valle de la Luna, and Quebrada de la Sal members (Fig 2C) [20, 67, 73, 75, 76]. The vast majority of Ischigualasto vertebrate fossils are recovered from fluvial deposits within the Cancha de Bochas Member, which records an arid to semiarid, highly seasonal paleoclimate (Fig 2C) [20, 67, 69].

The Ischigualasto Formation is also divided from base to top into three abundance-based biozones that span multiple geological units: the *Hyperodapedon-Exaeretodon-Herrerasaurus* (HEH), *Exaeretodon* (E), and *Jachaleria* (J) biozones (Fig 2C) [67]. The HEH biozone spans the La Peña and Cancha de Bochas Members, and the base of the Valle de la Luna Member of the Ischigualasto Formation. All sampled taxa except *Eodromaeus* come from the Cancha de Bochas portion of the HEH biozone; the *Eodromaeus* sample is derived from the base of the Valle de la Luna portion within the HEH biozone (Fig 2C). The HEH biozone is dominated by mid-sized non-dinosaurian herbivores; the earliest known dinosaurs represent ~11% of recovered vertebrate taxa, with dinosaurs occupying terrestrial carnivore and small-bodied omnivore and/or herbivore niches [67]. Vertebrate fossils recovered from the HEH biozone represent both attritional and mass mortality assemblages, presumably the result of both background mortality and intervals of drought-related mortality in an arid to semiarid climate [72–76]. ^40^Ar/^39^Ar dating of volcanic ash sampled from near the base and top of the formation produced ages of ~231.4 Ma and ~225.9 Ma, respectively [65]. Most recently, a new age derived from high precision CA-Tims U/Pb zircon dating indicates that a major paleoenvironmental perturbation and biotic crisis occurs at 228.91 ± 0.14 Ma, near the top of the Cancha de Bochas Member (Fig 1) [20], and capping our sampling interval (Fig 2C). These geochronological data constrain our histological sample from the HEH Biozone to a temporal interval of less than 2 million years.

## Materials and methods

**Fig 4:**
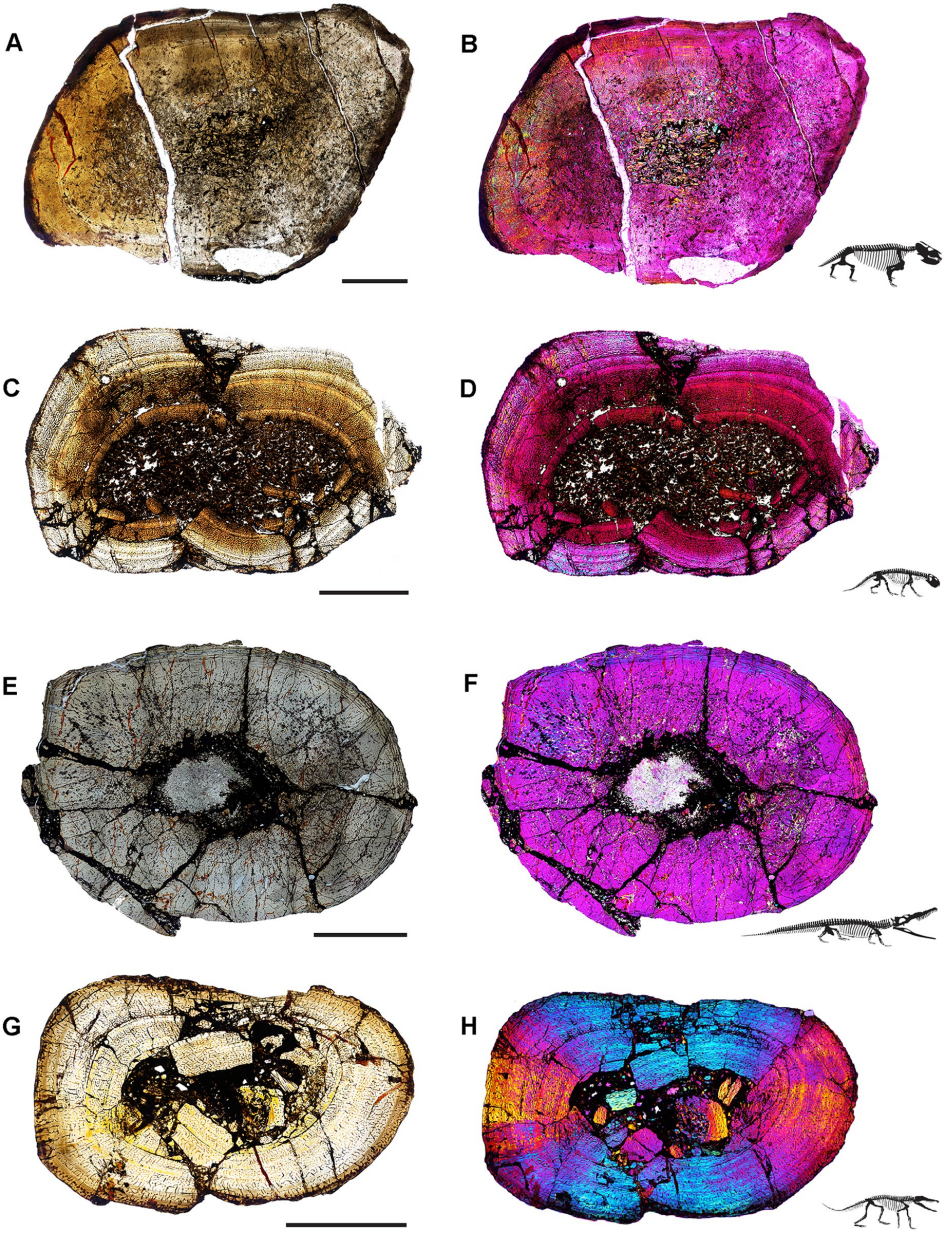
Ischigualasto Vertebrate Femoral Cross-Sections in Plane Polarized Light (PPL) and Cross-Polarized Light (XPL) with Lambda Compensator. For each taxon PPL images are on the left; XPL/lambda compensator images are on the right. Anterior is toward the top. **A** and **B**, *Exaeretodon* (PVSJ 38–2002); Scale 5 mm. **C** and **D**, *Hyperodapedon* (PVSJ 574); Scale = 10 mm. **E** and **F**, *Proterochampsa* (PVSJ 606); Scale = 5 mm. **G** and **H**, *Pseudochampsa* (PVSJ 567); Scale = 5 mm.

**Fig 5:**
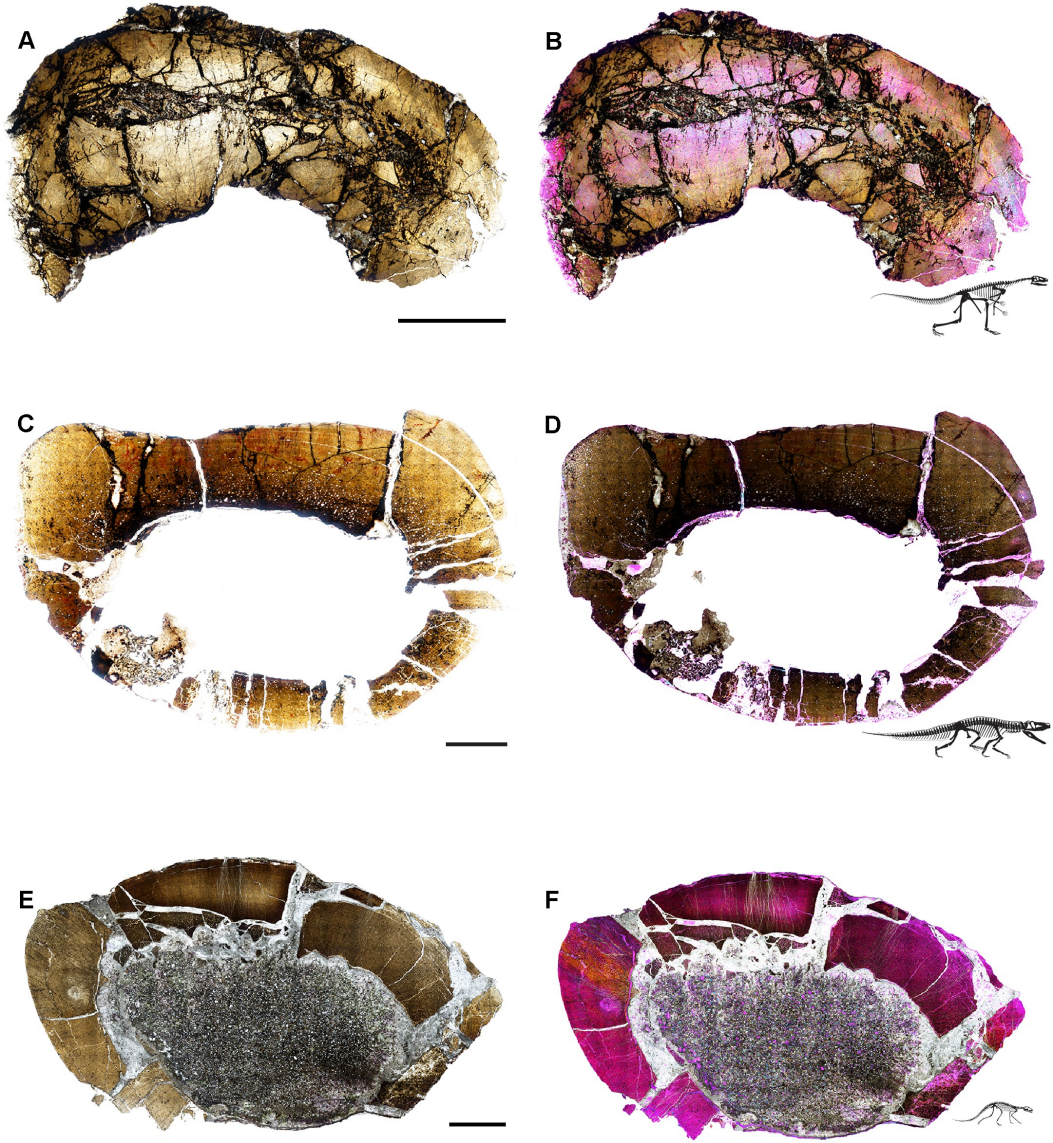
Ischigualasto Vertebrate Femoral Cross-Sections in Plane Polarized Light (PPL) and Cross-Polarized Light (XPL) with Lambda Compensator. For each taxon PPL images are on the left; XPL/lambda compensator images are on the right. Anterior is toward the top. **A** and **B**, *Sillosuchus* (PVSJ 085); Scale = 10 mm. **C** and **D**, *Saurosuchus* (PVSJ 047); Scale = 10 mm. **E** and **F**, *Trialestes* (PVSJ 368); Scale = 10 mm.

**Fig 6:**
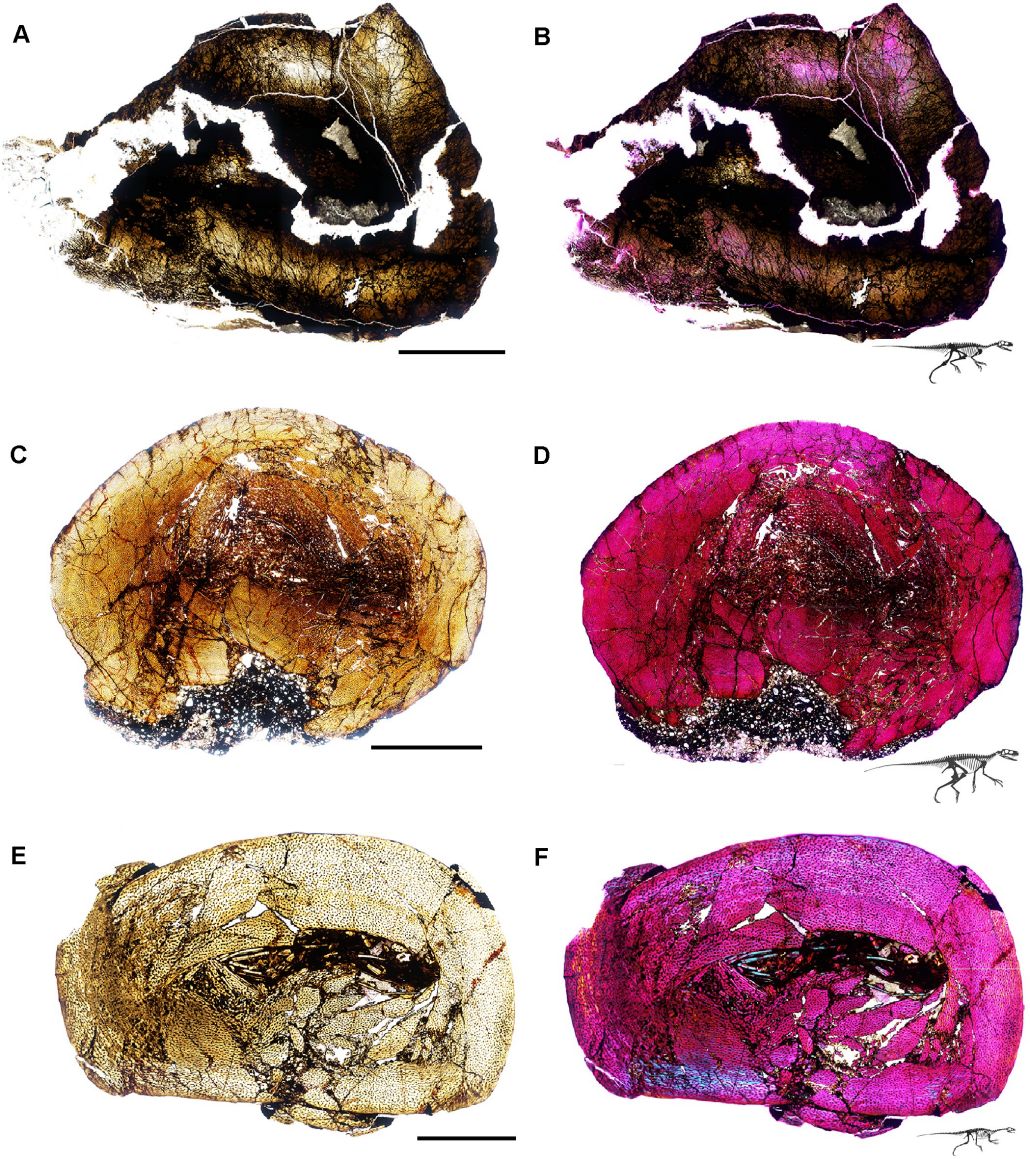
Ischigualasto Vertebrate Femoral Cross-Sections in Plane Polarized Light (PPL) and Cross-Polarized Light (XPL) with Lambda Compensator. For each taxon PPL images are on the left; XPL/lambda compensator images are on the right. Anterior is toward the top. **A** and **B**, *Sanjuansaurus* (PVSJ 605); Scale = 10 mm. **C** and **D**, *Herrerasaurus* (PVSJ 614); Scale = 10 mm. **E** and **F**, *Eodromaeus* (PVSJ 561); Scale = 3 mm.

**Fig 7:**
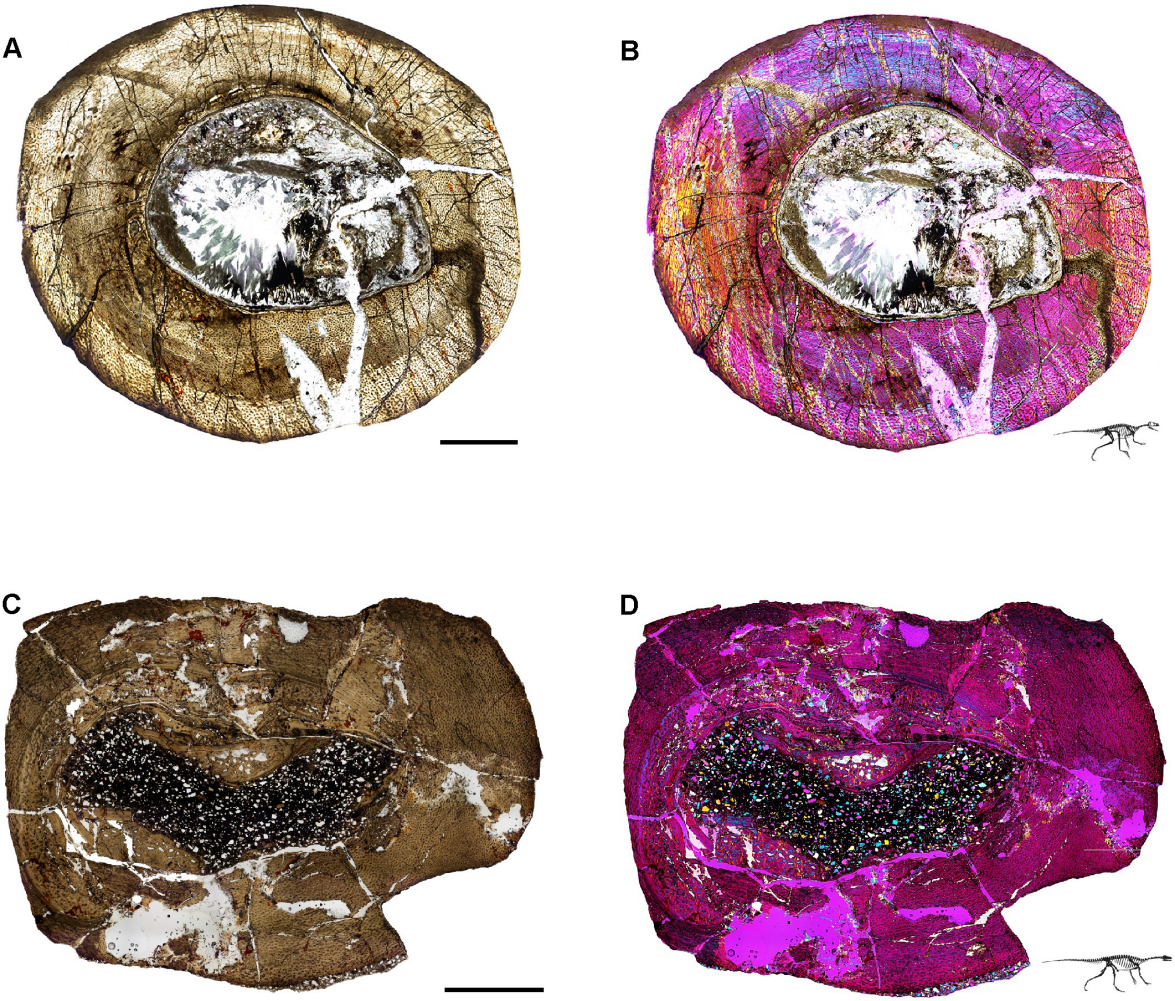
Ischigualasto Vertebrate Femoral Cross-Sections in Plane Polarized Light (PPL) and Cross-Polarized Light (XPL) with Lambda Compensator. For each taxon PPL images are on the left; XPL/lambda compensator images are on the right. Anterior is toward the top. **A** and **B**, *Eoraptor* (PVSJ 559); Scale = 3 mm. **C** and **D**, *Chromogisaurus* (PVSJ 845); Scale = 3 mm.

Our sample includes species spanning small (< 25 kg) to large body sizes (>250 kg), as well as herbivorous, carnivorous, and omnivorous taxa, bipeds and quadrupeds, and several taxa known only from holotype specimens (Fig 1; Table 1) [67]. Samples are derived from fossils curated in the División de Paleontología de Vertebrados del Museo de Ciencias Naturales y Universidad Nacional de San Juan, San Juan, Argentina (PVSJ), and consist of complete transverse sections of femoral mid-diaphyses for all taxa (Figs 4–7). We recognize that a sample limited to femora cannot fully represent the full range of tissues deposited in all parts of the skeleton throughout all ontogenetic stages. We also recognize that the diverse taxa in our sample include animals that represent a spectrum of locomotory biomechanics, from sprawlers (e.g., *Hyperodapedon*) to upright, bipedal cursors (e.g., *Eodromaeus*). It is plausible that these differences had some impact on bone histological organization. We have chosen to limit our sample to femora, in part, to minimize potential variations related to sampling of non-homologous elements. Sampling the same element in a similar location for every taxon helps to control for histological differences that might be driven by functional anatomy (e.g., weight-bearing hindlimbs vs. non-weight-bearing forelimbs) or even by the position of the thin section within an element (e.g., avoiding muscle attachment points or the trabecular-bone dominated ends of long bones). The femur is often subjected to less intensive secondary remodeling and records a more complete record of ontogenetic growth than other appendicular and axial skeletal elements [e.g., 1–5; 17, 18, 22–31, 56–57, 60–63; 77–79]. For these reasons, femora are commonly employed in histological studies of fossil and extant vertebrates. This increases the comparative power of a femoral sample—it affords a higher likelihood of direct comparison with published work and with potential future studies of femoral samples. For the few sampled Ischigualasto taxa for which ontogenetic series are known, we have also focused our sampling on the largest known individuals in hopes of capturing a longer histological archive of growth over time. This is particularly significant in light of demonstrated shifts in bone histology over the course of ontogeny [e.g., 1, 2, 5, 80]. Finally, limiting our sample to specimens derived from the HEH biozone, mostly within the Cancha de Bochas Member of the Ischigualasto Formation (Fig 2C) further reduces the possibility for observed histological distinctions driven by extrinsic environmental factors.

Our sample includes the basal dinosaurs *Chromogisaurus novasi* [81, 82], *Eodromaeus murphi* [83], *Eoraptor lunensis* [60, 82, 84], *Herrerasaurus ischigualastensis* [85, 86], and *Sanjuansaurus gordilloi* [87, 88]. Our sample also includes the herbivorous traversodontid cynodont *Exaeretodon argentinus* [89], the herbivorous rhynchosaurian archosauromorph *Hyperodapedon sanjuanensis* [90], and a suite of pseudosuchian archosaurs, including the carnivorous proterochampsians *Proterochampsa barrionuevoi* [91] and *Pseudochampsa ischigualastensis* [91, 92], the large-bodied carnivores *Sillosuchus longicervix* (a poposaurid) [93] and *Saurosuchus galilei* (a loricatan) [94–96], and the carnivorous crocodylomorph *Trialestes romeri* [97, 98] (Fig 1; Table 1). The histological descriptions provided here are the first for *Pseudochampsa*, *Saurosuchus*, *Sillosuchus*, *Trialestes*, and for all of the dinosaurs except *Herrerasaurus*. For other taxa, our samples build upon the work of our colleagues by adding new data from the femora of large-bodied individuals. Our sample includes histological data for five holotype specimens (Table 1). No permits were required for the described study, which complied with all relevant regulation. All specimens, along with their thin sections, are curated in the Museo de Ciencias Naturales at the Universidad de San Juan in San Juan, Argentina (PVSJ). Additional copies of thin-sections are housed at Macalester College in St. Paul, Minnesota, United States. High resolution versions of all included images and additional montaged and high magnification photomicrographs are also archived in MorphoBank (Project 4515).

Prior to sectioning, elements were measured, scanned, molded, cast, and photographed to document their original morphology. Mid-diaphyseal blocks 1–2 cm thick were extracted from each element using a high-speed diamond saw. Each bone block was prepared using traditional hard-tissue histology sampling techniques [2]. We studied elements with a petrographic microscope (Nikon Eclipse 50iPOL) in plane-polarized (PPL) and cross-polarized (XPL) light. We often utilized XPL light with a lambda compensator to visualize zones of anisotropic (LFB/PFB) and isotropic (WFB) bone mineral organization (e.g., Fig 4B). We obtained photomicrographs with this microscope and a Nikon DS-Fi1 digital camera (e.g., Fig 7). Composite images for each thin-section were compiled with NIS-Elements BR 4.20 (Figs 4–7).

## Osteohistological results in context

**Fig 8:**
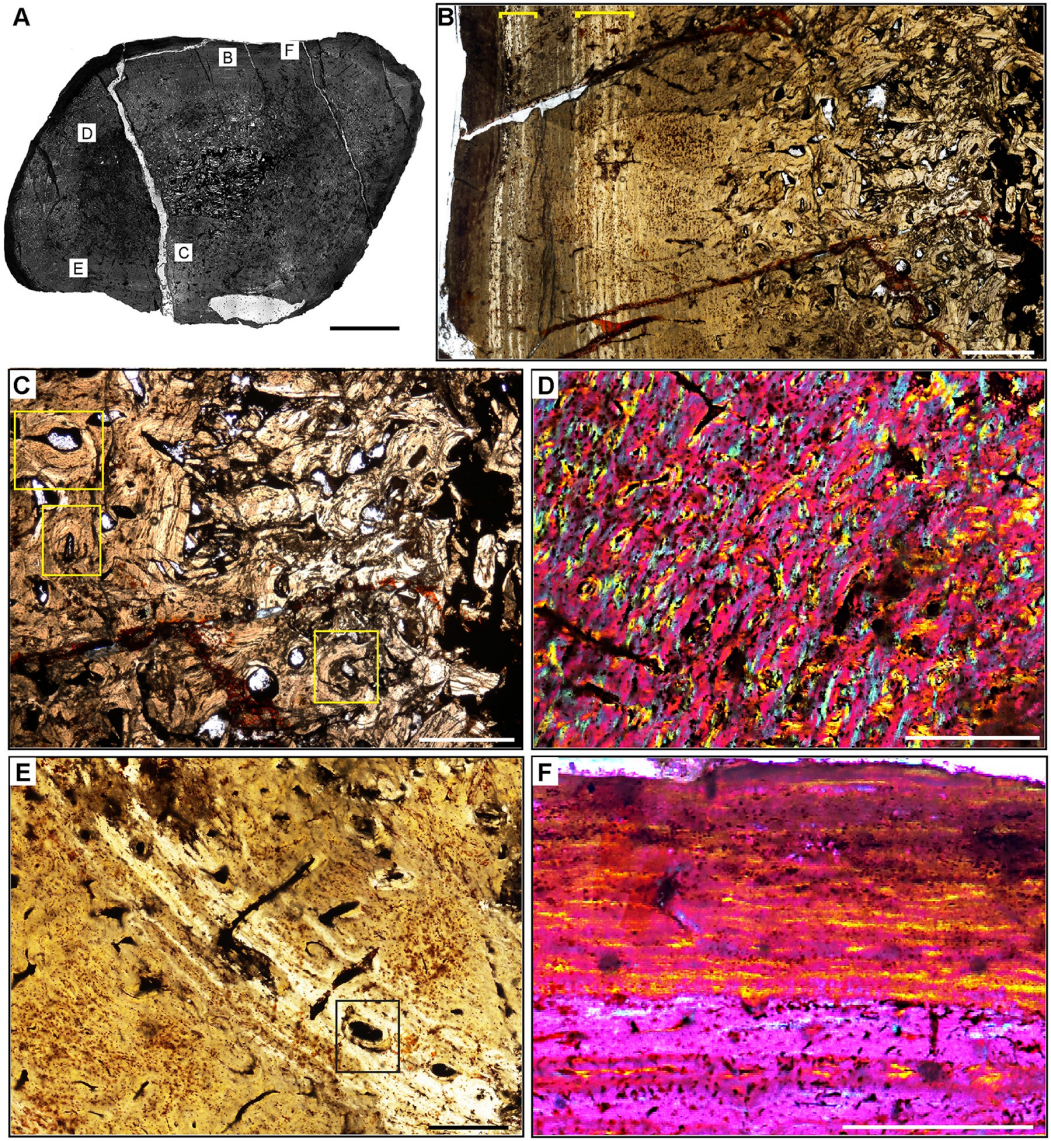
Femur histology of *Exaeretodon argentinus* PVSJ 38–2002. (**A**) General view of femoral histology in plane polarized light. Letters indicate positions of higher magnification photomicrographs B–F. Anterior is toward the top. Scale bar = 5 mm. (**B**) PPL image of a thickened, compacted cortex (left) that surrounds a small medullary space filled with bony trabeculae that have undergone multiple generations of endosteal remodeling (right). Several cycles of annuli and LAG are visible in the mid- and outer cortex (yellow brackets). The periosteal surface of the element is at the left. Scale bar = 5 mm. (**C**) PPL image highlighting secondary remodeling. Remodeling is indicated by both erosion rooms and secondary osteons (yellow rectangles), and is confined to perimedullar regions of the deep cortex. The single generation of secondary remodeling leaves persistent patches of primary bone tissue throughout the cross section. Scale bar = 250 microns. (**D**) XPL with lambda compensator image illustrates the highly vascularized fibrolamellar bone tissue characteristic of most cortical appositional growth in *Exaeretodon*. Bright pink areas of isotropic bone mineral organization highlight the woven bone component of the fibrolamellar complex. Turquoise and yellow areas reveal the more highly organized, anisotropic lamellar bone mineral organization within primary osteons of the fibrolamellar complex. In this view, primary vasculature is mostly longitudinal, with occasional circular and rare radial anastomoses. Scale bar = 500 microns. (**E)** PPL image of a broad zone of mid-cortical annuli and LAG. Rare secondary osteons occur between each annulus (black rectangle). Following deposition of these annuli (toward the bottom left corner of this image), appositional growth resumes, but with reduced primary vascularity dominated by mature longitudinal primary osteons. Scale bar = 500 microns. (**F**) XPL with lambda compensator image of periosteal surface. Here in the outermost cortex, bone tissue is nearly avascular and exhibits a significant increase in bone mineral organization to a lamellar bone matrix. The periosteal surface exhibits multiple stacked Lines of Arrested Growth (LAG) highlighted by yellow in this view. These LAG form the External Fundamental System (EFS) that signals the end of major appositional growth and the attainment of skeletal maturity. Scale bar = 500 microns.

**Fig 9:**
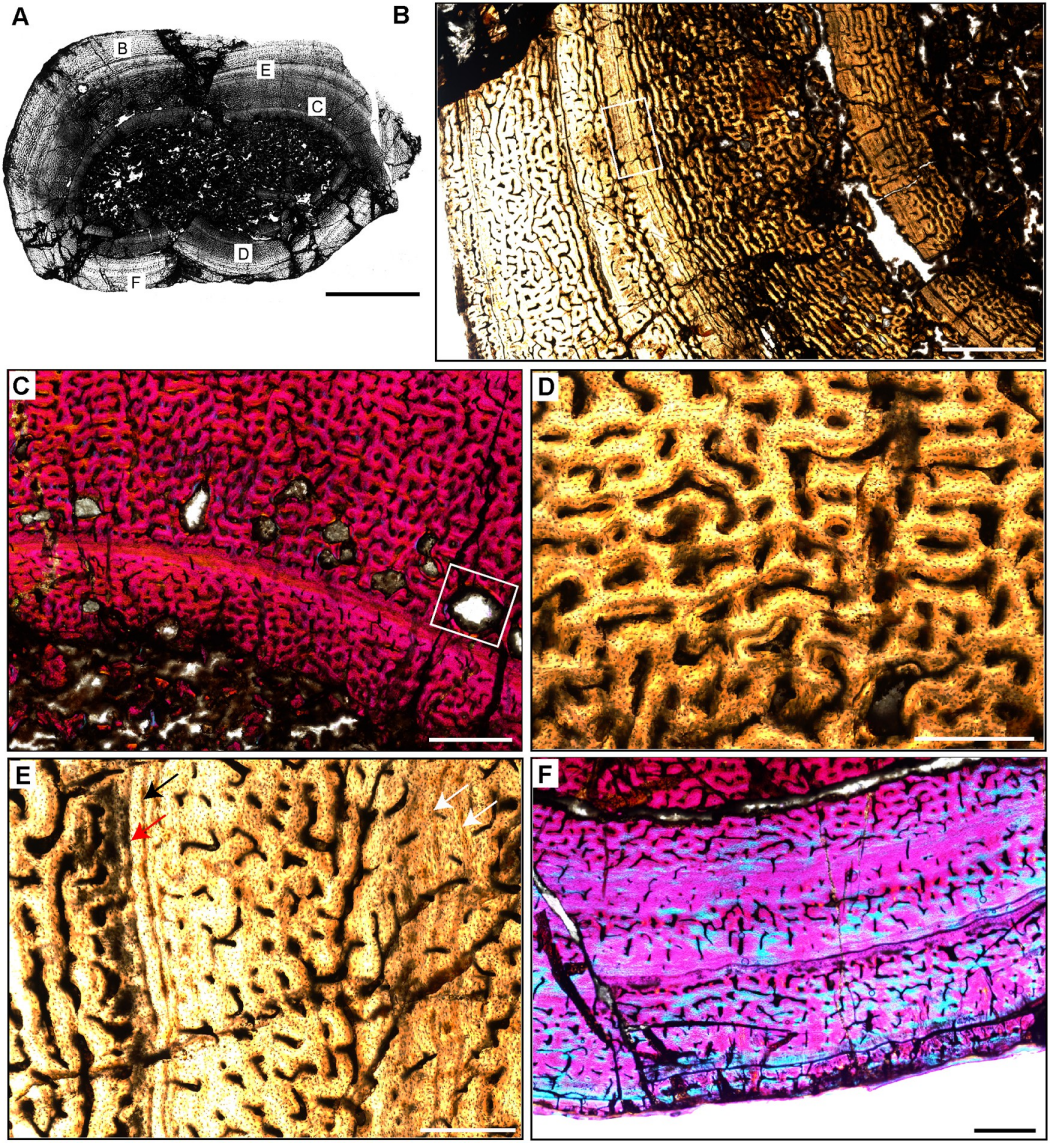
Femoral Histology of *Hyperodapedon sanjuanensis* PVSJ 574. (**A**) General view of femoral histology in plane polarized light (PPL). Letters indicate positions of higher magnification photomicrographs B–F. Anterior is toward the top. Scale bar = 10 mm. (**B**) PPL image of a thick highly vascularized cortex (left) that surrounds a medullary space that appears to be filled with broken bony trabeculae (right). Primary vascularity is high throughout most of the cross-section, though in the area indicated by the white rectangle a circumferential shift to more organized bone mineral signals a temporary reduction in bone apposition. Subsequently, additional cycles of annuli and LAG are visible nearer the periosteal surface (to the left). Scale bar = 1 mm. (**C**) XPL with lambda compensator image highlighting the onset of secondary remodeling in the deeper regions of the cortex. Remodeling is indicated by the presence of erosion rooms, a few of which exhibit centripetal deposition of lamellar bone indicating incipient formation of secondary osteons (white rectangle). Signatures of remodeling are mostly confined to the mid-cortex, except for one large erosion room in the outer cortex (seen in A). Scale bar = 1 mm. (**D**) PPL image highlighting the highly vascularized fibrolamellar bone tissue characteristic of the majority of cortical appositional growth in *Hyperodapedon*. In this region, primary osteons interweave in a reticular pattern. Scale bar = 500 microns. (**E)** PPL image of a typical mid-cortical growth cycle in *Hyperodapedon*. In this image, the deeper cortex is toward the right; the more superficial cortex is toward the left. At the left, faint annuli occur in the context of relatively lower vascularity and osteocyte lacunae that are arranged in parallel layers within a small region of PFB (white arrows). In the middle of this view, vascularity is high, reticular primary osteons are dominate, and occur within a woven bone context to form a typical fibrolamellar complex. Toward the left and more superficially, a reduction in relative bone appositional rate is recorded by a reduction in primary vascularity and a corresponding shift to more organized PFB, followed by deposition of an annulus (black arrow) and a LAG (red arrow). Following deposition of this LAG, the resumption of elevated primary bone depositional rate is heralded by the highly vascularized reticular FLB on the far left. Scale bar = 500 microns. (**F**) XPL with lambda compensator image from periosteal surface. Here in the outermost cortex, primary vascularity is reduced compared to the deeper and mid-cortex. A transition to more abundant PFB and/or LFB is recorded by the turquoise zones in this image, which indicate the isotropic nature of bone mineral in the outer cortex. The periosteal surface lacks evidence of an EFS. Scale bar = 1 mm.

**Fig 10:**
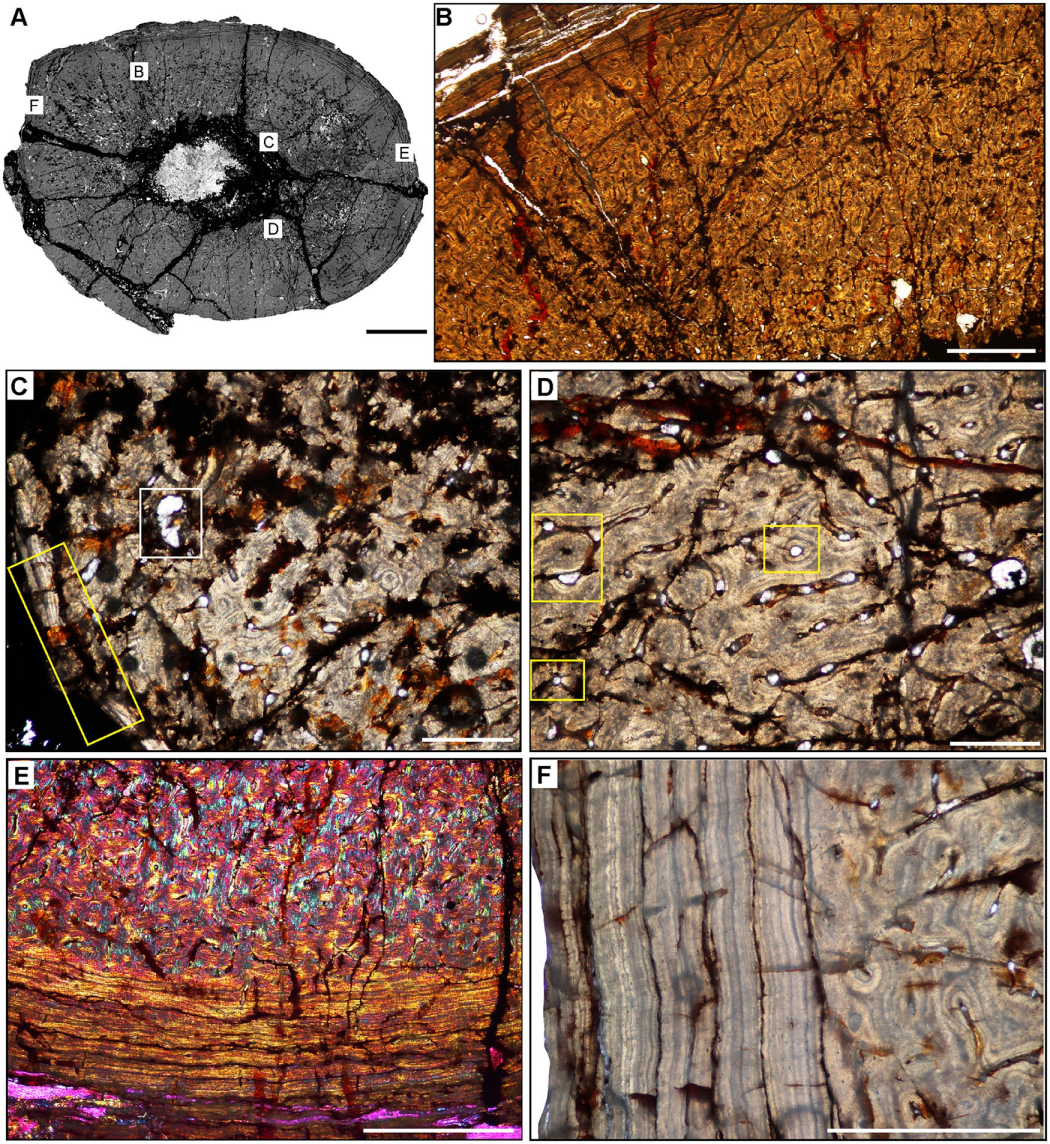
Femoral Histology of *Proterochampsa barrionuevoi* PVSJ 606. (**A**) General view of femoral histology in plane polarized light (PPL). Letters indicate positions of higher magnification photomicrographs B–F. Anterior is toward the top. Scale bar = 3 mm. (**B**) PPL image of a thick, highly vascularized cortex (left) that surrounds an open medullary space (right). Sparse perimedullar erosion rooms indicate that bone remodeling occurred in PVSJ 606. Primary radial vascularity with occasional longitudinal and circular anastomoses in an FLB context characterizes most of the cortex. Scale bar = 500 microns. (**C**) XPL image highlighting the onset of secondary remodeling in the deeper regions of the cortex. Two forms of secondary remodeling are present: an Internal Fundamental System (IFS), indicated by layers of centripetally deposited, avascular lamellar fibered bone (LFB) lining the open medullary cavity (yellow rectangle); and erosion rooms (white rectangle). Scale bar = 250 microns. (**D**) XPL image in the deep cortex, where a single generation of sparse secondary osteons is present (yellow rectangles). Even in these zones, primary tissue persists. Scale bar = 250 microns. (**E**) XPL with lambda compensator image documenting transition from middle (top of image) to external cortex (bottom of image). The densely vascularized FLB mid-cortex exhibits a sharp transition to much more poorly vascularized, cyclical deposits of LFB in the external cortex. In the mid-cortex, WFB is indicated by pinker regions, while more organized, LFB surrounding primary osteons (turquoise and orange). Vasculature in the mid-cortex include abundant longitudinal primary osteons with some circular and radial anastomoses. The external cortex exhibits more slowly deposited, mostly avascular LFB (orange). Scale bar = 500 microns. (**F**) XPL image provides a closer look at the periosteal surface of *Proterochampsa*. At the periosteal surface, at least six cycles of mostly avascular LFB punctuated by LAGs form an EFS. The EFS signals the attenuation of appositional growth linked with the attainment of skeletal maturity. Scale bar = 500 microns.

**Fig 11:**
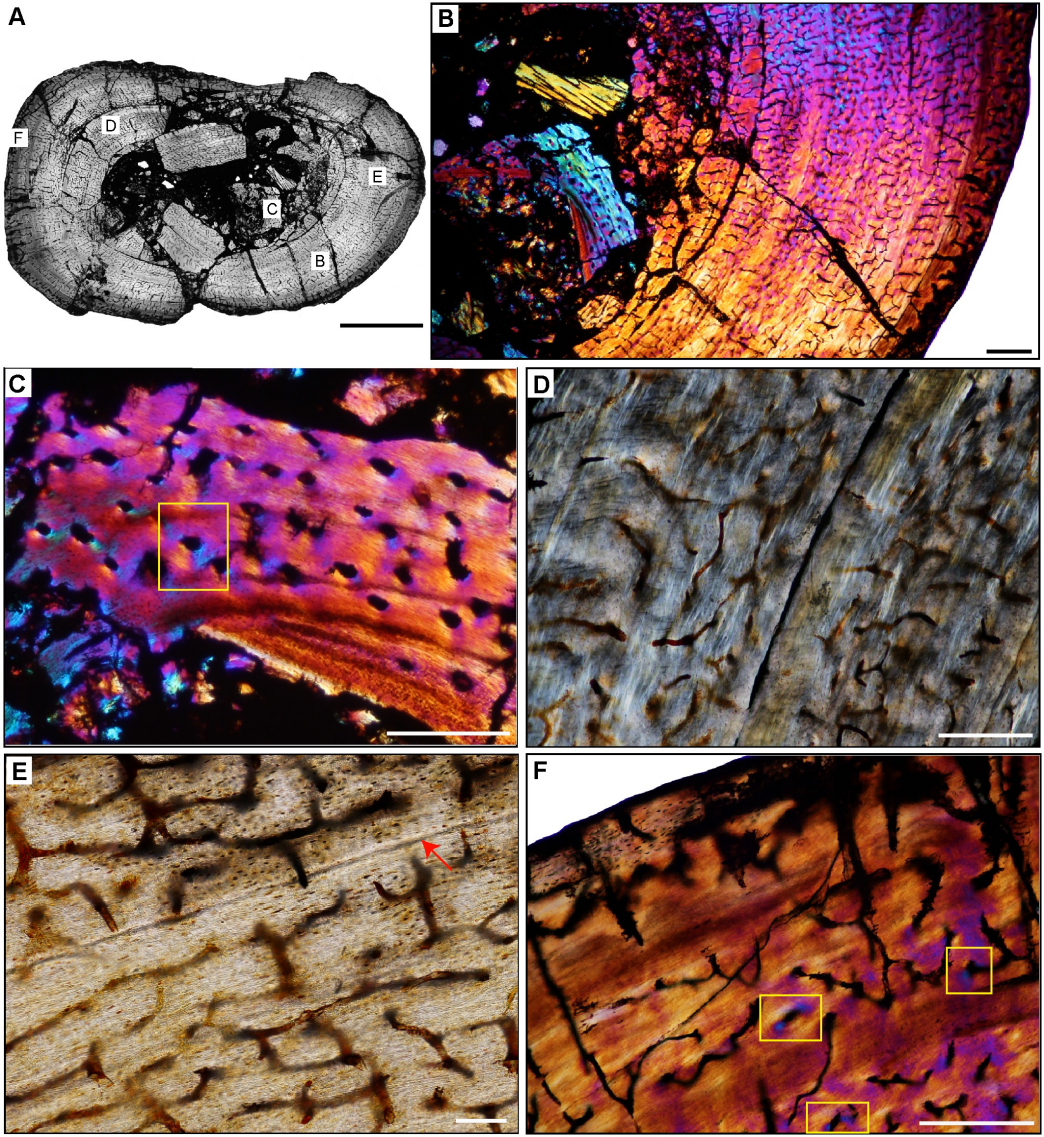
Femoral Histology of *Pseudochampsa ischigualastensis* PVSJ 567. (**A**) General view of femoral histology in PPL. Letters indicate positions of higher magnification photomicrographs B–F. Anterior is toward the top. Scale bar = 3 mm. (**B**) XPL with lambda compensator image of the open medullary cavity surrounded by a cortex dominated by cyclically deposited PFB and/or LFB. In spite of the relatively slow depositional rate indicated by bone mineral organization in *Pseudochampsa*, the femur maintains abundant but simple vascular canals and osteocyte lacunae. Scale bar = 500 microns. (**C**) XPL with lambda compensator image focused on the perimedullar area of the femur. Multiple cross-cutting lamellae form an IFS and indicate medullary drift. Other evidence of secondary remodeling, including erosion rooms and/or secondary osteons, is absent, even in the deep cortex. A few vascular canals in the deepest cortex highlight patchy formation of longitudinal primary osteons (yellow rectangle). Scale bar = 300 microns. (**D**) XPL image in the mid-cortex records the highly organized LFB that dominates the cortex. In these more external regions of the cortex vascular canals are irregular, reduced in their diameters, and lack the circumferential LFB that would indicate the presence of primary osteons. These are instead simple vascular canals directly embedded in a slow growing LFB matrix. Scale bar = 250 microns. (**E**) PPL image across a LAG (red arrow) capturing the end of one growth cycle (at the bottom) and the beginning of another (at the top). Note the difference in osteocyte lacuna abundance surrounding the LAG, and the lack of clear organization of osteocyte lacunae within LFB surrounding primary osteons. Scale bar = 100 microns. (**F**) XPL image with lambda compensator at the periosteal border of the femur. The external cortex records ongoing deposition of primary LFB with highly organized osteocyte lacunae in the context of avascularity/low vascularity at the time of death. The specimen lacks an EFS. In this view we can observe sporadic occurrence of sparse longitudinal primary osteons (yellow rectangles), but most vascular canals are simple and irregular. Scale bar = 300 microns.

**Fig 12:**
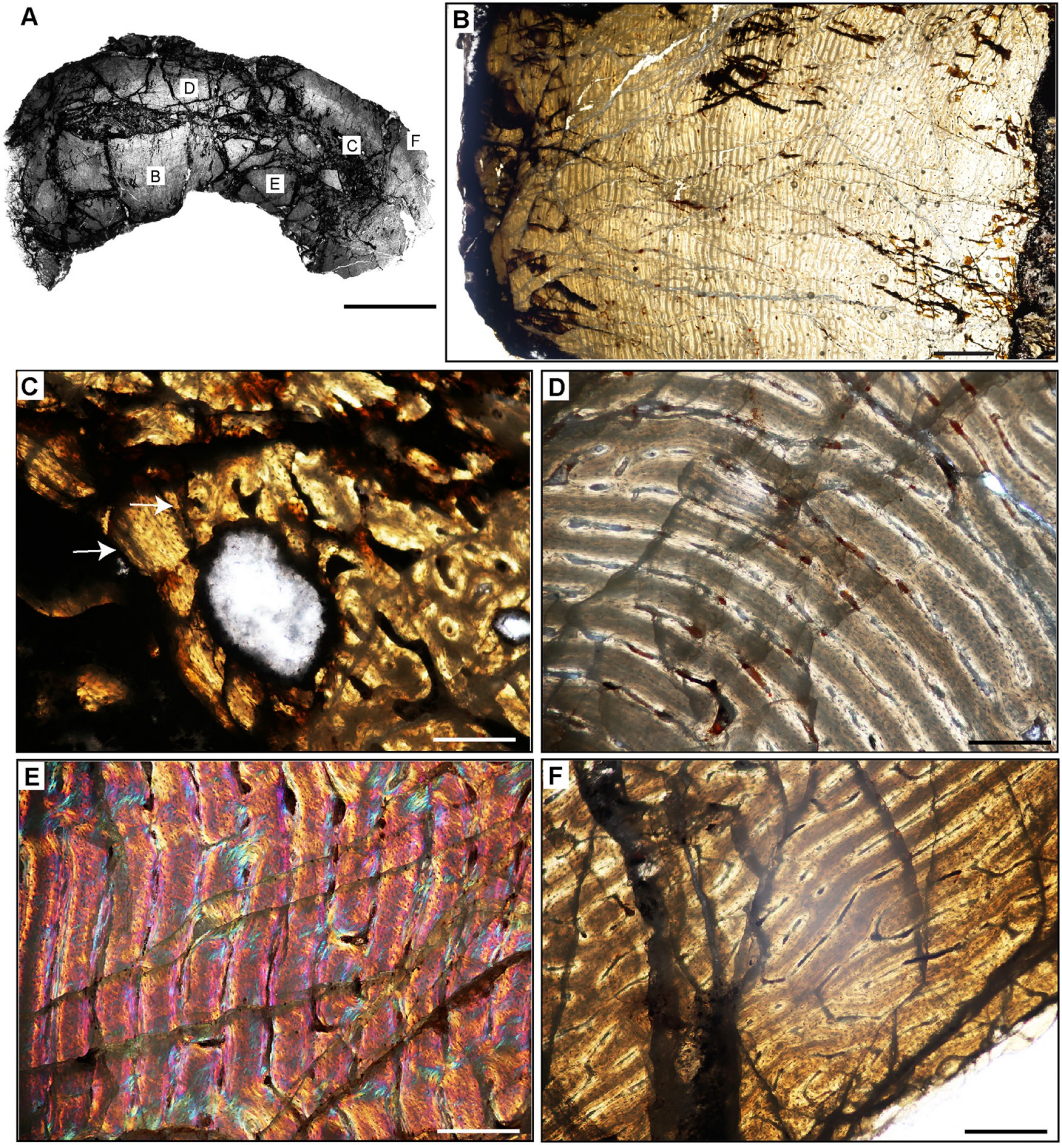
Femoral Histology of *Sillosuchus longicervix* PVSJ 085. (**A**) General view of femoral histology in PPL. Letters indicate positions of higher magnification photomicrographs B–F. Anterior is toward the top. Scale bar = 10 mm. (**B**) PPL image of a thick, highly vascularized cortex (left) that surrounds, what appears to be an open medullary cavity (left). Scale bar = 1 mm. (**C**) PPL image of the perimedullar deep cortex. A narrow band of LFB that cross cuts primary bone tissue indicates the formation of an IFS. White arrows pinpoint the borders of the IFS. Sparse evidence of perimedullar erosion rooms also indicate that bone remodeling occurred in PVSJ 085. There is no evidence for centripetal infill of erosional rooms, and secondary osteons are absent. Scale bar = 250 microns. (**D**) XPL image of mid-cortex highlighting the dominant pattern of primary bone vascularity and bone mineral organization in *Sillosuchus*. Primary osteons (white) are embedded in woven fibered bone (gray) to form a traditional fibrolamellar complex. Circular and longitudinal primary osteons dominate the cortex and form a laminar vascular pattern. Scale bar = 250 microns. (**E**) XPL with lambda compensator image of the more superficial outer cortex on the posterior side of the element highlights ongoing deposition of a well-developed laminar vascular network in a fibrolamellar context. Pink regions indicate the woven bone component of the fibrolamellar complex. Turquoise and orange regions highlight the more organized lamellar bone components surrounding primary osteons. Scale bar = 250 microns. (**F**) PPL image of the external cortex in *Sillosuchus*. In the outermost cortex, primary vasculature persists in an anastomosing network, though a transition to somewhat smaller primary canals exists. At the periosteal surface (toward the bottom right) an EFS is absent, indicating that *Sillosuchus* was still actively undergoing appositional skeletal growth when it died. Scale bar = 250 microns.

**Fig 13:**
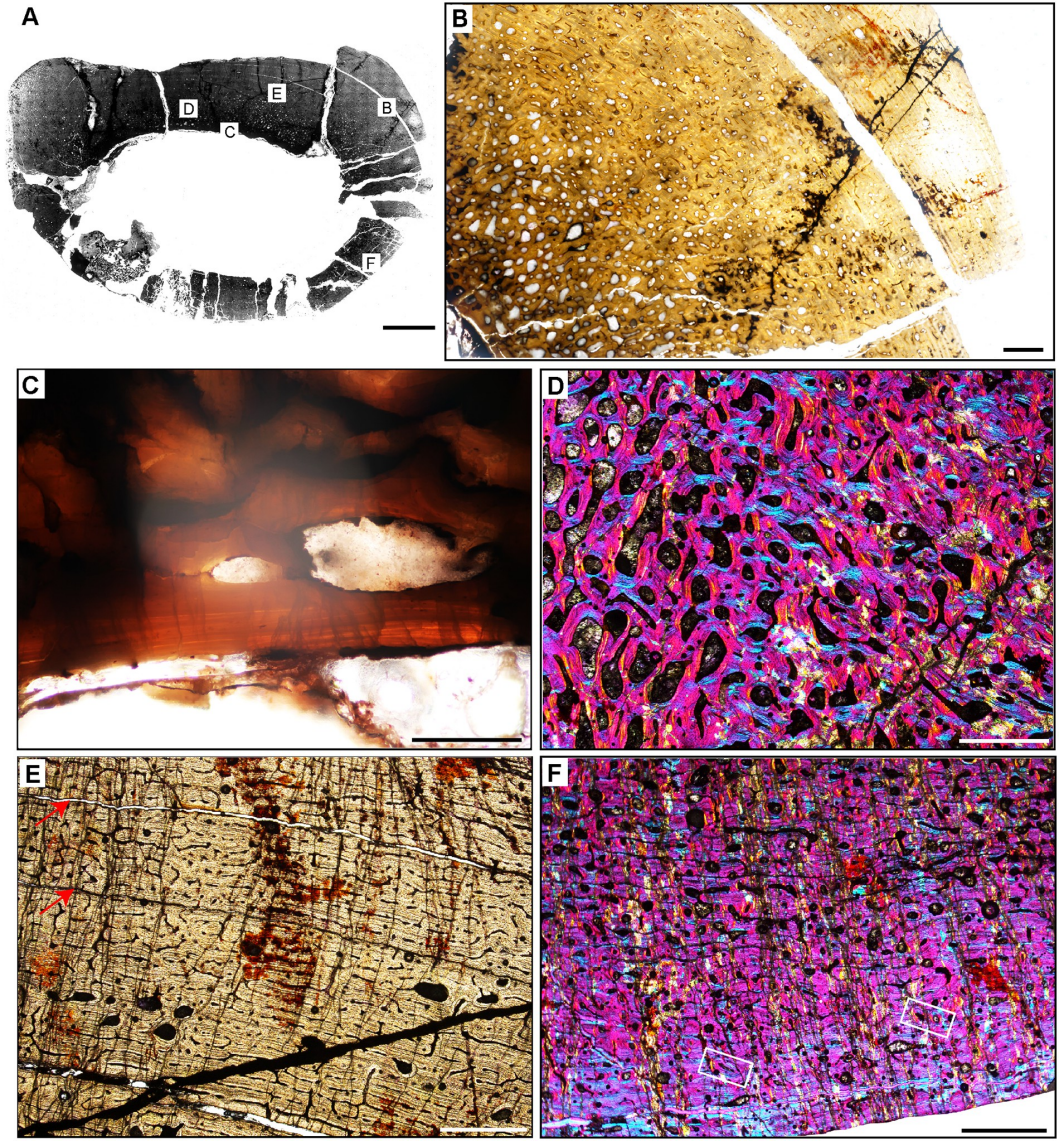
Femoral Histology of *Saurosuchus galilei* PVSJ 047 (Figs 5C, 5D and 12). (**A**) General view of femoral histology in PPL. Letters indicate positions of higher magnification photomicrographs B–F. Anterior is toward the top. Scale bar = 10 mm. (**B**) PPL image of the highly vascularized remodeled cortex and open medullary cavity in *Saurosuchus*. The medullary space is at the bottom left; the periosteal surface is at the upper right. The crack visible mid-frame follows a LAG. Even at this low magnification intensive remodeling that extends well into the mid-cortex is visible. Scale bar = 1 mm. (**C**) PPL view of the perimedullar deep cortex. An IFS overprints two erosion rooms and indicates medullary drift and remodeling in *Saurosuchus*. (**D**) XPL with lambda compensator image of the deep cortex highlighting the intensity of secondary remodeling. A single generation of secondary remodeling overprints some areas of primary bone in the deep cortex. Scale bar = 1mm. (**E**) PPL image of the *Saurosuchus* mid-cortex. Primary bone tissue is fibrolamellar with a dense laminar vascular network with occasional radial anastomoses. At least six mid-cortical LAG punctuate growth in this specimen; two are indicated here by red arrows. A third deeper LAG traceable in other regions of the cross section is aligned with the crack at the bottom of the frame. Note the erosion rooms and occasional secondary osteons extending into the mid-cortex. Scale bar = 1 mm. (**F**) XPL with lambda compensator image, outer cortex and periosteal surface. Laminar primary osteons in an FLB context grade into a more organized bone tissue dominated by PFB and patches of LFB with fewer smaller longitudinal primary osteons as we approach the periosteal margin of the bone (bottom). Note that even here in the external cortex a few sparsely distributed secondary osteons exist (white rectangles) and indicate the extent of sub-periosteal cortical bone remodeling. Scale bar = 1 mm.

**Fig 14:**
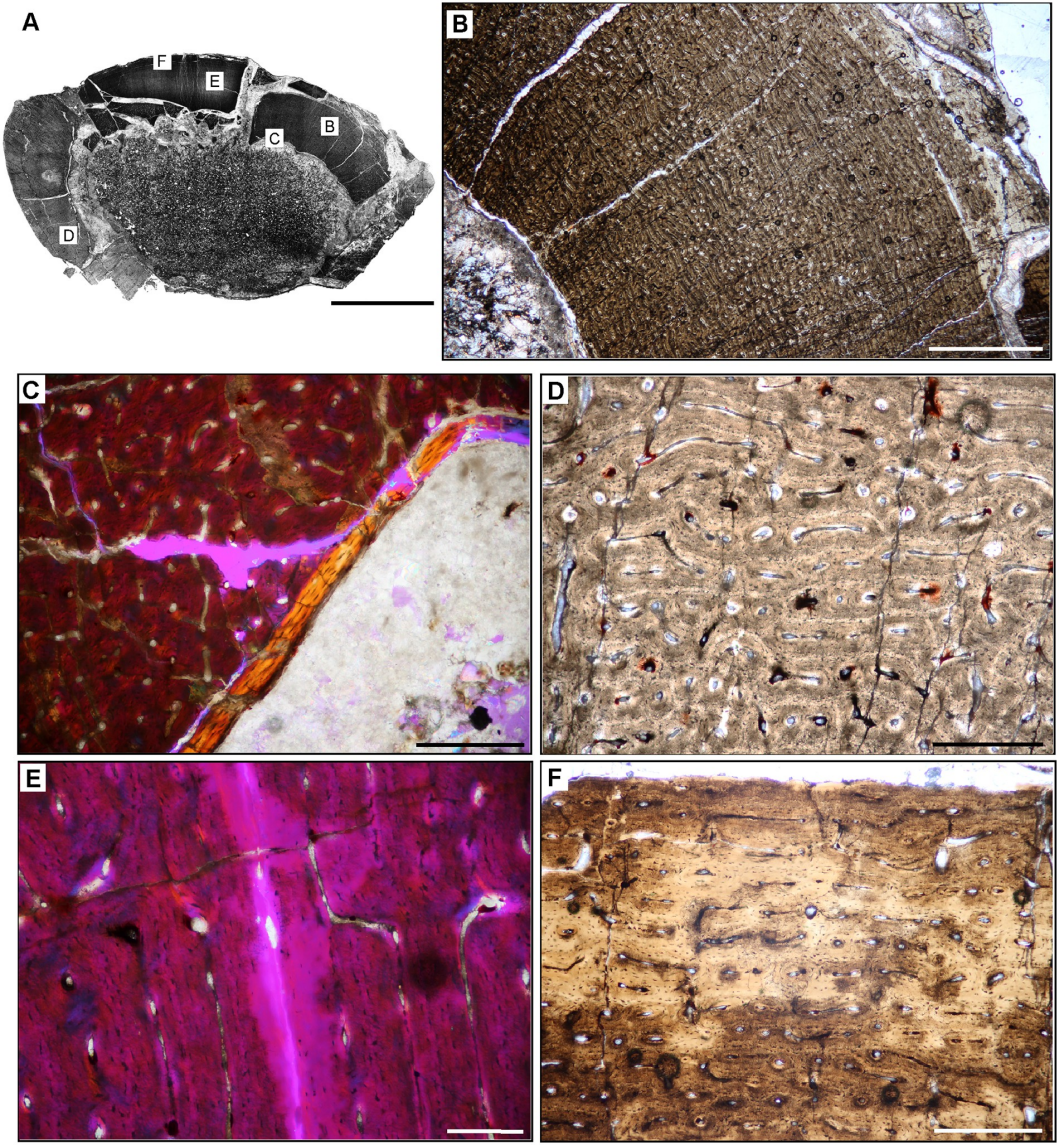
Femoral Histology of *Trialestes romeri* PVSJ 368. (**A**) General view of femoral histology in PPL. Letters indicate positions of higher magnification photomicrographs B–F. Anterior is toward the top. Scale bar = 1 cm. (**B**) PPL image of cortical bone tissue. Medullary cavity toward bottom left, periosteal surface at top right. Densely interweaving vascular networks characterize *Trialestes* femoral histology. Scale bar = 300 microns. (**C**) XPL with lambda compensator image of the deepest cortex. Band of yellow-orange highlights the IFS, a signature of medullary drift and deep cortical remodeling, which cross-cuts densely vascularized primary fibrolamellar bone tissue. Note the absence of other forms of remodeling, including erosion rooms and/or secondary osteons. Scale bar = 300 microns. (**D**) PPL image of mid-cortex records densely vascularized laminar primary fibrolamellar bone in which circular and longitudinal primary osteons interweave. Scale bar = 300 microns. (**E**) XPL with lambda compensator image of mid cortex documenting a patch of the superficial primary cortex recording a transition to LFB and/or PFB with a significant, but temporary reduction in vasculature. At least one area of high birefringence resembles a LAG, but it cannot be traced circumferentially around the cross-section, and includes small longitudinal simple vascular canals within. The thickness of adjacent laminae indicate that this birefringence may simply be a circular primary osteon interweaving with a few longitudinal primary osteons within a typical laminar vascular network. Scale bar = 100 microns. (**F**) PPL image of the external cortex of *Trialestes*. Deposition of laminar FLB persists, though longitudinal primary osteons become more common than their anastomosing circular primary osteons. At the periosteal surface (top) a deceleration of bone apposition is recorded by a shift toward more organized osteocyte lacunae in parallel rows within an PFB/LFB context, and vasculature becomes dominated by unidirectional longitudinal primary osteons. The EFS is absent, indicating ongoing, but slower growth at this later phase of *Trialestes* ontogeny. Scale bar = 300 microns.

**Fig 15:**
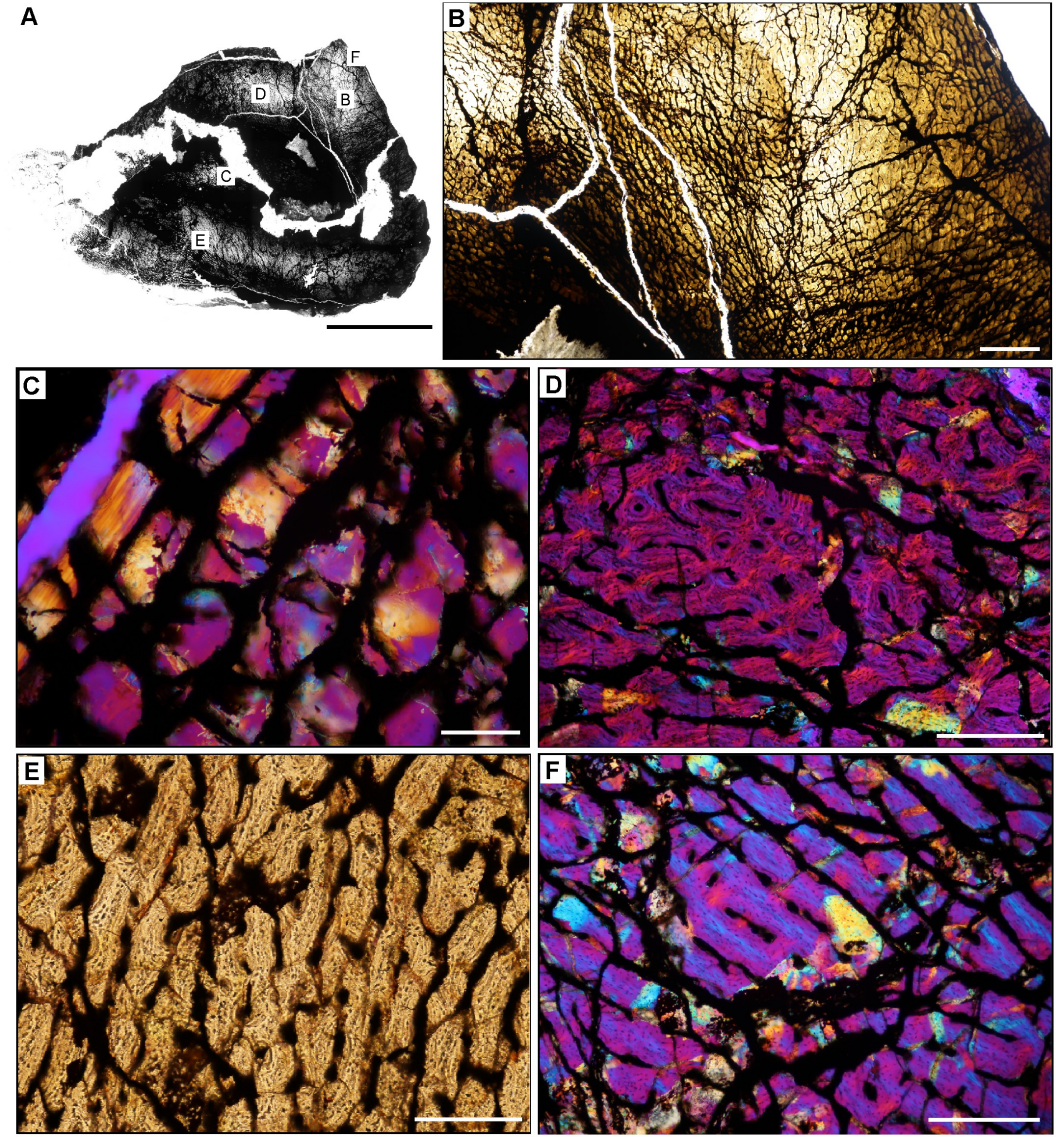
Femoral histology of *Sanjuansaurus gordoilloi* PVSJ 605. (**A**) General view of femoral histology in PPL. Letters indicate positions of higher magnification photomicrographs B–F. Anterior is toward the top. Scale bar = 10 mm. (**B**) PPL image highlighting brecciated and diagenetically altered preservation of *Sanjuansaurus*. In spite of relatively poor preservation, primary histological features can still be observed. *Sanjuansaurus* exhibits an open medullary cavity and a densely vascularized primary bone cortex. Scale bar = 1 mm. (**C**) XPL image with lambda compensator highlights thin avascular endosteal lamellae surrounding the open medullary cavity (top left) and forming an IFS. The deep cortex lacks other evidence of secondary remodeling. Scale bar = 100 microns. (**D**) XPL with lambda compensator image of mid-cortex documents fibrolamellar bone tissue that is highly vascularized by reticular primary osteons. Scale bar = 300 microns. (**E**) PPL image of more superficial cortex indicates the consistency of laminar and/reticular primary osteons in a fibrolamellar context that dominate the appositional growth pattern of *Sanjuansaurus*. The cortex is completely devoid of secondary osteons, erosion rooms, annuli, and LAG. Scale bar = 300 microns. (**F**) XPL with lambda compensator image of the external cortex. Periosteal surface is toward top right corner of image. Blue areas highlight more organization of the bone matrix, with osteocyte lacunae organized into parallel lines. In the outer regions of the cortex the woven bone scaffold transitions to a parallel-fibered organization. Primary osteons (here highlighted in pink and orange) are more unidirectional, and usually longitudinal. This femur lacks an EFS. Scale bar = 300 microns.

**Fig 16:**
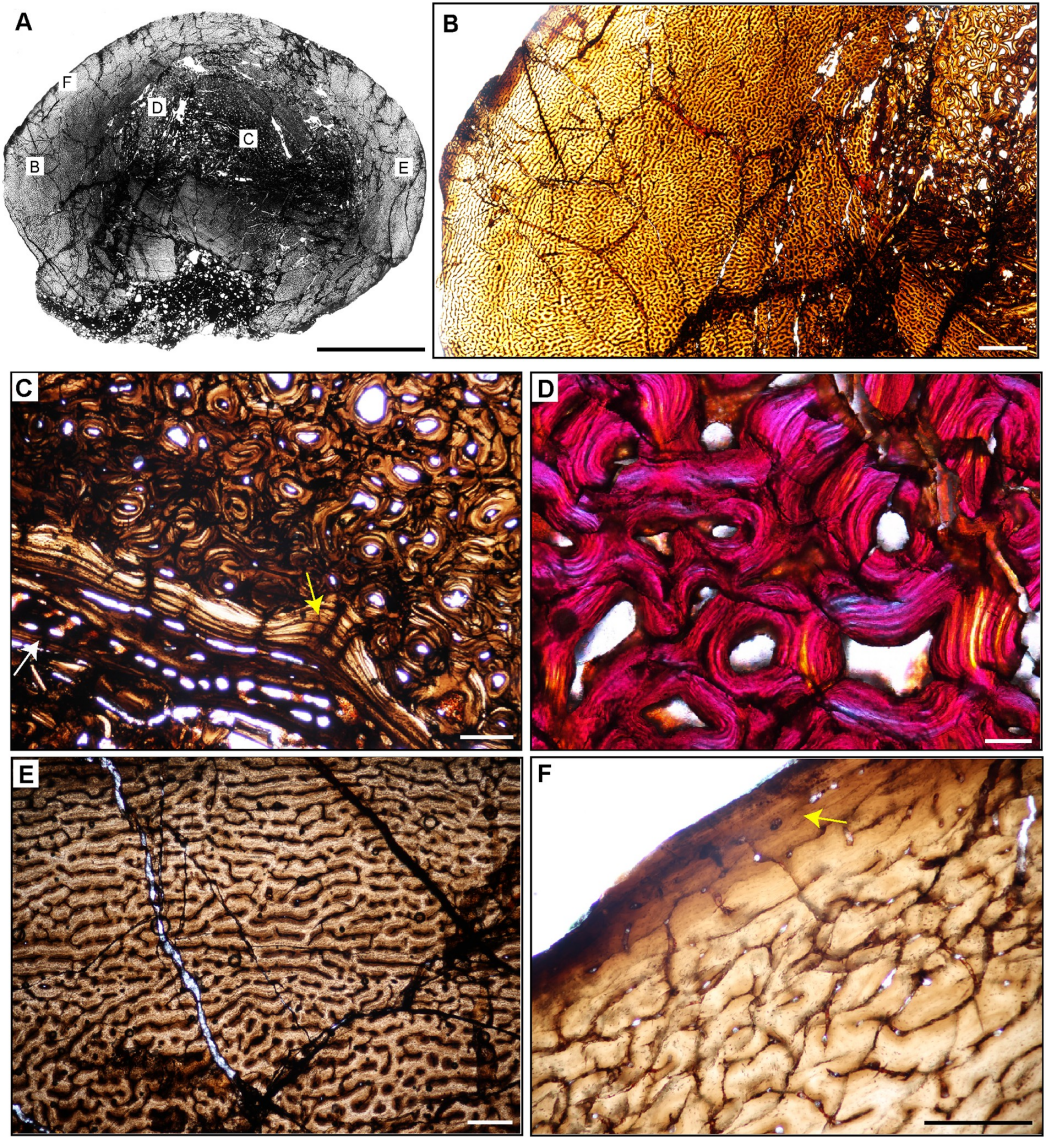
Femoral histology of *Herrerasaurus ischigualastensis* PVSJ 614. (**A**) General view of femoral histology in PPL. Letters indicate positions of higher magnification photomicrographs B–F. Anterior is toward the top. Scale bar = 10 mm. (**B**) PPL image from medullary cavity (right) to periosteal surface (left). A densely vascularized cortex surrounds an open medullary cavity lined by an IFS. Scale bar = 1 mm. (**C**) PPL image of the perimedullar deep cortex highlighting the IFS lining the medullary cavity (yellow arrow) with a zone of wide open vascular canals that are interbedded between another, and a more superficial layer of highly organized lamellae (white arrow). The bony tissue visible deep to the IFS lining in the bottom left of this image are pieces of cortex displaced by breakage into the open medullary cavity. Scale bar = 300 microns. (**D**) XPL with lambda compensator image highlighting deep cortical dense Haversian bone tissue formed through multiple bouts of bone remodeling that completely obliterates original primary bone. Scale bar = 100 microns. (**E**) PPL image of the middle cortex documents continuous fibrolamellar bone tissue with densely interweaving laminar and reticular primary osteonal networks. There are no LAG or annuli observed in the cortex of *Herrerasaurus*. Scale bar = 300 microns. (**F**) PPL image of the external cortex. Reticular fibrolamellar bone tissue dominates the last phase of growth in *Herrerasaurus*, and grades in some areas of the periosteal surface to patchy parallel-fibered bone tissue with reduced vascularity (yellow arrow). This change is circumferentially inconsistent, with other periosteal surfaces still recording ongoing active appositional growth. No EFS is observed, indicating that *Herrerasaurus* was still growing at the time of death. Scale bar = 300 microns.

**Fig 17:**
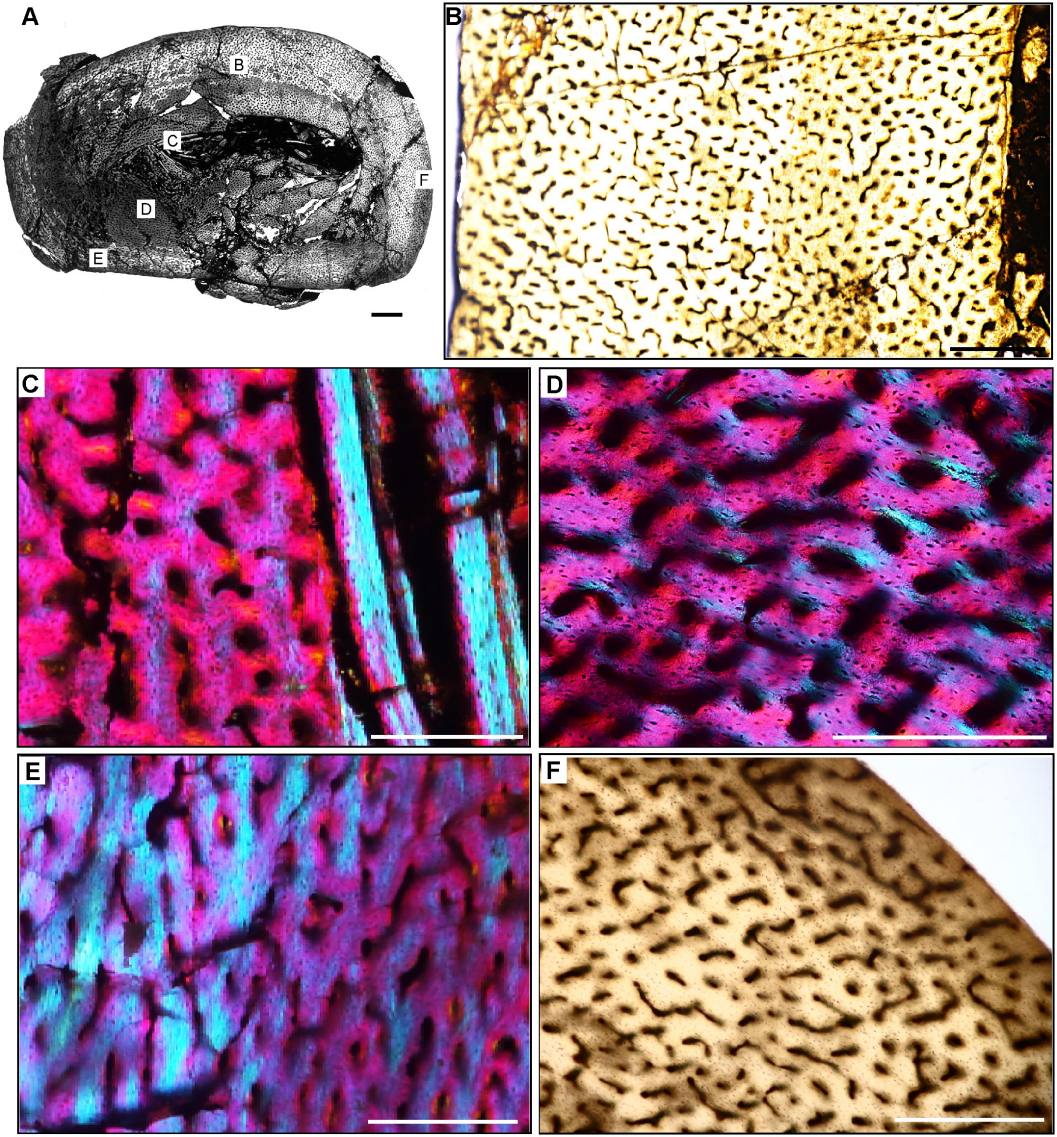
Femoral Histology of *Eodromaeus* murphi PVSJ 561. (**A**) General view of femoral histology in PPL. Letters indicate positions of higher magnification photomicrographs B–F. Anterior is toward the top. Scale bar = 1 mm. (**B**) PPL image spanning the medullary cavity (right) to the periosteal surface (left). A densely vascularized fibrolamellar cortex devoid of secondary osteons and/or erosion rooms surrounds an open medullary cavity. Scale bar = 500 microns. (**C**) XPL with lambda compensator image details lamellar fibered layers of endosteally derived bone that form an IFS lining the medullary cavity (turquoise at right). Primary tissue in the deep cortex overprinted by the IFS is reticular fibrolamellar bone, seen here in pink with orange and turquoise illustrating lamellar bone organization surrounding primary vascular canals. Scale bar = 250 microns. (**D**) XPL with lambda compensator image drawn from mid-cortex illustrates osteonal organization of lamellar fibered tissue around primary vascular canals within a woven bone scaffold. Note the reticular organization of primary vascular canals and the rounded and numerous osteocyte lacunae. Scale bar = 250 microns. (**E**) XPL with lambda compensator image near the periosteal surface highlights primary bone organizational change in later ontogeny. The middle cortex (toward the right) records a traditional fibrolamellar complex with reticular primary osteons indicative of relatively elevated growth rates. Closer to the periosteal surface (toward the left) the scaffold of apposition transitions to parallel-fibered bone mineral, indicated by the turquoise color in this micrograph. Scale bar = 250 microns. (**F**) PPL image of the external cortex highlighting an area of ongoing but slowed growth at the periosteal surface. Primary reticular fibrolamellar bone persists more deeply, but at the periosteal border osteocyte lacunae become more organized in parallel layers reflecting the parallel organization of bone mineral. These changes are accompanied by a shift toward more sparse longitudinal vascular canals. Together, these changes indicate a reduction in appositional growth but not a cessation. An EFS is absent in *Eodromaeus*. Scale bar = 200 microns.

**Fig 18:**
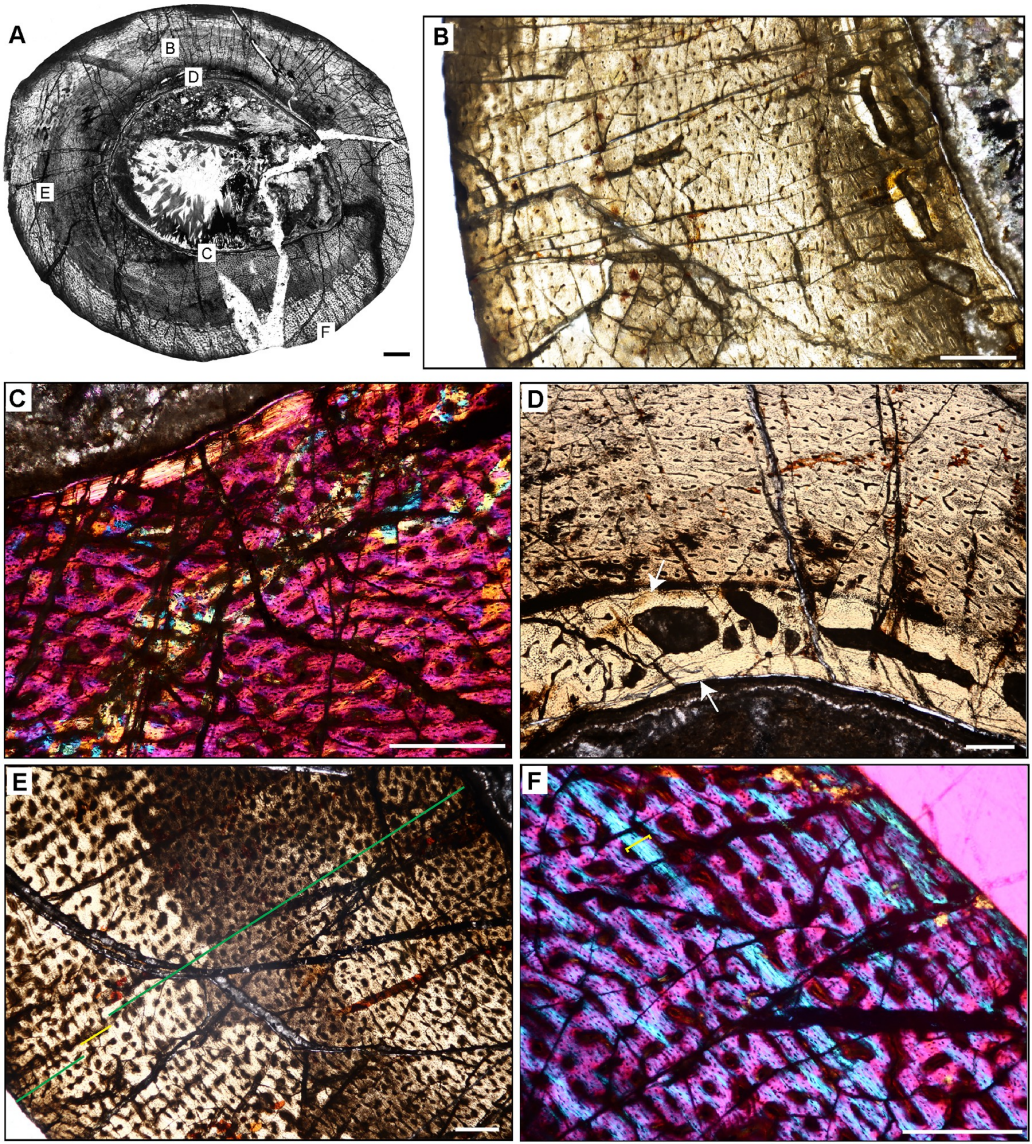
Femoral histology of *Eoraptor lunensis* PVSJ 559. (**A**) General view of femoral histology in PPL. Letters indicate positions of higher magnification photomicrographs B–F. Anterior is toward the top. Scale bar = 1 mm. (**B**) PPL image spanning the medullary cavity (right) to the periosteal surface (left). Unusually large erosional spaces are visible in the deep cortex. The cortex preserves well-vascularized fibrolamellar bone. LAG are absent. Scale bar = 500 microns. (**C**) XPL with lambda compensator image of perimedullar cortex. Orange color in the top left highlights the well-developed IFS lining the medullar cavity. The deep cortex exhibits fibrolamellar bone dominated by abundant longitudinal primary osteons. Scale bar = 300 microns. (**D**) PPL image of perimedullar erosion bays that are situated between two layers of endosteally-derived lamellae (white arrows) that have eroded primary bone tissue and reflect medullary drift. Between these thin lamellar layers large erosion rooms have eroded away compacted Haversian osteonal bone. Scale bar = 300 microns. (**E**) PPL image of the cortex *Eoraptor* documenting three observable growth intervals, in the context of continuous deposition of well-vascularized fibrolamellar bone. The deepest cortex (green line, upper right) exhibits abundant circular and longitudinal primary osteons that sometimes interweave in a laminar pattern. This segment encompasses the majority of appositional growth in *Eoraptor*. Following this burst of growth, a period of deceleration is indicated by a shift toward less primary vasculature and parallel-fibered bone tissue (yellow line). A return to faster growth later in ontogeny is indicated by more highly vascularized fibrolamellar bone (green line, lower left). In some regions of the last growth interval at the periosteal surface circular, longitudinal, and radial primary osteons interweave in a sub-plexiform pattern. Scale bar = 300 microns. (**F**) XPL with lambda compensator image of another area of the outer cortex in *Eoraptor*. This image highlights the same pattern observed in (E, yellow line) zooming in on the region of growth deceleration. Parallel-fibered bone tissue surrounds mostly longitudinal primary osteons (lower left). The bright turquoise line (yellow bracket) indicates a transition to avascular lamellar fibered bone tissue signaling a temporary but significant decrease in bone apposition. This annulus is not traceable circumferentially. More periosteally, primary osteons exist in a woven-fibered scaffold to form a traditional, highly vascularized fibrolamellar complex during the final recorded phase of growth for *Eoraptor*. The lack of an EFS indicates that this individual was still actively growing at the time of death. Scale bar = 300 microns.

**Fig 19:**
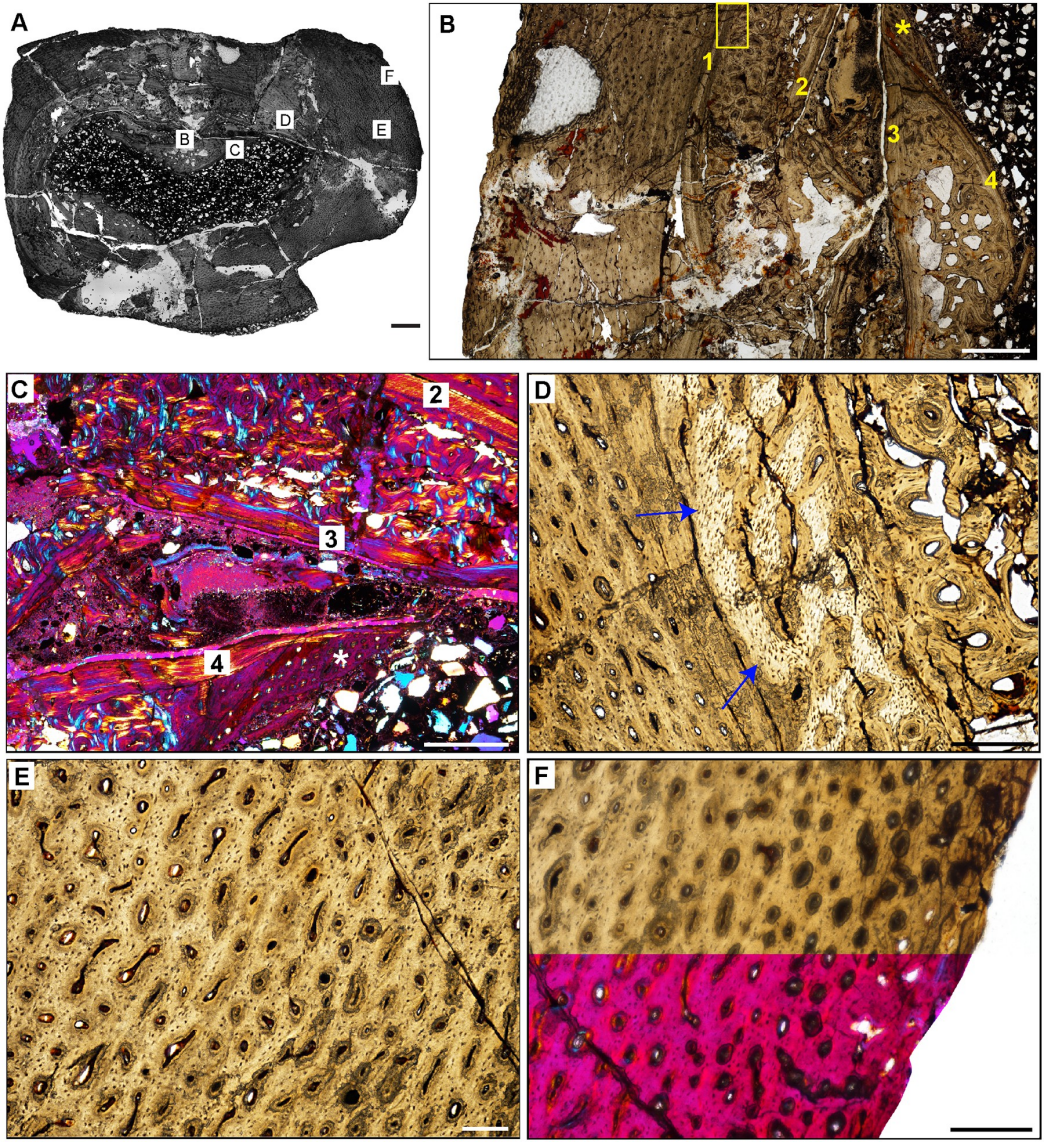
Femoral histology of *Chromogisaurus novasi* PVSJ 845. (**A**) General view of femoral histology in PPL. Letters indicate positions of higher magnification photomicrographs B–F. Anterior is toward the top. Scale bar = 1 mm. (**B**) PPL image spanning the medullary cavity (right) to the periosteal surface (left). Four cycles of bone remodeling each include centrifugal erosion, followed by centripetal deposition of endosteally-derived LFB, and continued erosion of this LFB through the formation of secondary osteons/dense Haversian bone tissue. The endosteal LFB layers for each of these cycles is indicated by the numbers, 1–4, with 1 representing the initial erosional cycle, and 4 representing the cycle that was most close to the time of death/most recent. The same numbers and the star also apply to cycles labeled in **C**. The yellow rectangle indicates the approximate position of **D**. Note the unusually large erosional spaces are visible deep to cycle 3, and in the patch of primary woven-fibered bone tissue lining the perimedullar space, indicated by the yellow star. Superficial to the most external layer of endosteal lamellae the cortex preserves well-vascularized fibrolamellar bone. LAG are present but only in association with deep cortical remodeling. Scale bar = 500 microns. (**C**) XPL with lambda compensator image of perimedullar cortex. Numbers indicate the intervals of deep cortical centripetal deposition of lamellar bone tissue following an episodic cycle of medullary expansion. Between these intervals, these secondary bone tissue deposits are also eroded, and are replaced by secondary osteons that form dense Haversian bone. This signature is especially clear between Cycles 2 and 3 in this image. Cycle 4 includes both endosteally-derived LFB, secondary osteonal bone, and longitudinally vascularized woven fibered bone that lines the medullary cavity (indicated by the white star). Scale bar = 300 microns. (**D**) PPL image of perimedullar secondary osteons and erosion rooms are situated between two layers of endosteally-derived lamellae (cycles 1 and 2 in image B). Blue arrows indicate the superficial extent of centrifugal erosion and reversal in cycle 1. Secondary osteons gradually replace earlier deposits of endosteally-derived LFB. More deeply, between cycles 1 and 2, secondary osteons have obliterated primary bone tissue. Scale bar = 500 microns. (**E**) PPL image of the cortex *Chromogisaurus* documenting the general nature of the middle cortex. Superficial to deep cortical signatures of remodeling, primary fibrolamellar bone tissue vascularized by abundant longitudinal vascular canals persists to the external cortex. In some regions of the mid-cortex circular and longitudinal primary osteons anastomose in a sub-laminar to laminar vascular arrangement. Scale bar = 250 microns. (**F**) PPL and XPL with lambda compensator composite image of another area of the outer cortex. Deposition of fibrolamellar bone tissue vascularized by longitudinal primary osteons persists in preserved regions of the external cortex. The lack of an EFS or a significant change in bone depositional pattern indicates ongoing appositional growth in *Chromogisaurus* at the time of death. Scale bar = 500 microns.

We employ the osteohistological terminology of Francillon-Vieillot et al. [1] and Buffrénil et al. [5], and we follow Nesbitt and colleagues’ phylogeny [99] supplemented with other recent analyses of the early dinosaur tree to contextualize our histological descriptions [100, 101]. We first briefly introduce each taxon, and follow with descriptions of bone microstructure for each femur, organized from noteworthy features of the medullary space and deep cortex outward to the periosteal margin (Figs 8–19). We focus on primary bone mineral organization, primary vascular orientation, presence/absence of growth marks, including annuli and/or LAGs and the EFS, and also document features of secondary bone remodeling, including trabecular formation, formation of an IFS, and/or development of secondary osteons. To streamline later discussion and avoid redundancy, we conclude our Ischigualasto-specific histological descriptions with a comparison of our results to those previously published for each taxon.

### Synapsida

#### *Exaeretodon argentinus* PVSJ 38–2002 (Figs 4A, 4B and 8)

Cynodontia was the last therapsid group to evolve, and includes mammals as extant representatives [102]. By the end of the Triassic the group was globally distributed and had diversified into a wide array of taxa varying in lifestyle, diet, and morphology [103]. Traversodontidae is a clade of herbivorous non-mammaliform cynodonts that thrived during the Middle and Late Triassic [104]. *Exaeretodon* is among the largest members of this clade [105, 106], and femur PVSJ 38–2002 is from the largest individual to have been sampled histologically to date (Table 1) [89, 105, 107]. *Exaeretodon* fossils represent ~17% of the tetrapod diversity in the Cancha de Bochas Member of the Ischigualasto Formation [20, 67, 69, 89].

*Exaeretodon* femur PVSJ 38–2002 exhibits a thick compact cortex surrounding a narrow cancellous spongiosa packed with highly remodeled, broken, bony trabeculae (Figs 4A, 4B and 8A). Within the coarse and cancellous spongiosa large erosional spaces are lined with endosteal lamellar bone indicating cycles of bone erosion and redeposition over the course of ontogeny (Fig 8B and 8C). Bone erosion extends into the perimedullar deep cortex, where a few erosion rooms are developed and sparse secondary osteons overprint some areas of primary bone (Fig 8B and 8C). FLB characterizes primary bone throughout most of the cortex. Primary vasculature within the FLB context is composed of mature, highly infilled, and widely spaced longitudinal and circular canals that interweave within a woven bone matrix (Fig 8D and 8E). The intensive lamellar infilling of these primary vascular spaces imparts a “highly organized” morphology, but close examination under cross-polarized light highlights the presence of thin woven bone components between primary osteons (Fig 8D). This pattern persists in all active growth zones in the cortex. Active appositional growth in the cortex is punctuated by two broad regions of mid-cortical growth marks (Fig 8B). The first occurs in the mid-cortex, superficial to the perimedullar region of compacted bone and bony trabeculae (Fig 8E). This first growth mark includes a narrow region of 3–4 annuli and a LAG in the context of a shift toward more highly organized PFB/LFB and reduced vascularity. This signature is traceable around the circumference of the element (Figs 4B, 4C, 8A, 8B and 8E). A circumferential ring of erosional spaces and incipient secondary osteons occurs between each of the annuli (Fig 8E). FLB deposition resumes following this sustained pause in growth, though vasculature is reduced and dominated by more longitudinal primary osteons. This more superficial zone of active growth is terminated by a second more superficial pause in bone apposition again recorded by 3–4 stacked annuli and a LAG in a LFB matrix (Fig 8A and 8B). After this second deceleration, primary bone growth resumes in a final narrow peripheral growth zone. Vasculature in this region is very sparse, and in some areas the element is avascular (Fig 8F). The periosteal border exhibits stacked LAGs and annuli in the context of avascular LFB forming an EFS that signals the cessation of growth and attainment of maximum size (Fig 8F).

The bone tissues of therapsid clades are relatively well known and generally reveal life histories characterized by highly vascularized FLB [105, 108–116]. Recently Veiga and colleagues [105] analyzed multiple appendicular elements at variable ontogenetic stages for *Exaeretodon riograndensis* from the Late Triassic of Brazil. Our observations for PVSJ 38–2002, though from an individual a bit larger than that sampled by Veiga and colleagues [105], are consistent with those of the largest femur in their study. In both of these *Exaeretodon* samples, well-vascularized primary FLB characterizes most of the ontogeny [105], though patterns of vasculature vary throughout the cortex in all sampled individuals. Multiple cyclical pauses in bone deposition are also indicated by annuli and LAG, with decreasing bone apposition between these cyclical LAG approaching the periosteal surface. A gradual shift to more highly organized LFB occurs near the periosteal surface in both specimens. In contrast with the *Exaeretodon* sample of Veiga and colleagues [105], PVSJ 38–2002 exhibits an EFS.

### Archosauromorpha

#### *Hyperodapedon sanjuanensis* PVSJ 574 (Figs 4C, 4D and 9)

Rhynchosauria is a group of non-archosauriform archosauromorphs restricted to the Triassic Period. The group includes the first large reptiles to exploit herbivory [90, 117–119]. Rhynchosaurs are among the most common members of Triassic faunas and include taxa with well-preserved ontogenetic series [88–90]. Most studies of rhynchosaur growth have focused on rib histology [17–18, 22, 39, 41], with a few analyses exploring histology among multiple elements and/or ontogenetic series [e.g., 19, 117–119]. The *Hyperodapedon* femur we sampled (PVSJ 574) is similar in size (Table 1, 21.1 cm) to that of another sampled specimen from India (24.8 cm) [19, 90]. *Hyperodapedon* fossils represent ~60% of the recovered fossils in the Cancha de Bochas Member of the Ischigualasto Formation [20, 67, 69, 90].

The femur of *Hyperodapedon* (PVSJ 574) exhibits a thick compact cortex. Antero-posterior compression and breakage makes it difficult to discern the nature of the medullary region, but it appears to be filled with broken remnants of a spongiosa composed of thin spicules of remodeled bony trabeculae (Figs 4C, 4D and 9B). There are a few very sparse but large erosional spaces in the deep cortex, with a few extending into more superficial regions of the mid-cortex (Fig 9B and 9C). All of these erosion rooms indicate the onset of limited remodeling of primary cortical bone in *Hyperodapedon*. Circumferential cementing lines and centripetal deposition hint at the onset of secondary osteon formation in some of the erosion rooms (Fig 9C), but mature secondary osteons are absent. Primary FLB with abundant interweaving, irregular, and reticular vascular networks of primary osteons dominates the cortex of PVSJ 574 (Fig 9D). Throughout the cortex osteocyte lacunae are miniscule but very dense in the woven bone components of the fibrolamellar complex (Fig 9D).

The active growth recorded by such highly vascularized FLB in the cortex of PVSJ 574 is divided into five zones demarcated by circumferential annuli and/or LAG (Figs 4C, 4D, 9A and 9B) that impart a stratified morphology to the transverse section of PVSJ 574. The most external zone is narrower than the deeper zones, though some mid-cortical zones are thinner than the most peripheral zone. From deep to superficial, subtle changes to bone microstructure also point to a relative reduction in the rate of primary bone osteogenesis within each active growth cycle in later ontogeny (Fig 9B, 9C, 9E and 9F). Variation also records changes to bone depositional pace within each cycle. Each growth cycle begins with a pulse of well-vascularized FLB bone, followed by a transition to less vascularized PFB. Within this area of PFB, annuli are more common, and many growth cycles conclude with an appositional cessation indicated by a LAG. This pattern indicates a gradual slowing of growth followed by a stop, and then a resumption of active apposition as ontogeny continues (Fig 9E). These variations are accompanied by a reduction in primary bone vascular anastomoses and osteocyte lacunae density (Fig 9E and 9F). Following each interval of growth cessation the signal reverses, as LFB/PFB grades into FLB and signals a gradual resumption of faster deposition within the next active growth zone (Fig 9E and 9F). Sometimes the resumption of FLB deposition is abrupt (e.g., Fig 9E), and at other times it can occur more gradually (Fig 9F). The zones decrease in width from deep to superficial, with the most external zones preserving the narrowest zones of primary bone deposition (Fig 9A, 9B and 9F). In the most external portions of PVSJ 574, primary vascular canals are either longitudinal, or form a relatively sparse network of longitudinal and circular vascular canals in a sub-laminar pattern (Fig 9F). PVSJ 574 lacks an EFS, though the external cortical bone documents a transition in vascular density and more closely spaced LAG (Fig 9F). This may indicate growth attenuation as adult size is approached, or may simply represent the “reset” of appositional growth following a cessation as observed in the deeper regions of earlier ontogeny cycles in the deeper cortex. Either way, our data suggest that *Hyperodapedon* was undergoing a period of significantly reduced appositional growth when it died.

Rhynchosaur bone histology has been described for species of *Hyperodapedon* from India [19] and Brazil [118]. A few isolated and indeterminate rhynchosaur elements from the Ischigualasto Formation that likely can be attributed to *Hyperodapedon* have also been briefly described [18, 39]. Though the sampled skeletal elements, ontogenetic stage, and the locations of thin-section vary amongst these samples, a general growth pattern emerges for *Hyperodapedon* that is consistent with data from our sampled femur. The majority of bone apposition in *Hyperodapadon* is characterized by early deposition of FLB with a significant woven-fibered component and abundant primary vascularization. These FLB tissues occur within cycles that are clearly demarcated by LAG. Cycles between LAG are broadest deeper in the cortex, narrowing toward the periosteal margin. Near the outer circumference of the sampled elements primary bone deposition generally transitions to PFB and/or LFB dominated by sparse longitudinal primary vascular canals, and no EFS [18, 39]. Other sampled rhynchosaur taxa also generally exhibit more well-developed signatures of secondary remodeling, including both endosteal lamellae forming an IFS and deep cortical secondary osteons [18, 118–119]. This general pattern also occurs in the Brazilian rhynchosaur *Teyumbaita sulcognathus* [118]. In contrast, the bone tissue of *Stenaulorhynchus stockleyi* from Tanzania preserves moderately vascularized PFB grading externally to LFB punctuated by regular growth marks, with deep cortical erosion rooms and a few secondary osteons, culminating in an EFS [119].

### Archosauriformes—Proterochampsia [non-archosaurian archosauriformes)

Proterochampsia are medium-sized quadrupedal carnivores endemic to the Triassic of South America [91–92, 120, 121]. Their superficial resemblance to extant crocodilians prompted the notion that they were likely semi-aquatic [91, 120–123], but bone histological data drawn from a diversity of proterochampsians from the Chañares Formation of Argentina suggest that at least some may have led more terrestrial lifestyles [121, 124, 125]. We sampled similarly sized femora from two proterochampsian genera (Table 1, ~18 cm long), both from individuals larger than other previously sampled proterochampsians [92]. Both sampled taxa represent less than ~1% of tetrapod diversity in the Cancha de Bochas Member of the Ischigualasto Formation: *Proterochamspa barrionuevoi* and *Pseudochampsa ischigualastensis* [20, 67, 69, 91, 92].

#### *Proterochampsa barrionuevoi* PVSJ 606 (Figs 4E, 4F and 10)

The femur of *Proterochampsa* (PVSJ 606) exhibits a well-defined open medullary cavity devoid of trabecular bone but lined in some regions by endosteal lamellae forming an IFS (Figs 4E, 4F and 10A–10C) [91]. These lamellae crosscut primary bone deposits in the deepest regions of the cortex, and indicate medullary drift driven by cycles of perimedullar resorption and redeposition (Fig 10C). The cortex is dominated by primary bone, but in the posterolateral regions of the deep cortex a few very sparse erosion rooms and a handful of secondary osteons indicate limited remodeling of primary cortical bone (Fig 10B–10D). Even in these areas primary bone tissue persists throughout the cortex, allowing a full view of histological patterns during ontogeny. Diagenetic alteration makes it somewhat difficult to discern the nature of primary bone mineral organization from deeper to more superficial regions in some areas of the cortex, but circumferential organization of osteocyte lacunae around primary vascular canals are distinctive from the surrounding bone matrix and point to a typical FLB organization (Fig 10D and 10E). Osteocyte lacunae in the woven bone regions of the fibrolamellar complex of PVSJ 606 are generally disorganized, but remain relatively sparse and small (Fig 10D). Radial primary osteons dominate the vascular pattern throughout the cortex, but abundant longitudinal and occasional circular anastomoses also occur (Fig 10D–10F). Mid-cortical growth marks are completely absent in PVSJ 606 (Figs 4E, 4F, 10A and 10B). The element records a sharp change in primary bone depositional pattern near the periosteal surface, where primary FLB transitions to highly organized LFB and circumferential stacked LAG (Fig 10E and 10F). These features are consistent with an EFS. This stark transition could exist because the FLB was first eroded, and the EFS then deposited on an erosional surface. If this were the case, we would expect to observe an erosional line that results in truncated primary osteons, and/or woven tissue fabric at the point where the EFS begins. These features are not observed in our sample (Fig 10A, 10E and 10F). Instead, primary bone histology in *Proterochampsa* indicates a continuously, rapid early ontogeny bone growth pattern culminating in a rapid late ontogeny shift to slower bone depositional rates approaching maximum size.

Our data for *Proterochampsa* generally align with those of previous studies of closely related taxa, with a few notable exceptions. Other sampled taxa are also characterized by highly vascularized FLB early in ontogeny that transitioned to LFB over life history [121, 124, 125]. Primary vascular patterns in other sampled femora exhibit mostly longitudinal primary osteons with few anastomoses [121]. Secondary osteons are either rare, as is the case in *Proterochampsa*, or completely absent in all other sampled taxa [121]. Other taxa share the presence of the IFS with *Proterochampsa* [121]. In most of these other sampled taxa primary appositional growth is punctuated by mid-cortical growth marks or by mid-cortical transition to PFB. A few other specimens share the transition from highly vascularized FLB to more organized LFB at the periosteal border, but in no other proterochampsian so far described is this transition as stark as it is in *Proterochampsa* [18, 124]. In contrast with previously studied proterochampsians, *Proterochampsa* PVSJ 606 records the development of an EFS.

#### *Pseudochampsa ischigualastensis* PVSJ 567 (Figs 4G, 4H and 11)

The femur of *Pseudochampsa* (PVSJ 567) exhibits an open medullary cavity (Figs 4G, 4H, 11A and 11B) [91, 92]. Though the deep cortex is broken, an endosteally derived lamellar IFS that truncates primary bone in some areas of the deep cortex can be identified (Fig 11B and 11C). Cortical erosion rooms and secondary osteons are completely absent from this specimen. Close examination indicates that, with the exception of a few patchy deposits in the deepest cortex (Fig 11C), most of primary cortex is dominated by deposits of PFB and LFB (Fig 11A–11D) organized into at least six distinctive zones, most of which are defined by LAG, but some are characterized instead by a zone of avascularity (e.g., Fig 11B). In spite of the highly organized nature of bone mineral and osteocyte lacunae in PVSJ 567, the element is highly vascularized with a dense network of small, irregular, reticular, circular, and radial canals interweaving through the cortex (Fig 11A–11C). For the most part these vascular canals are embedded directly within the primary bone, and lack a surrounding border of centripetally deposited osteonal tissue (Fig 11D–11F). As such, they cannot be diagnosed as primary osteons, and are instead best characterized as simple vascular canals. Exceptions can be found in a few places in the cortex where some osteonal organization exists around longitudinal primary vascular canals (Fig 11C and 11F). Vascular canals are also more abundant and interweaving deeper in the cortex, and become more simply organized, sporadic, and smaller in more superficial active growth zones (Fig 11D). Growth zone width decreases from the deep cortex toward the periosteal surface. Within these zones, a transition occurs from more highly vascularized PFB at the beginning of each to avascular LFB deep to the deposition of at least one LAG at the superficial margin of each zone. This pattern repeats itself in each cyclical growth zone. The most external zone exhibits circumferential layers of LFB with very tiny densely organized osteocyte lacunae. Primary vasculature persists even at the periosteal border where longitudinal canals dominate, but occasional anastomosis with radial and/or reticular canals persists, as observed in deeper cycles. PVSJ 567 lacks an EFS, and was still actively growing, albeit slowly, when death occurred (Fig 11F).

The more highly organized PFB and LFB tissues that characterize *Pseudochampsa* femoral sample PVSJ 567 throughout ontogeny starkly contrast with the highly vascularized FLB of other sampled proterochampsians, including *Proterochampsa* [18, 92, 121, 125, 126]. Instead of primary osteons embedded in a woven bone matrix, vascular canals in PVSJ 606 are generally simple and lack clear evidence of centripetally deposited osteonal bone. These simple vascular canals anastomose, but in the context of PFB and/or LFB. That said, the preponderance of radially-oriented vascularity is similar to what we observed in *Proterochampsa* (PVSJ 606). In sharp contrast with the continuous (or nearly continuous) ontogenetic growth strategy of other protochampsian samples (especially *Proterochampsa)*, tissue histology in *Pseudochampsa* indicates that growth in this taxon was punctuated by regular cessations [18, 124], and this points to a distinctive life history for this taxon (or at least this individual) relative to its close relatives.

### Archosauria—Pseudosuchia

Pseudosuchia was one of the most diverse groups of terrestrial vertebrates in Middle and Late Triassic ecosystems [127, 128]. Members of the group include quadrupedal and facultatively bipedal forms, semiaquatic and terrestrial taxa, and organisms with dietary preferences ranging from carnivory to omnivory and piscivory [127, 129–131]. Representative members of this diverse group have been the focus of previous histological inquiry [*Postosuchus*, 17; *Effigia*, 132; *Batrachotomus*, 133; *Decruriasuchus*, *Prestosuchus*, 134–135]. Here we provide the first histological insights for two pseudosuchians that represent the largest terrestrial carnivores in the Ischigualasto Formation: the poposaurid *Sillosuchus longicervix* [93] and the loricatan *Saurosuchus galilei* [94–96]. Our femoral samples for each of these taxa come from similarly sized large individuals (Table 1, femur length ≥ 46 cm long). *Sillosuchus* shares some anatomical characters with taxa like *Effigia*, *Shuvosaurus*, and *Poposaurus*, which indicate a general, gracile, bipedal body plan convergent with later evolving ornithomimid dinosaurs [136, 137]. The cervical vertebrae in *Sillosuchus* exhibit pleurocoels that are similar to those observed in later saurischian dinosaurs, and may indicate the presence of pulmonary air sacs [132, 136]. *Sillosuchus* represents only about 0.9% of Cancha de Bochas Member faunal diversity [20, 67, 69, 93]. At up to 9 meters long, *Saurosuchus* was a large-bodied quadruped with erect posture, and was one of the largest terrestrial predators known worldwide from the Late Triassic [67, 69, 94–96]. *Saurosuchus* represents about 2.4% of faunal diversity in the Cancha de Bochas Member of the Ischigualasto Formation [20, 67, 69].

#### *Sillosuchus longicervix* PVSJ 085 (Figs 5A, 5B and 12)

The *Sillosuchus* femoral sample was taken from the poorly preserved holotype specimen (PVSJ 085). The femur is complete but crushed at proximal and distal ends [113]. Although most regions of the external cortex are brecciated, the diaphysis preserves patches of observable microstructural detail (Figs 5A, 5B and 12). The medullary cavity is crushed, making the nature of the medullary space difficult to ascertain (Fig 12A and 12B). The lack of trabecular components and the preservation of an endosteal lamellar IFS in the deepest cortex indicate that the medullary cavity was probably open (Fig 12B and 12C). A few large erosion rooms are restricted to the deep perimedullar cortex, but secondary osteons appear to be absent (Fig 12B and 12C). Isolated patches of compacted coarse cancellous bone, an endosteally derived tissue formed through the compaction of bony trabeculae, are also present (Fig 12C). Highly vascularized FLB comprises the cortex. Throughout the sample, circular and longitudinal primary osteons interweave in a laminar vascular pattern (Fig 12B, 12D and 12E), even at the most superficial periosteal surfaces (Fig 12F). The sample is devoid of annuli and LAG (Fig 12B and 12D–12F), and the absence of an EFS or any signature of increasing histological organization at the periosteal border supports the hypothesis that this large-bodied *Sillosuchus* specimen was still actively growing at the time of death (Fig 12F).

*Sillosuchus* shares a primary cortex dominated by highly vascularized FLB with *Poposaurus* [137], but exhibits a distinctive laminar vascular pattern that is more similar to the pattern observed in *Fasolasuchus* [135]. The smaller-bodied *Poposaurus* also differs from *Sillosuchus* in that it has a growth pattern interrupted by occasional zones of PFB accompanied by at least seven cyclical LAG [137]. *Sillosuchus* and *Poposaurus* differ from *Effigia*, a smaller-bodied relative in which cortices are dominated by PFB with simple longitudinal and reticular primary osteons vascular canals, with only localized deposits of FLB [132]. Our data indicate that *Sillosuchus* exhibits no histological evidence for slowed growth at the periosteal surface (e.g., the transition to slower growing PFB and/or LFB, appearance of annuli and/or LAG, and/or reduction in vascular density and anastomosis toward the periosteum), and was thus still actively growing at the time of death. *Sillosuchus* is unique among sampled pseudosuchians, including other closely related poposaurids and more distantly related taxa like *Saurosuchus* (see below), because it maintained continuous high rates of bone deposition throughout a significant part of ontogeny.

#### *Saurosuchus galilei* PVSJ 047 (Figs 5C, 5D and 13)

The femur (PVSJ 047) of *Saurosuchus galilei* exhibits a broad open medullary cavity lined by layers of endosteal lamellar bone in an IFS that cross-cut primary bone in the deep cortex (Fig 13A–13C). Signatures of bone remodeling are pervasive in the deep cortex of PVSJ 047, where areas of primary bone are overprinted with wide open erosional bays that are often lined with thin layers of centripetally deposited LFB (Fig 13D). Bone resorption extends into the mid-cortex. Sparse secondary osteons are most pervasive in the deep cortex and extend into the external cortex in some areas. That said, remodeling does not completely obliterate the primary bone signature in most regions of the cortex (Fig 13D–13F). Remodeling is more intensive in the thicker anterior cortex, with more frequent erosion rooms that extend toward the outer cortex, accompanied by denser, more highly interwoven, and more immature primary osteons in an FLB context (Fig 13E). An anterior external muscle scar is reflected in tissue microstructure by an area of higher vascularization and intensive remodeling that extends closer to the external cortex in PVSJ 047 (Figs 5C, 5D and 13A). The highly vascularized woven cortex is perforated by an interweaving network of mature longitudinal and circular primary osteons with occasional radial and reticular anastomoses in a sub-laminar or laminar pattern (Fig 13E). FLB deposition is punctuated by at least six mid-cortical LAG throughout ontogeny; cracks in the mid- and outer cortex follow several of these LAG in the cross-section (Fig 13B and 13E), and some cannot be traced completely around the element. Bone organization within each growth cycle transitions from highly vascularized FLB to more highly organized PFB dominated by sparse longitudinal primary osteons (Fig 13E), followed by deposition of a LAG. Each LAG is followed by an abrupt resumption of laminar FLB deposition in the subsequent cycle, in which a wide lamina of woven bone with disorganized osteocyte lacunae immediately follows LAG formation and signals the recovery of faster, more disorganized FLB deposition (Fig 13E). Growth cycles become thinner toward the periosteal surface of the element. Though the periosteal surface is poorly preserved, where it is visible areas of PFB and LFB with longitudinal primary osteons are common, and likely indicate attenuation of growth as PVSJ 047 approaches skeletal maturity. That said, in spite of the very large size of this specimen, PVSJ 047 lacks an EFS (Fig 13F).

Primary bone tissue histology in *Saurosuchus* is comparable to that of some other large bodied pseudosuchians (e.g., *Sillosuchus*, *Batrachotomus*, *Effigia*, *Postosuchus*, *Decuriasuchus*), especially with regard to the dominance of FLB and secondary remodeling that, at least in some individuals, extends into the mid-cortex [17, 132–136]. *Sillosuchus* and *Saurosuchus* both exhibit more frequent anastomosing networks of laminar primary osteons when compared to other pseudosuchians, where cortices tend to be dominated by unidirectional primary osteons that generally lack abundant anastomoses [17, 132–136]. *Saurosuchus* lacks an EFS, just as in *Batrachotomus*, *Decuriasuchus*, indicating that these particularly large-bodied species likely continued to increase body size on their paths to skeletal maturity. *Saurosuchus* also shares the presence of cyclical mid-cortical growth marks associated with localized areas of PFB with most other sampled pseudosuchians [17, 132–136].

### Archosauria—Crocodylomorpha

#### *Trialestes romeri* PVSJ 368 (Figs 5E, 5F and 14)

*Trialestes romeri* is one of the earliest known members of non-crocodyliform crocodylomorphs [97, 98]. Its anatomy suggests that it was an active terrestrial carnivore capable of cursorial locomotion [97–99]. *Trialestes* fossils account for just 0.1% of vertebrate faunal diversity recovered from the Cancha de Bochas Member of the Ischigualasto Formation [20, 67, 69]. Our data for *Trialestes* are the first for the genus, and add to the previously known histological patterns observed in other basal crocodylomorphs, including the humerus of *Terrestrisuchus* [17, 18], the femora of *Hesperosuchus* [5, 17, 18] and *Saltoposuchus* [138], and the tibia and fibula of an unnamed large bodied South African crocodylomorph [BP/1/8484, 139]. There is no femur associated with the holotype of *Trialestes* [97], but a referred specimen indicates a maximum femoral length for previously studied *Trialestes* of just 20.4 cm [97, Martínez, pers. comm]. The *Trialestes* femur in our sample (PVSJ 368) is associated with a partial skull, axial column, shoulder and pelvic girdles, and forelimb and hindlimb elements that share autapomorphies with the holotype [97, Martínez, pers. comm]. Femur PVSJ 368 is more than twice the length of femora from other known *Trialestes* specimens [97] (Table 1; 42 cm).

The right femur of *Trialestes romeri* (PVSJ 368) exhibits an open medullary cavity lined by a thin border of endosteal LFB forming an IFS (Fig 14A–14C). The posterior margin of the element is not preserved. Preserved regions of the cortex lack signatures of primary bone remodeling; erosion rooms and secondary osteons are absent (Fig 14B and 14C). Highly vascularized primary FLB dominates the cortex. Abundant interweaving circular and longitudinal primary osteons form a laminar vascular network (Fig 14D). In some areas occasional radial and reticular anastomoses indicate a sub-plexiform pattern (Fig 14F). The anteromedial mid-cortex documents a zone of reticular vascularity, with abundant radially-oriented primary osteons that may indicate preferential directional growth (Figs 5E, 5F and 14A). In more superficial regions of the cortex primary osteons are mature, and vascular canals are quite narrow (Fig 14E and 14F). In the outermost regions of the cortex the dominant FLB transitions in places to narrow patches of nearly avascular LFB that cannot be traced around the cross-section (Fig 14E). Only sparse longitudinal primary osteons are present in this zone of reduced primary bone deposition (Fig 14F). More superficially, deposition of FLB with laminar vascularity resumes and continues unabated nearly to the external margin of the element. At the periosteal surface, osteocyte lacunae become aligned in parallel rows in a PFB/LFB context, as primary osteons shift to a more longitudinal orientation (Fig 14F). The EFS is absent indicating that though primary bone deposition may have been slowing, *Trialestes* was still growing when it perished (Fig 14F).

*Trialestes* femoral histology is most similar to that of the humerus of *Terrestrisuchus* [17] and the femur of *Saltoposuchus* [138]. These taxa share the presence of open medullary cavities devoid of cancellous bony trabeculae, but lined with endosteally derived LFB forming an IFS with BP/1/8484 [139], and contrast with the trabeculae-filled medullary cavity observed in *Hesperosuchus* [5, 17]. In *Trialestes*, *Terrestrisuchus*, and *Saltoposuchus* some regions of the cortex are wider than others, and the cortices are highly vascularized and become more organized toward the periphery. *Terrestrisuchus* and *Saltoposuchus* exhibit a predominance of longitudinal primary vascular canals within an FLB context, whereas *Trialestes* exhibits an interweaving vascular network, potentially indicating a relatively faster bone depositional rate. Where *Terrestrisuchus* exhibits a single mid-cortical LAG, *Saltoposuchus* and *Trialestes* exhibit annuli. *Saltoposuchus* exhibits two zones of increased primary bone organization, one in the mid-cortex and one near the periosteal surface. In *Trialestes* a single annulus is situated near the periosteal surface. These data contrast with those for *Hesperosuchus* [17] and BP/1/8484 [139], in which primary cortices exhibit slower growing PFB and LFB punctuated by more regular LAGs. Interestingly, the only basal crocodylomorph that exhibits significant remodeling by secondary osteons is *Hesperosuchus* [5, 17]. None of these taxa exhibit a peripheral EFS, though BP/1/8484 exhibits peripheral LAGs that are increasingly more closely spaced [139].

### Archosauria—Dinosauria

Early dinosaurs represent about 11% of the vertebrate faunal diversity known from the HEH biozone of the Cancha de Bochas Member [20, 67, 69]. Known dinosaur taxa include the basal saurischians *Herrerasaurus ischigualastensis* [85, 86] and *Sanjuansaurus gordilloi* [87, 88], which co-occur and are the largest-known dinosaurs in the Ischigualasto Formation [67, 69, 76, 85–88]. Four other dinosaurs are known from the member, and all were small bodied species less than 3 meters in length. These include sauropodomorphs (*Eoraptor lunensis*, *Chromogisaurus novasi*, *Panphagia protos)* [67, 81–83, 140] and the small-bodied theropod *Eodromaeus murphi* [67, 83]. *Pisanosaurus mertii*, from the uppermost horizons of the Ischigualasto Formation, may represent a fifth early diverging ornithischian dinosaur [141–145], but recent work suggests that it may instead be a non-dinosaurian dinosauriform [146]. It comes from a different unit within the Ischigualasto Formation, and it was not sampled for this study. Femora are not yet known for *Panphagia* and *Saturnalia* so we have also excluded these taxa [67]. Femoral histologies are described below for *Herrerasaurus*, *Sanjuansaurus*, *Eodromaeus*, *Eoraptor*, and *Chromogisaurus*. These histological descriptions are the first for *Sanjuansaurus* [87–88], *Eodromaeus* [83], *Eoraptor* [60, 82, 84], and *Chromogisaurus* [81, 82], and supplement existing histological descriptions of the humerus and tibia of *Herrerasaurus* [17, 18, 147]. Our sampled *Herrerasaurus* femur (PVSJ 614) is from a similarly sized individual to those previously studied histologically (Table 1) [17, 18, 76, 147]. The *Eoraptor* femur in our sample is derived from a referred specimen (PVSJ 559) that is slightly larger than the adult holotype (Table 1) [84]. For all other early dinosaurs in our sample, only the single holotype femur is known.

#### *Sanjuansaurus gordilloi* (PVSJ 605) (Figs 6A, 6B and 15)

The holotype right femur of *Sanjuansaurus gordilloi* (PVSJ 605) is brecciated, but details of general primary bone growth patterns and bone microstructure are discernible throughout the cortex (Figs 6A, 6B and 15) [87, 88]. The medullary cavity is open and lined with a few thin avascular endosteally derived lamellae forming an IFS (Fig 15A–15C). Though the element is poorly preserved, erosion rooms, secondary osteons, LAGs, and annuli appear to be absent throughout the cortex where histological structure can be clearly observed (Fig 15C and 15D). Instead, highly vascularized primary FLB forms the entire cortex. Reticular vascularization dominates in the deeper regions of the cortex of PVSJ 605, and primary osteons are mature and highly infilled. Laminar vascularity becomes more common in the mid-cortex, with interweaving circular and longitudinal primary osteons uninterrupted by growth marks (Fig 15D and 15E). Where preserved, the periosteal surface becomes more organized as patches of PFB and longitudinal primary osteons are more common, pointing to attenuation of growth as skeletal maturity is reached (Fig 15F). The EFS is absent in PVSJ 605.

#### *Herrerasaurus ischigualastensis* PVSJ 614 (Figs 6C, 6D and 16)

The left femur of *Herrerasaurus ischigualastensis* (PVSJ 614) is slightly crushed, and some of the bony cortex is displaced into a relatively small but open medullary cavity (Figs 6C, 6D, 16A and 16B). The medullary cavity is lined by distinctive layers of avascular endosteal LFB forming an unusual two-part IFS (Fig 16C). The deepest cortical layer exhibits a few thin internal lamellae with highly organized lamellar bone fiber orientations. Slightly more externally, a single row of wide and open vascular channels is intercalated between these internal lamellae and another group of 5–6 thin highly organized external lamellae (Fig 16C). The interspersed vascular spaces are suggestive of remnant partially eroded and remodeled medullary bone. This region of perimedullar endosteal remodeling cross cuts the deepest preserved region of the cortex (Fig 16C). In the deepest regions of the cortex primary bone tissue is completely obliterated by multiple generations of well-developed secondary osteons forming dense Haversian bone (Fig 16C and 16D). This remodeling doesn’t extend beyond the deep cortex, and secondary osteons are absent in the middle cortex. The primary cortex is dominated by well-developed FLB characterized by dense vascularity in laminar and reticular interweaving networks (Fig 16E). This cortical structure is consistent throughout the cortex, though in the mid-cortex laminar bone tends to become slightly more dominant (Fig 16E). The element is devoid of cortical growth marks. At the periosteal surface vasculature decreases slightly and becomes dominated with immature longitudinal and sparse circular primary osteons. The preserved posterolateral region of the femur exhibits patches of PFB that are either dominated by longitudinal primary osteons or are avascular (Fig 16F). The anterior, medial, and lateral periosteal surfaces, where preserved, are characterized by abundant vasculature and immature primary osteons, indicating ongoing and rapid primary bone apposition in these regions (Fig 16B). The specimen lacks an EFS (Fig 16F).

These results are consistent with those reported by Ricqlès and colleagues [17, 18], specifically in relation to the densely vascularized primary fibrolamellar cortices. In other studied *Herrerasaurus* specimens vascular networks tend to be plexiform, with an abundance of circular vascular canals instead of the laminar-reticular pattern that dominates femur PVSJ 614. Moreover, thin layers of avascular endosteal bone line the medullary cavity to form an IFS in other sampled specimens, and secondary osteon formation is limited or absent [17, 147]. In contrast, PVSJ 614 exhibits a distinctive pattern of intensive secondary remodeling, with both endosteal lamellar bone forming the IFS and secondary osteons that are dense in the deep cortex. This may point to a somewhat older skeletal age for our sampled specimen relative to other sampled elements, or it may relate to a difference in functional environment (since other sampled specimens included a tibia and a humerus). Our sample includes a thin periosteal zone of avascular PFB/LFB, at least on the posterior surface of the element, that is similar to that observed in other sampled specimens. A similarly sized *Herrerasaurus* tibia exhibits an EFS, and also lacks cortical growth marks [147], but other sampled specimens lack definitive EFS, and thus were still actively growing when they died.

#### *Eodromaeus murphi* PVSJ 561 (Figs 6E, 6F and 17)

We sampled the right femur of the theropod *Eodromaeus murphi* (PVSJ 561) [83]. The element is only slightly crushed and bone microanatomy is very well-preserved (Figs 6E, 6F, 17A and 17B). The open medullary cavity is somewhat crushed, but endosteally derived avascular lamellar bone rings the regions of the medullary space that persist to form an IFS (Fig 17C). Aside from this evidence of bone erosion and redeposition around the medullary cavity there is no indication of intracortical bone remodeling. There are no resorption cavities, and secondary osteons are absent (Fig 17A–17D). Primary FLB is present throughout the cortex, with mostly primary osteons embedded within a woven bone matrix preserving abundant disorganized osteocyte lacunae (Fig 17C–17E). The density of organized LFB surrounding the many vascular canals imparts a sense of more detailed organization of primary tissue when the specimen is viewed at low magnification (Fig 17B). However, closer study at higher magnification reveals the disorganized nature of the woven component of the fibrolamellar complex (Fig 17C and 17D). Primary osteon orientations vary throughout the cortex of PVSJ 605. In some areas, vascularity is dominated by longitudinal primary osteons, while in other areas they interweave with circular, radial, and reticular osteons that record vascular patterns including reticular, laminar, and sub-plexiform patterns (Fig 17B–17D). In some regions primary osteons are closely packed, leaving only thin interspersed regions of woven bone surrounding them (Fig 17D). There are no LAGs or annuli in the cortex of the femur of *Eodromaeus*, indicating that primary osteogenesis continued unabated until death in this individual. The periosteal border exhibits transition in bone fiber orientation. In the external cortex, a very thin layer of much more highly organized PFB characterized by osteocyte lacunae arranged in thin parallel laminae is accompanied by a decrease in primary vasculature (Fig 17E and 17F). Vasculature in this final bone depositional interval of femur PVSJ 605 is much more sporadic, with mostly immature longitudinally-oriented primary osteons, including some that are open at the periosteal surface. Femur PVSJ 561 lacks an EFS and points to ongoing growth at the time of death (Fig 17E and 17F).

#### *Eoraptor lunensis* (PVSJ 559) (Figs 7A, 7B and 18)

The *Eoraptor* femoral sample was extracted from a referred right femur recovered 25 cm from the holotypic skeleton [84]. The element exhibits some breakage, but microanatomical details are well-preserved throughout. Femur PVSJ 559 exhibits a wide and open medullary cavity that is partially lined with centripetally deposited layers of endosteal lamellar bone forming an IFS (Fig 18A–18C). The deep cortical histology of this element is highly variable. In some areas the IFS lamellae ringing the medullary space overlap deep cortical deposits of primary fibrolamellar bone (Fig 18C). In other areas the IFS overlaps generations of secondary osteons that reworked an isolated patch of the deep cortex (Fig 18D). The adjacent endosteal lamellae are themselves indicative of extensive remodeling, with large (~550 microns) circumferentially oriented erosion rooms formed at the boundary between endosteal lamellae and older deposits of primary FLB (Fig 18B and 18D). These erosion rooms exhibit thin external layers of centripetally-deposited LFB (Fig 18B). An arc of compacted Haversian tissue persists between these layers of endosteally derived lamellar bone (Fig 18D). The cortex of PVSJ 559 can be divided into three regions that record transitions from more highly vascularized disorganized primary FLB for most of ontogeny to an interval of somewhat more organized lower vascularity PFB for an interval during later mid-ontogeny, and then back to a phase of more active growth in the latest recorded ontogenetic stage (Fig 18E). The deepest primary cortex exhibits highly vascularized primary FLB with circular and longitudinal primary osteons interweaving in a laminar pattern. In some regions this pattern transitions into one with an abundance of radial and more irregularly organized primary osteons, but always in the context of a woven bone matrix that exhibits densely packed osteocyte lacunae consistent with a standard fibrolamellar complex (Fig 18E and 18F). Moving superficially, a transition in primary bone depositional pattern is indicated by a temporary transition in bone mineral and vascular organization. In this region, bone mineral organization is predominantly parallel-fibered, with localized patches of even more highly organized LFB (Fig 18E and 18F), and vasculature is sparse and reduced to simple mature longitudinal primary osteons (Fig 18E and 18F). In the most superficial regions of the cortex, PVSJ 559 documents a return to a more disorganized FLB depositional pattern (Fig 18E and 18F). A sub-plexiform vascular network dominates this region of the cortex, with abundant circular, longitudinal, and radial primary osteons interweaving in a sub-plexiform pattern (Fig 18E). The *Eoraptor* femur PVSJ 559 lacks LAGs and a peripheral EFS, though the perturbations near the outer cortex are suggestive of the onset of growth rate attenuation (Fig 18F).

#### *Chromogisaurus novasi* (PVSJ 845) (Figs 7C, 7D and 19)

We sampled the holotypic partial right femur of *Chromogisaurus* [81, 82] (Figs 7C, 7D and 19). Bone microstructural data are well-preserved in the mid-diaphysis in spite of breakage at both ends and some overall compression and fracturing of the element. Femur PVSJ 845 exhibits an open medullary cavity devoid of trabecular bone (Fig 19A and 19B). At least four cycles of endosteal remodeling are preserved that include both endosteally-derived LFB as well as secondary osteons (Fig 19B and 19C). The most superficial of these LFB layers likely formed first during an early period of relatively early ontogenetic medullary drift and endosteal bone remodeling that obliterated earlier deposits of deep cortical primary bone tissue (Fig 19B–19D). Following the initial pulse of outwardly focused medullary drift, centripetal deposition of LFB would have resulted in a narrower medullary cavity (Fig 19C and 19D). Some of this endosteal LFB underwent a sequence of additional secondary remodeling as ontogeny proceeded, and was eventually replaced by dense Haversian bone (Fig 19B–19D). A second phase of bone erosion occurred more deeply and adjacent to the medullary cavity, resulting in deposits of LFB that cross-cut these earlier deposits of Haversian bone (Fig 19C). A similar cycle of bone erosion and redeposition occurs at least once more in the perimedullar space, with the formation of a third endosteal lamellar layer that is partially remodeled by secondary osteons (Fig 19C). Here, between the third and fourth cycles, large open erosional spaces are dispersed among secondary osteons and indicate the ongoing process of bone resorption and redeposition (Fig 19B). The fourth, final, and deepest signature of perimedullar remodeling is a bit more unusual (Fig 19B and 19C). Though in some regions the medullary cavity is lined by a fairly typical endosteal IFS (Fig 19C), in at least one area of the deepest cortex a zone of longitudinally vascularized woven fibered bone tissue lies deep to the most internal deposits of endosteal lamellar bone, and abuts a small region of the medullary cavity (Fig 19C). This patch of woven bone tissue deep to endosteal lamellae and lining the medullary cavity in *Chromogisaurus* exhibits some of the diagnostic features of both medullary bone, a sex-specific bone tissue produced in females during egg-laying [148, 149], as well as a paleopathological condition described for at least one other basal sauropodomorph [150, 151].

At the same time that these cycles of remodeling overprinted signatures of primary tissue in the deep cortex, appositional growth in more superficial areas continued. The primary cortex of PVSJ 845 is characterized by the continuous deposition of FLB vascularized by abundant longitudinal primary osteons with occasional circular anastomoses (Fig 19B and 19D–19F). Most superficially, no major transition in primary bone depositional pattern is indicated, though primary vascularization gradually becomes more uniformly dominated by unidirectional longitudinal primary osteons (Fig 19E and 19F). Intracortical LAGs and a peripheral EFS are absent from femur PVSJ 845 (Fig 19F). Appositional growth was unabated at the time of death for this *Chromogisaurus* individual.

## Discussion

Decades of qualitative comparative biology and experimental research on the bone histology of extant vertebrates have cemented our understanding that life history data are captured within bone tissue, and that homologous features preserved in the bone microstructure of fossil vertebrates allows for deductions about their relative growth strategies [see summaries in 1–5]. Our results add to the growing dataset that relates to questions that relate to how and when the generalized dichotomy observed in the distribution of bone tissues in living archosaurs evolved, first articulated by Ricqlès and colleagues [17, 18], and echoed by many other authors [e.g., 11, 15, 23, 24, 39–41, 60, 61, 101, 120, 121, 135, 152–157]. Namely, were the ancestors of crown-group archosaurs relatively fast or relatively slowly growing animals? Did the ancestors of crown-group archosaurs grow differently than closely and more distantly related faunal contemporaries? And what insights might data from early dinosaurs provide in understanding when dinosaurs first evolved their relatively rapid growth strategies? Our results on the femoral histology of a suite of coeval vertebrates, including a diversity of early dinosaurs from the Ischigualasto Formation of Argentina, directly address these questions.

First, we employ multiple metrics of maturity to assess relative ontogenetic status for each individual in the Ischigualasto sample. We then integrate histological data across all sampled Ischigualasto vertebrates to develop three “Growth Strategy Groups” (GSGs) that reflect a faunal perspective on broad similarities in ontogenetic bone growth patterns. With these groups established, we explore the complexity within groups, particularly as it relates to growth rate distinctions among closely related, anatomically similar taxa, as well as similarities among distant relatives with presumably similar ecological roles. We conclude the discussion with a brief consideration of how these new data pertain to the hypothesis of an early innovation of a uniquely “dinosaurian” growth strategy.

### Ontogenetic assessment in ischigualasto vertebrates

**Fig 20:**
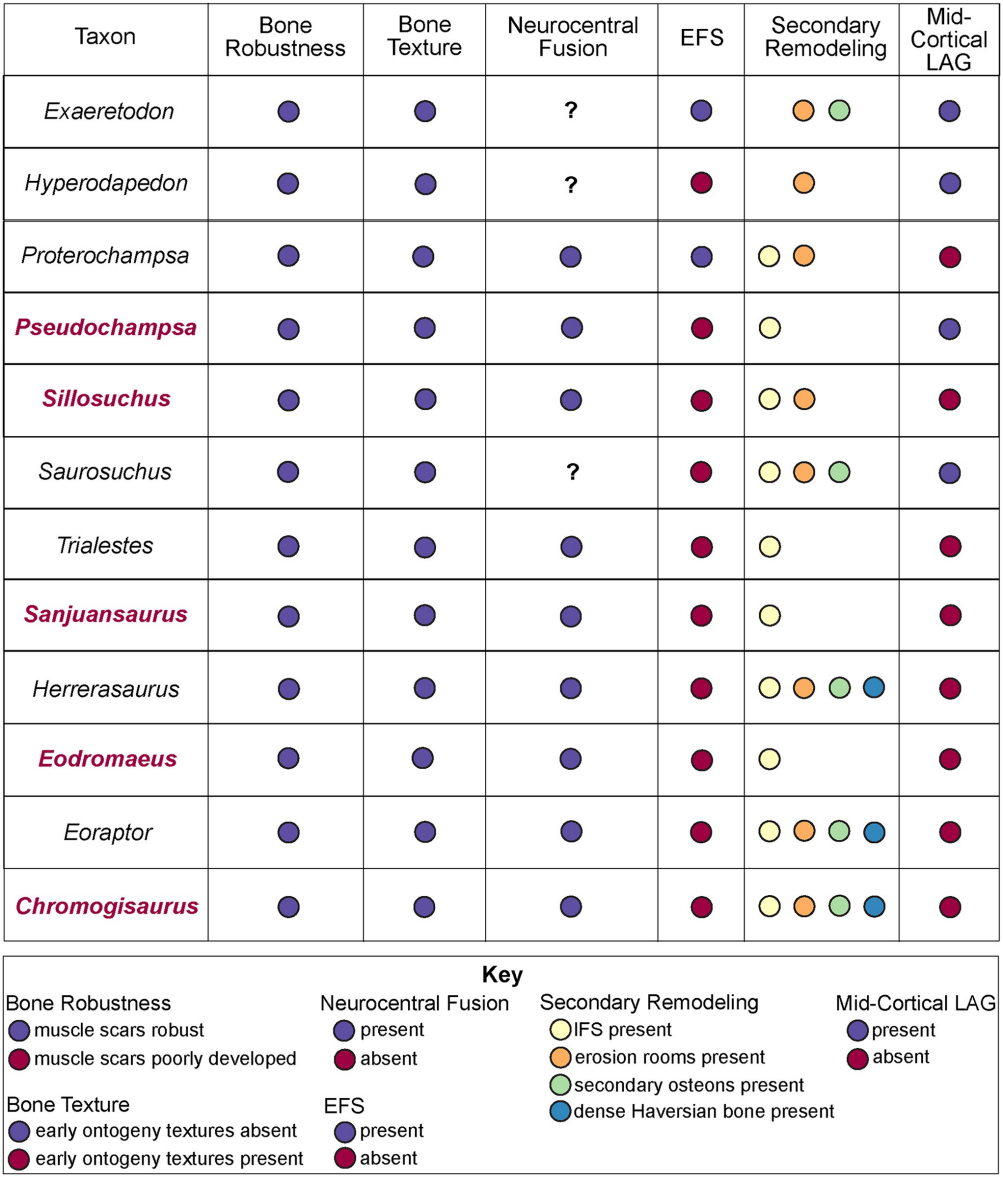
Anatomical and osteohistological signals of relative ontogenetic status in the ischigualasto vertebrate sample.

The disparate growth patterns, body plans, and life histories among extinct reptiles make it challenging to diagnose the ontogenetic status of the Ischigualasto taxa [1, 2, 4, 5, 59]. Often only a single method is used in the determination of relative maturity, and these assessments tend to be accompanied by ambiguous, ill-defined terminology relating to relative ontogenetic status (e.g., “early juvenile,” “late subadult”). Here we follow recommended best practices outlined by Griffin and colleagues [59], and use multiple metrics of maturity drawn from both gross morphology and osteohistology to assess ontogenetic status in the Ischigualasto sample. The preponderance of the evidence suggests that all of the individuals in our histological sample have grown beyond the earliest stages of ontogeny. Though just two individuals in the sample are skeletally mature (based on the presence of EFS), all record an appreciable record of growth that we consider to be characteristic for each taxon. Here we detail our findings for a number of commonly used ontogenetic assessment metrics (Fig 20).

Samples taken from holotype specimens known from only a single skeleton are indicated by a taxonomic name in ***red*** [81–83, 87, 88, 91–93]. Most other samples were drawn from larger specimens than those previously studied [89, 91, 94–96]. Ours are the first histological samples for *Eoraptor* [60, 82, 84], and *Trialestes* [97, 98]. These samples were purposely drawn from individuals with the largest known body sizes for the genus. In the cases of *Herrerasaurus* and *Hyperodapedon*, samples were drawn from large individuals equivalent in size to those previously examined [85, 86, 76, 90, 147]. Data on gross anatomical metrics of maturity are drawn from the primary literature and through personal observations by the authors [60, 81–98, 147]. Robustness refers to the development of muscle scars (purple = muscle scars are robust and strongly developed; red = muscle scars are poorly developed or undeveloped) [59,158, 159]. Bone texture refers to surficial longitudinal striations/porosity/fibrous textures that are common in early ontogeny (purple = absence of early ontogeny bone textures; red = presence of early ontogeny bone textures) [59, 160, 161]. Neurocentral fusion between vertebral centra and their neural arches occurs over the course of ontogeny and is a dependable indicator of skeletal maturity (purple = neurocentral fusion is present in sampled specimens or in smaller individuals of the same taxon throughout axial column; red = neurocentral fusion is absent in sampled specimens throughout axial column;? = specimen is not associated with vertebrae and neurocentral fusion cannot be assessed) [162–164]. The EFS indicates attainment of skeletal maturity (purple = EFS is present; red = EFS is absent) [1, 2, 5, 22, 23, 39–42, 59, 165, 166]. All taxa show evidence of secondary remodeling, which generally indicates more prolonged skeletal development (light yellow = presence of an IFS indicating medullary drift; orange = erosion rooms present; may contain some secondary infill; light green = presence of secondary osteons; blue = multiple generations of secondary osteons overlap to form dense Haversian bone) [1–6, 56, 59–63]. Mid-cortical LAG indicate periodic cessation of appositional growth throughout ontogeny (purple = mid-cortical LAG are present; red = mid-cortical LAG are absent). The absence of LAG does not necessarily indicate less than one year of bone deposition [1–5, 11–14, 28–33, 45, 47, 53–59].

The most common metric for assessing maturity in fossil reptiles is body size [59]. With the aim of accessing the longest record of growth for each taxon in our analysis, we sampled the largest individuals known for *Exaeretodon*, *Proterochampsa*, *Trialestes*, *Saurosuchus*, and *Eoraptor*. (Fig 20; Table 1) [60, 82, 84, 91, 94–98]. In the case of *Hyperodapedon* and *Herrerasaurus*, we sampled femora from individuals of equivalent size to those previously sampled by other authors (Fig 20; Table 1) [85, 86, 76, 90, 147]. For these two taxa larger samples do not exist, and it is important to note that previous studies found an EFS indicative of skeletal maturity in a sampled *Herrerasaurus* tibia [147]. Our sample also included five holotype femora for which only a single individual is known (*Eodromaeus*, *Chromogisaurus*, *Sanjuansaurus*, *Pseudochampsa*, and *Sillosuchus*). Femoral lengths for each of these holotype specimens suggest that they too experienced appreciable growth beyond hatching/early ontogeny (Fig 20, Table 1) [81–83, 87, 88, 91–93].

Other variables that inform our understanding of relative maturity among the Ischigualasto sample rely upon the external surfaces of appendicular elements. The first of these variables can be referred to as the ‘robustness’ of the postcranial skeleton [59]. Femoral bone scars resulting from muscle attachment are known to become more “robust” (rugose and prominent) with age [59, 160, 161]. Every individual in our sample exhibits clearly defined and robust muscle attachment sites [81–98]. The second variable relates to surface textures of long bones, which tend to become smoother as organisms grow older [59, 160, 161]. None of the bones we sampled exhibit the striated, fibrous, or porous bone textures typical of very young individuals [160, 161]. Although these external anatomical features do not serve as the ‘gold standard’ for ontogenetic assessment [59], the observed aspects of femoral surface textures and muscle scars are consistent with the premise that the Ischigualasto individuals sampled in this study progressed well beyond the earliest stages of their life histories.

The sutures between vertebral centra and their respective neural arches fuse or close over the course of ontogeny in reptiles, often in a sequence from posterior to anterior [59, 132, 162–164]. Though patterns of fusion vary among vertebrate groups, open neurocentral sutures are relatively dependable indicators of skeletal immaturity [59]. Nine of our Ischigualasto specimens were discovered in association with vertebrae that exhibit neurocentral closure throughout the axial column [17, 18, 60, 76, 81–88, 91–93,97, 98, 147]. Among them, the *Eoraptor* femur we sampled was associated with two dorsal vertebrae that exhibit closed neurocentral sutures; in addition, the *Eoraptor* holotype (PVSJ 512) is from a smaller skeleton that also includes a complete articulated axial column that exhibits neurocentral fusion [60, 82, 84]. Our samples of *Saurosuchus*, *Exaeretodon*, and *Hyperodapedon* were not associated with well-preserved axial elements, but they do exhibit a variety of other features consistent with advanced age (including size, surface texture, and histology).

Finally, osteohistological data also reveal important clues that inform our understanding of ontogenetic status in fossil vertebrates [1–14, 21–26, 59, 165, 166]. These data include shifting patterns of primary bone tissue and vascular organization, details of bone remodeling, and the occurrence of an EFS. The most obvious histological indicator of skeletal maturity is the EFS. We observed an EFS in *Proterochampsa* and *Exaeretodon*. All other taxa in our sample were still actively growing at the time of death (and thus skeletally “immature”). Even so, other histological data help us clarify their relative progression within the ontogenetic spectrum. Most other taxa document changes in primary tissue histology that suggest alterations in bone appositional rates over time; the only exception is *Eodromaeus* (Fig 17). Another signature of more advanced ontogenetic status for all taxa, including *Eodromaeus*, is the presence of secondary remodeling [1–6, 56, 59–63]. Secondary remodeling includes the formation of erosion rooms and/or mid-cortical secondary osteons, formation of an IFS signaling medullary drift, and the presence of dense Haversian tissue in *Eoraptor* and *Herrerasaurus*. Finally, although some Ischigualasto taxa do record cyclical mid-cortical LAG, most do not (Fig 20). The absence of LAG could be interpreted as indicative of individuals less than a year old, but given the other metrics that indicate more advanced ontogenetic stages as well as the well-known complications relating to LAG deposition [1–5, 11–14, 28–33, 45, 47, 53–59], we deem this interpretation unlikely. Collectively, these osteohistological characters indicate that the sampled Ischigualasto individuals had progressed beyond the earliest stages of their ontogenies [1–14, 21–26, 59, 165, 166].

Given the evidence outlined above, we contend that the individuals in our sample exhibit an informative record of growth spanning a significant ontogenetic interval. All show evidence of having moved beyond the earliest phases of growth, and the majority document transitions in their bones and skeletons that indicate some degree of skeletal maturation. That said, only two sampled taxa can be considered definitively skeletally mature—*Exaeretodon* and *Proterochampsa*. While our categorizations of “skeletally mature” and “skeletally immature” may not be as exacting as desired, we are confident that the Ischigualasto sample provides ample data to compare among these taxa in order to assess broader faunal growth patterns, which are developed in the following section.

### A faunal perspective: Growth strategy groups

**Fig 21:**
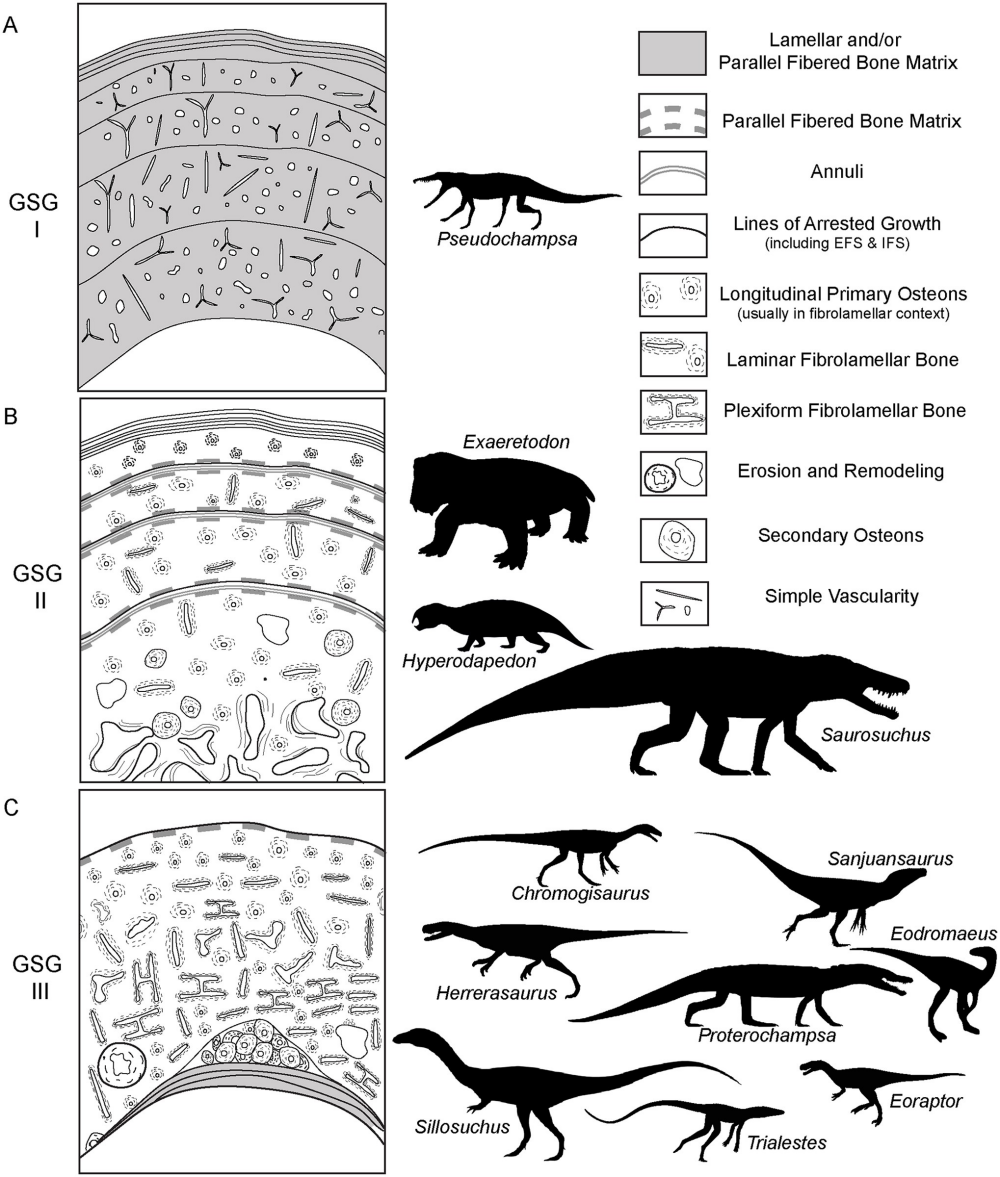
Growth strategy groups based on bone histology.

Here we employ bone histological features to categorize each taxon within one of three different “Growth Strategy Groups” that provide a framework for comparing relative growth patterns among the Ischigualasto paleofauna. Each GSG includes taxa that exhibit broad similarities in life history and ontogenetic growth strategies based upon observed bone growth patterns diagnosed by specific bone microstructural features. These include dominant bone fiber organization, continuous or cyclical bone deposition and the nature of growth marks if present, density/pattern of anastomosing primary vasculature, and density/types of secondary bone remodeling, including secondary osteons and/or the IFS (Fig 21, Table 2). We present the GSGs from slowest growing to fastest growing, and include some observations that arguably pave the way for future comparative work with additional samples.

**Table 2:** Growth strategy groups. Histological features used to diagnose each of the three Growth Strategy Groups are outlined here, along with group membership.

Growth Strategy Group	Diagnostic Histological Characteristics	Ischigualasto Vertebrates
GSG I—Slower and Cyclical	*Dominant fibrillar organization*: PFB and/or LFB *Vascularity*: Simple primary vascular canals with no osteonal infills and/or unidirectional primary osteons with limited anastomosis *Cyclicity*: Cyclical bone deposition; cycles demarcated by LAG and/or annuli *Remodeling*: Secondary osteons absent; remodeling only evident as IFS	*Pseudochampsa*
GSG II—Faster, But Cyclical	*Dominant fibrillar organization*: FLB, may have some regular mid-cortical regions of PFB or LFB *Vascularity*: Primary osteons with limited anastomosis *Cyclicity*: Cyclical bone deposition; cycles attenuate from deep to superficial, and are bounded by LAG and/or annuli *Remodeling*: Sparse secondary osteons limited to deep cortex; IFS may be present	*Exaeretodon* *Hyperodapedon* *Saurosuchus*
GSG III—Continually Fast	*Dominant fibrillar organization*: FLB *Vascularity*: Primary osteons abundant and interweave in complex but varying patterns *Cyclicity*: Bone deposition continuous until late ontogeny; late ontogeny shift to PFB/LFB, areas of avascularity, and/or EFS form. No mid- or deep-cortical growth marks Outer 1/3 of element may exhibit irregular mid-ontogeny reductions in bone appositional rate recorded by PFB/LFB, areas of reduced vascularity or avascularity, or annuli, but not by regular mid-cortical LAG *Remodeling*: Secondary osteons may be present, sometimes as dense Haversian bone; Mid-cortical remodeling may be present; IFS may be present.	*Proterochampsa* *Sillosuchus* *Trialestes* *Herrerasaurus* *Sanjuansaurus* *Eodromaeus* *Eoraptor* *Chromogisaurus*

Ischigualasto vertebrates exhibit some overall similarities in bone tissue organization that reflect broad congruence in growth pattern. These three Growth Strategy Groups (GSG) reflect these shared features, though we note that within each, significant variation exists that should not be overlooked. (**A**) Only one sampled Ischigualasto vertebrate currently fits into the slowest growth group, GSG I. *Pseudochampsa* exemplifies a slower overall growth pattern than other Ischigualasto vertebrates because it exhibits more organized bone mineral throughout the cortex, regular cessations in bone apposition recorded by regular LAG, simple vascular canals rather than primary osteons, and limited secondary remodeling focused only on the medullary cavity. (**B**) Three Ischigualasto vertebrates fit within GSG II, a group that exhibits elevated rates of primary bone deposition related to GSG I, but continues to experience regular cessation of growth throughout ontogeny recorded by mid-cortical growth marks, including annuli and LAG. This group includes two herbivores, *Exaeretodon* and *Hyperodapedon*, as well as the large-bodied predatory *Saurosuchus*, a loricatan pseudosuchian. Each of these taxa shares the deposition of well-vascularized fibrolamellar bone tissue throughout most of ontogeny, as well as intracortical secondary remodeling that results in the formation of secondary osteons. (**C**) GSG III includes taxa that grow at similar relative rates to those in GSG II, except that in GSG III growth is continuous until late in ontogeny when the first signatures of growth rate reduction are recorded in transition from fibrolamellar bone to PFB and/or LFB in the outer cortex. At least three of the dinosaurs in this group exhibit dense Haversian patterns of deep cortical remodeling that occurs in a unique, arc-like morphology, sometimes sandwiched between layers of endosteally derived LFB that represent earlier phases of medullary drift in the form of bone erosion and redeposition. (*Chromogisaurus*, *Eoraptor*, *Herrerasaurus*). Organismal silhouettes were downloaded from Phylopic.org. See S1 File for credits and copyright data for individual silhouettes.

#### Growth strategy group I—Slower & cyclical

Just one taxon resides in GSG I, which is reserved for Ischigualasto vertebrates that exhibit a dominant pattern of histological features indicative of relatively slow growth rates (Fig 21A, Table 2). Future analyses of additional taxa may increase membership within this group. Slow growing and highly organized PFB and LFB characterizes *Pseudochampsa* and signals an overall slower rate of bone deposition in this taxon when compared to other sampled Ischigualasto taxa. In extant vertebrates these tissues have been experimentally shown to form at apposition rates of 5 μm or less per day [7]. Primary vasculature is abundant, but canals are simple and generally lack centripetal deposition of osteonal bone. *Pseudochampsa* experienced regular drops in bone depositional rate signaled by changing bone mineral organization from PFB to LFB, coincident with reduced diameter, density, and directionality of primary vasculature as well as the presence of highly organized, tiny, and diminished osteocyte lacunae. These reductions in primary bone deposition occur before and after multiple LAGs indicating regular and recurrent cessation of growth throughout ontogeny. Bone organization surrounding LAGs demonstrates a gradual slowing and cessation, and a similarly gradual resumption of primary osteogenesis. At least 4–5 cycles that narrow from the deeper to more superficial cortex indicate a regimented periodicity and regular attenuation of primary bone apposition over life history for this *Pseudochampsa* individual. The cortex lacks evidence of bone remodeling in spite of the relatively mature ontogenetic status of this specimen (Fig 20). It is possible that a broken fragment floating within the open medullary cavity indicates that some endosteal perimedullar erosion and redeposition occurred, but this is uncertain. These data are most similar to those recorded in the femoral histology for *Chanaresuchus bonapartei* from the Los Chañares Formation of Argentina [124]. These distinctive patterns set *Pseudochampsa* and *Chanaresuchus* apart from other sampled proterochampsians (see below), as well as other sampled Ischigualasto taxa, and point to a cyclical reduced rate of osteogenesis throughout ontogeny [124].

### Growth strategy group II—Fast, but punctuated

*Exaeretodon*, *Hyperodapedon*, and *Saurosuchus* are members of GSG II (Fig 21B, Table 2). All three taxa exhibit primary cortices dominated by FLB, consistent with an overall elevation of growth rate relative to the PFB and LFB-dominated cortices characteristic of GSG I. Studies of extant vertebrates reveal that the woven component of FLB forms rapidly at widely ranging apposition rates between ~5 to 171 μm per day [7–10, 167]. It is important to note that primary vascular patterns vary widely among these three taxa. It is likely that the more mature primary osteons with fewer anastomoses observed in *Exaeretodon* indicates a somewhat lower rate of bone deposition than the highly interweaving laminar vascular networks that occur in *Saurosuchus* and *Hyperodapedon* (Figs 8, 9 and 13). Despite the dominance of FLB among these disparate taxa, each exhibits annuli/LAG that punctuate growth throughout the cortex, similar to *Pseudochampsa* in GSG I. These signals indicate that throughout ontogeny taxa within GSG II experienced cyclicity in bone depositional rates. *Exaeretodon* experienced at least two decelerations in growth rates captured by circumferential growth marks, while *Hyperodapedon* underwent four cyclical cessations and resumptions of primary apposition. *Saurosuchus* paused bone deposition at least six times during ontogeny. Each decrease in growth rate is recorded by a shift from FLB dominated by unidirectional primary osteons to more poorly vascularized or avascular PFB with annuli, and finally to a LAG. LAG in *Exaeretodon* and *Hyperodapedon* are followed first by a thin layer of PFB followed by FLB dominated by wide and open primary vascular canals that indicate the resumption of faster growth. This repeating pattern in these two taxa indicates that growth rates didn’t resume quickly following cessation, but instead ramped back up over some period of time. In contrast, recovery of elevated growth rates in *Saurosuchus* occurred less gradually, with each LAG followed by an abrupt resumption of laminar FLB deposition. In *Saurosuchus* and *Exaeretodon* the width of FLB deposits narrows between cyclical growth marks, from deep to superficial. This change in cycle thickness indicates that primary apposition in each active growth cycle decreased as these taxa aged. In *Hyperodapedon* this pattern isn’t as straightforward; one mid-cortical cycle is narrower than those deposited later (Figs 4C, 4D, 9A and 9B). For all three taxa most appositional growth occurred within the first few growth cycles, probably in the first few years of life.

Each member of GSG II also exhibits signatures of mid-cortical secondary remodeling supporting the contention that they were at advanced ontogenetic age, and also likely experienced somewhat elevated rates of bone metabolism [e.g., 1–5, 56, 60–63, 105, 119]. For *Exaeretodon* and *Saurosuchus*, primary bone remodeling is well underway in the perimedullar cortex, where both erosion rooms and sparse secondary osteons overprint primary bone tissue. In *Hyperodapedon*, secondary remodeling has not yet progressed to this point, and is signaled instead by abundant perimedullar and rare mid-cortical erosion rooms with some centripetal deposition of lamellar bone (Fig 9B and 9C) that heralds the onset of secondary osteon formation. *Saurosuchus* exhibits the most widespread cortical remodeling among the Ischigualasto sample, with secondary osteons present even in the outer cortex. That said, when compared to taxa in GSG III (see below), remodeling in *Saurosuchus* is still relatively sparse, with an absence of dense Haversian tissue despite the large body size (and likely advanced age) of this specimen.

*Exaeretodon* exhibits an EFS that signals the attainment of adult body size. Though *Hyperodapedon* and *Saurosuchus* lack the EFS, the slowing of bone deposition recorded in the periosteal histology likely signals a determinate growth strategy consistent with that of other rhynchosaurs and pseudosuchians sampled thus far [118, 119, 132–135]. Elevated growth rates in early ontogeny are hypothesized to have facilitated the evolution of larger adult body size in taxa like *Exaeretodon* relative to other traversodontids [105, 107], and an early ontogeny growth spurt may have played a similar role in fueling large body size in *Hyperodapedon* [19, 118, 119] and *Saurosuchus*.

#### Growth strategy group III—Fast & continuous

GSG III includes the proterochampsian *Proterochampsa*, the poposaurid *Sillosuchus*, the crocodylomorph *Trialestes*, and all of the sampled dinosaurs, including *Herrerasaurus*, *Sanjuansaurus*, *Eodromaeus*, *Eoraptor*, and *Chromogisaurus* (Fig 21C, Table 2). GSG III taxa share continuous deposition of FLB with abundant anastomoses of dense vascularity regularly approaching or attaining laminar and plexiform patterns during growth history. In contrast with taxa in GSG I and II, members of GSG III lack cyclical growth mark evidence of mid-ontogeny cessation or significant decelerations of appositional growth until later in ontogeny. Though these patterns might seem to be an indication that GSG III merely designates a group of relatively young individuals, comprehensive metrics of skeletal maturity actually point to relatively older ontogenetic status for all GSG III taxa (Fig 20).

Evidence of bone remodeling is present in all GSG III taxa, ranging from simple medullary drift/formation of the IFS to scant intracortical primary bone resorption and/or sparse secondary osteon formation to dense Haversian bone (Fig 20). *Trialestes*, *Sanjuansaurus*, *Eodromaeus* exhibit perimedullar IFS, but lack any evidence of intracortical remodeling (e.g., erosion rooms and/or secondary osteons). *Sillosuchus* lacks secondary osteons, but exhibits both the IFS and perimedullar and mid-cortical erosion rooms that are the harbingers of ongoing bone resorption. *Proterochampsa* exhibits an IFS, sparse deep cortical secondary osteons, and mid-cortical erosional bays, all indicative of a high rate of bone metabolism. The dinosaurs *Herrerasaurus Eoraptor*, and *Chromogisaurus* exhibit the IFS, intracortical erosion rooms, some deep cortical secondary osteons, and are the only Ischigualasto vertebrates to exhibit intensive remodeling that resulted in the formation of perimedullar/deep cortical dense Haversian bone (Fig 16B and 16C; Fig 18B and 18C; Fig 19B–19D).

Most GSG III taxa are characterized by an external cortical transition to occasional patches of more organized patches of PFB and/or LFB with longitudinal primary vascular canals indicative of slowing primary osteogenesis. These shifts may relate to the approach of skeletal maturity in later ontogeny, but appositional growth was ongoing, albeit at somewhat attenuated rates in most taxa. It is interesting to note that among the GSG III taxa *Chomogisaurus* does not exhibit a major shift in bone appositional pattern consistent with cessation of growth and skeletal maturity, in spite of the presence of an perimedullar tissue that exhibits a suite of diagnostic features that are more consistent with medullary bone than with pathological bone [148–151]. Medullary tissue is present at the endosteal surface in sexually mature females during egg-formation [148, 149], in constrast with pathological bone, which is deposited at both endosteal and periosteal surfaces [150, 151]. The potential presence of medullary bone can only be confirmed with the sampling of additional elements. If present in the absence of the EFS, it would signal an intriguing decoupling of sexual and somatic maturity in this early sauropodomorph.

### Histological hints of ecological complexity

The three GSGs described above were established using clear distinctions in relative patterns of ontogenetic growth (Fig 20, Table 2). There are also important histological variations recorded *within* each GSG, including distinctions in the patterns and density of primary vascularity and the intensity and nature of bone remodeling (e.g., mid-cortical secondary resorption vs. perimedullar resorption vs. dense Haversian bone) suggestive of more subtle differences in growth history. Our results are consistent with those of previous workers that histological variation exists even among closely related taxa, and this variation serves as a caution against inferring common growth patterns simply because two taxa may be phylogenetically closely related [18, 105, 107, 108, 119, 124]. Furthermore, our results also reveal that a number of the more distantly related taxa in our sample thought to share broadly similar ecological roles also share features of their bone tissue and growth trajectories.

Despite their similar sizes, morphologies, and presumed ecological roles in the Ischigualasto ecosystem, *Proterochampsa* and *Pseudochampsa* show dramatic distinctions in their femoral bone tissues that place them in the slowest and fastest growing GSGs, respectively (Fig 20, Tables 1 and 2) (Figs 10 and 11) [121, 124]. Perhaps, as suggested for *Chanaresuchus*, the pattern in *Pseudochampsa* relates to a semi-aquatic habitat preference, with the compact cortex and reduced medullary cavity signaling an adaptation related to reduction in buoyancy and aquatic lifestyle [e.g., 124]. Histological differences observed here for *Pseudochampsa* and *Proterochampsa* may eventually help test hypotheses of niche-partitioning among these closely related and morphologically comparable taxa within the Ischigualasto ecosystem [e.g., 168–169].

A similar observation can be made for the large bodied pseudosuchians *Sillosuchus* and *Saurosuchus* (Figs 12 and 13). Though the sampled femora were derived from individuals of similar large body sizes (Table 1), and both share features indicative of appreciative ontogenetic growth (Fig 20), they each exhibit unique general growth patterns, relegating them to distinct GSG (Table 2). Even though both taxa deposit rapidly growing highly vascularized fibrolamellar bone, *Saurosuchus* does so cyclically while *Sillosuchus* does so continuously. *Saurosuchus* paired cyclical primary bone deposition with intensive secondary remodeling and a late ontogeny reduction in primary appositional rate indicative of the approach of skeletal maturity. Our sample from *Sillosuchus* contrasts with the record for *Saurosuchus*, and exhibits continuous ongoing growth with only limited mid-cortical erosion and unabated appositional growth.

Histological differences also exist in the closely related and ecologically similar dinosaurian carnivores *Herrerasaurus* and *Sanjuansaurus* (Figs 15 and 16). Our femoral samples were derived from large-bodied individuals of almost identical size (Table 1) that exhibit rapid, continuous growth. That said, subtle distinctions in primary bone vasculature and patterns of bone remodeling highlight differences in growth dynamics. Although *Herrerasaurus* exhibits dense Haversian bone in the deepest cortex, it was still growing at the time of death. In contrast, though *Sanjuansaurus* exhibits changes in peripheral bone vascularity consistent with a reduction in bone appositional rate, it completely lacks mid-cortical secondary remodeling.

Our samples for *Eodromaeus*, *Eoraptor*, and *Chromogisaurus* also come from similarly-sized individuals (femur lengths for all ~16 cm), and once again, bone tissue distinctions may indicate subtle differences in relative ontogenetic status (Figs 17–20, Table 2). While *Eoraptor* exhibits both intensive deep cortical remodeling and a subtle peripheral perturbation in growth that may signal a more advanced state of skeletal maturity (Fig 18), *Chromogisaurus* shares patterns of deep cortical remodeling, including potential medullary bone, but lacks evidence for a marginal decrease in appositional growth (Fig 19). *Eodromaeus* lacks both (Fig 17). This is presumably an indication that in spite of their similar sizes, perhaps the sampled *Eodromaeus* femur (PVSJ 561) came from a younger individual than the femora of *Chromogisaurus* (PVSJ 85) and/or *Eoraptor* (PVSJ 559). Moreover, *Chromogisaurus* and *Eoraptor* share intensive deep cortical secondary remodeling, but the *Chromogisaurus* femur lacks the transition to more organized peripheral primary bone characteristic of *Eoraptor*. These differences may be a signal that skeletal maturity was delayed in *Chromogisaurus* relative to *Eoraptor*.

We also detect some interesting similarities in generalized growth patterns when we consider distantly related taxa that share broadly similar ecological roles and/or functional biology. For example, three phylogenetically dissimilar taxa apparently adopted similar growth strategies of cyclical active growth, elevated bone depositional rates during intervals of active growth, and bone metabolism that fueled bone remodeling. *Exaeretodon* and *Hyperodapedon* fill large-bodied herbivorous niches and exhibit general similarities in femoral histology (Figs 8 and 9). Interestingly, they also share these patterns with *Saurosuchus*, a large-bodied carnivore (Fig 13). All three taxa are quadrupedal and exhibit large body sizes, which also may have played a role in their femoral histologies.

Our data further blur the distinctions in histological types that distinguish extant crocodile-line and bird-line archosaurs [e.g., 11, 15, 23, 24, 39–41, 60, 61, 101, 120, 121, 135, 152–157]. In modern systems, crocodile-line archosaurs generally exhibit more slow and cyclical growth throughout ontogeny, while many bird-line archosaurs grow faster and often more continuously [e.g., 1–18, 22–25, 27, 30, 31, 36–41, 46, 47, 155–157, 166]. Our data indicate that a diversity of crocodile-line archosaurs from the Ischigualasto Formation exhibit faster growth patterns than their living descendants. These results are consistent with those for many other Late Triassic crocodylomorphs [e.g., 51, 138, 139, 155–157], and add new data that help populate gaps in our understanding of key divergences in growth pattern among some of the earliest members of the crocodilian stem-lineage. *Sillosuchus* and *Saurosuchus* exhibit primary bone growth rates that are on par with those of relatively fast-growing extant mammals and birds [1–18]. The bone tissue we observed in the even more derived basal crocodylomorph *Trialestes* also departs from the slower growth patterns observed in extant crocodilians [1–18, 27]. The *Trialestes* cortex is completely dominated by continuous deposition of highly vascularized FLB throughout ontogeny. These new data indicate that *Trialestes* was growing faster than at least one of its larger-bodied relatives (*Saurosuchus*), and this may complicate the hypothesis that within archosaur clades larger species grew faster than smaller species, a pattern observed among some extant vertebrates [18, 157]. These divergent patterns, particularly amongst diverse members of the “crocodile-line” archosaur clade, highlight the need for more sampling and study of this group, which includes taxa that converged in body size, shape, and functional morphology with later Mesozoic theropod dinosaurs [e.g., 127, 136, 138, 139, 170, 171]. These data also bolster the hypothesis that at least some basal mid-sized terrestrial crocodylomorphs exhibited rapid rates of growth most similar to those recorded in the bones of mammals, and dinosaurs, including birds [1–18]. Moreover, the data generally support the hypothesis that basal Crocodylomorpha were experimenting with growth rates that were generally higher and were sustained for longer in ontogeny than those documented for crown group members of the clade [e.g., 1–18, 22–25, 27, 30, 31, 36–41, 46, 47, 155–157, 166].

Finally, it is interesting to note that the prevalence of skeletally immature specimens within the Ischigualasto assemblage is consistent with the expectations for the depositional settings of the Cancha de Bochas and Valle de la Luna Members of the Ischigualasto Formation [20]. Paleosols and paleofloristic data indicate that this interval was characterized by a seasonally arid climate [20, 72–76]. Periods of drought likely forced vertebrates to congregate around water sources, and likely also caused episodes of heightened mortality. Our data suggest that these conditions may have been particularly punishing for animals still actively growing and not yet at skeletal maturity, and this is consistent with mortality profiles documented in modern ecosystems impacted by droughts. It has also been hypothesized for other Triassic localities [59, 172–179].

### The dawn of a dinosaurian growth pattern?

Studies of Jurassic and Cretaceous dinosaurian growth patterns are common, and have revealed that all dinosaurs likely grew faster than extant reptiles [see recent summaries in 1, 2, 4–5, 166, 180]. Our understanding of this pattern diminishes as we delve deeper in time and approach dinosaur origins in the Late Triassic. Our data highlight the lack of a uniquely “dinosaurian” growth signal during this earliest phase of dinosaur evolution. The Ischigualasto dinosaur histologies are generally indistinguishable from those of taxa as diverse as *Proterochampsa*, *Trialestes*, and *Sillosuchus*, which come from distinctive, distantly related branches of the Late Triassic vertebrate phylogeny. The dinosaurs in our sample grew more continuously than some of the other large-bodied Ischigualasto taxa [e.g., *Saurosuchus*, *Exaeretodon*, *Hyperodapedon*], and they likely grew at more continuously rapid rates than the cynodont *Exaeretodon*. Because the Ischigualasto dinosaurs are the oldest known dinosaurs thus far, our results support the hypothesis that dinosaurs may have evolved elevated rates and continuous growth strategies characteristic of living birds and mammals very early in their history [1–2, 4–5, 166, 180]. That said, in doing so they were not alone. Our data on *Trialestes* and other non-dinosaurian archosaurs supports previous hypotheses that Triassic crocodile-line archosaurs exhibited a wider suite of growth strategies than those of their extant descendants [e.g., 1–18, 22–25, 27, 30, 31, 36–41, 46–47, 155–157, 166], and that these growth rates were, at least for some taxa, on par with those of the “fast growing” early dinosaurs in our sample.

The dense Haversian bone tissue signatures of intensive and deep cortical remodeling distinguish *Herrerasaurus*, *Eoraptor*, and *Chromogisaurus* from all other taxa in our sample, and may indicate both a more advanced relative ontogenetic age, as well as relatively high rates of bone metabolism [1–5, 56, 60–63]. Evidence of cortical remodeling also occurs throughout the cortex in *Saurosuchus* (Fig 13), where a single generation of secondary osteons reaches the outer cortex but never completely overprints primary tissue, even in the deep cortex. Secondary osteons are also present, but they are very sparse in the deepest cortices of *Exaeretodon* (Fig 8) *Hyperodapedon* (Fig 9), and *Proterochampsa* (Fig 10). Erosional rooms in the deep cortex of *Sillosuchus* are harbingers of future formation of secondary osteons. Given the ongoing nature of bone remodeling throughout life, we cannot rule out the hypothesis that dense Haversian tissue could form in these other taxa, but it seems unlikely given the relatively mature skeletons of at least some taxa [e.g., *Saurosuchus*, *Proterochampsa*) (Fig 20). The absence of similar patterns in *Eodromaeus* (Fig 17) and *Sanjuansaurus* (Fig 15) may be related to the relative skeletal immaturity our samples for both of these taxa may preserve. Thus, it is not the presence of secondary osteonal remodeling that distinguishes *Herrerasaurus*, *Eoraptor*, and *Chromogisaurus*, but the intensity and focused nature of preserved remodeling in their deep and mid-cortices (Figs 16, 18 and 19).

Our data suggest that the earliest known dinosaurs were already growing quickly and, continuously, for at least a significant fraction of their ontogenies. Though all of the Ischigualasto dinosaurs are members of GSG III, the group of fast and continuous growers, other early dinosaurs may not be. Our observations for these early dinosaurs contrast with at least some other Early Jurassic theropods and sauropodomorphs that instead exhibit the fast, but cyclical growth strategy shared by members of GSG II [17, 18, 166, 180–190]. Of course, our preliminary results require further testing via the analysis of new specimens, particularly of other basal dinosaurs and their contemporaries, especially those represented by ontogenetic series [26, 50, 54, 101, 144, 146, 173, 182–185]. With these goals in mind, new discoveries in the Santa Maria Formation in Brazil offer a prime opportunity to test these hypotheses in a similarly diverse fauna that is also temporally and paleoenvironmentally constrained [100, 101, 191–196]. Histological data from Santa Maria Formation aetosaurs [191] and the dinosaur *Nhandumirim* [193] support our conclusions that the interval surrounding the dawn of dinosaurs was a time of experimentation with fast growth a possibility across the phylogenetic spectrum.

## Conclusions

In this report we described the osteohistology of a diverse sample of tetrapods from the Upper Triassic Ischigualasto Formation of Argentina, including five early dinosaurs (*Chromogisaurus*, *Eodromaeus*, *Eoraptor*, *Herrerasaurus*, *Sanjuansaurus*). We approached the analysis of bone tissue from an “ecosystem-level” perspective by constraining our sample to femora drawn from a narrow temporal interval representing a specific paleoenvironment. These sampling controls pave a clearer path to comparison among disparate taxa. Our data allowed us to investigate two key questions: 1) What is the variability in femoral bone histology among members of this pivotal Late Triassic paleofauna? and 2) Do our histological results support the hypothesis that the oldest known dinosaurs already exhibited the elevated growth dynamics characteristic of later Mesozoic dinosaurs?

The Ischigualasto assemblage is dominated by skeletally immature individuals, but all sampled specimens exhibit characteristics of appreciable ontogenetic growth. Most Ischigualasto Formation taxa exhibit bone histological patterns consistent with relatively rapid growth rates for a significant part of their ontogenies. With the exception of the slower growing *Pseudochampsa*, growth rates among members of the paleofauna were likely on par with those observed in the tissues of some living mammals and birds, and generally exceeded those recorded for living reptiles. Diagnostic histological features allowed us to categorize our faunal sample into three distinctive Growth Strategy Groups that serve as a useful framework for future comparative studies. With these groups we recovered interesting variation in growth strategies among relatively closely related and morphologically similar taxa [e.g., *Proterochampsa* versus *Pseudochampsa; Herrerasaurus* vs. *Sanjuansaurus*), as well as among taxa that exhibit some similarities in apparent niche exploitation, in spite of being more distant relatives (e.g., *Hyperodapedon* and *Exaeretodon*). Similar patterns apparently also exist among Late Triassic taxa from other units, and these patterns may be characteristic of recovery faunas in the aftermath of the Permo-Triassic Mass Extinction.

Finally, the dinosaurs are among the fastest growing taxa in the sample, but they are not alone, sharing elevated growth rates with an archosauriform, a pseudosuchian, and a crocodylomorph. Interestingly, the Ischigualasto dinosaurs grew at least as quickly, and apparently more continuously than some basal sauropodomorph dinosaurs and even derived theropod dinosaurs of the later Mesozoic. Ancestral elevated growth rates likely played a significant role in the ascent of dinosaurs within Mesozoic ecosystems, but in the earliest days of their evolutionary history the dinosaurs were not alone in this regard: they shared this pattern with a diverse array of contemporaries.

## Supporting information

S1 File Source and copyright data for vertebrate silhouettes shown in Fig 21. (DOCX)

S2 File (DOCX)
