# Supplementary material for: Osteohistological insight into the growth dynamics of early dinosaurs and their contemporaries
Source: PLoS One. 2024 Apr 3;19(4):e0298242. doi: 10.1371/journal.pone.0298242 (PMC10990230; doi:10.1371/journal.pone.0298242)
Supplement: S1 File — (DOCX) [file pone.0298242.s001.docx]

Supporting Information

Osteohistological insight into the growth dynamics of early dinosaurs and their contemporaries

Kristina Curry Rogers^1^*, Ricardo N. Martínez^2^, Carina Colombi^3^, Raymond R. Rogers^4^, and Oscar Alcober^2^

Figure 21. Growth Strategy Groups Summary Figure. Silhouettes are all downloaded from phylopic.org. Artist details and copyright info for each silhouette as follows:

The following are copyrighted with <https://creativecommons.org/licenses/by/3.0/>

- *Chromogisaurus; Saurosuchus*, represented in Figure 20 by a related taxon, *Batrachotomus kupferzellensis*; and *Proterochampsa* represented in Figure 20 by the related taxon *Stangerochampsa*, were created by Scott Hartman.
- *Eoraptor* and *Pseudochampsa*, represented in Figure 20 by the related taxon, *Chanaresuchus bonapartei* are by T. Michael Keesey.
- *Trialestes*, represented in Figure 20 by the related taxon, *Terrestrisuchus gracilis,* was created by Scott Reid
- *Sillosuchus*, represented in Figure 20 by the related taxon, *Shuvosuarus inexpectatus*, was created by Sarah Werning,
- *Sanjuansaurus* was created by thefunkmonk,
- *Eodromaeus* was created by Dysalatornis

And the following are copyrighted with <https://creativecommons.org/publicdomain/zero/1.0/>

- *Exaretodon*, represented in Figure 20 by the related taxon *Lystrosaurus declivis,* was created by Matt Celeskey
- *Hyperodapedon* was created by Steven Traver
- *Herrerasaurus* was created by Tasman Dixon
